# Supplementary material for: Psilocybin-assisted massed cognitive processing therapy for chronic posttraumatic stress disorder: Protocol for an open-label pilot feasibility trial
Source: PLoS One. 2025 Jan 17;20(1):e0313741. doi: 10.1371/journal.pone.0313741 (PMC11741563; doi:10.1371/journal.pone.0313741)
Supplement: S1 File — (DOCX) [file pone.0313741.s001.docx]

**Letter of Information and Consent to Participate in a Research Study**

Before agreeing to take part in this research study, it is important that you read the information in this research consent form. It includes details we think you need to know in order to decide if you wish to take part in the study. If you have any questions, ask a study doctor or study staff.

| **Study Title:** | **Psilocybin-Assisted Massed Cognitive Processing Therapy for Chronic Posttraumatic Stress Disorder: An Open-label Trial** |
| --- | --- |
| **Protocol Number:** | [23-230] |
| **Sponsor Investigator/Lead Investigator (Study doctor):** | Venkat Bhat, MD MSc FRCPC DABPN  St. Michael's Hospital and the University of Toronto  193 Yonge Street, Suite 6-013, Toronto, ON M5B 1M8  416-360-4000 ext. 76404  [Venkat.bhat@utoronto.ca](mailto:Venkat.bhat@utoronto.ca)  [venkat.bhat@unityhealth.to](mailto:venkat.bhat@unityhealth.to)  24-hour emergency phone number: 647-946-4011 |
| **Co-Principal Investigator:** | Dr. Candice M. Monson, PhD  Professor of Psychology at Toronto Metropolitan University  416-979-5000 Ext. 556209  Email: [candice.monson@torontomu.ca](mailto:candice.monson@torontomu.ca) |
| **Co-Investigators:** | Dr. Perry Menzies  St. Michael's Hospital and the University of Toronto  416-864-3085  [perry.menzies@unityhealth.to](mailto:perry.menzies@unityhealth.to)  Dr. Rakesh Jetly, MD FRCPC  Associate Professor of Psychiatry at Dalhousie University and University of Ottawa  613-945-6817  [rakesh@drjetly.com](mailto:rakesh@drjetly.com)  Dr. Wendy Lou PhD  Professor of Biostatistics, Dalla Lana School of Public Health, University of Toronto  416-946-7804  [wendy.lou@utoronto.ca](mailto:wendy.lou@utoronto.ca)  Dr. Sridhar (Sri) Krishnan PhD, PEng, FCAE  Professor of Engineering, Signal Analysis Research Group, Toronto Metropolitan University  416-979-5000 Ext. 554931  [krishnan@ryerson.ca](mailto:krishnan@ryerson.ca) |
| **Address:** | St. Michael's Hospital, Unity Health Toronto  193 Yonge Street, Suite 6-013  Toronto, ON M5B 1M8 |
| **Date:** | 23-Feb-2024 |
| **Version:** | 5 |
| **Funder:** | Internal sources of funding |

**Conflict of Interest Statement**

The researchers are using internal funding from existing sources for this study and receive no direct or personal benefit from any study activities.

There is a risk of conflict of interest if you had previous relationships with any of the study staff. If that is the case, you may become ineligible to participate in the study.

Participation in this study is voluntary– you do not have to participate and you can withdraw at any time.

**Introduction**

You are being invited to consider taking part in a clinical research study testing an investigational drug for chronic posttraumatic stress disorder. You are being asked to take part in this study because you have chronic posttraumatic stress disorder. It is important that you understand the purpose of the study as well as what the study involves and its risks and possible benefits before you decide if you would like to participate. Please read this document carefully and take the time to consider if this study is right for you. You should take as much time as you need to make your decision.

One of our study team members will go through the information included in this consent form with you. Our study team members will explain what you will do during the research study, as well as risks and possible benefits. You should ask the study doctor or study staff to explain anything that you do not understand and make sure that all of your questions have been answered before signing this consent form. Before you make your decision, feel free to talk about this study with anyone you wish, including your family, friends and your healthcare professional(s).

Your participation is entirely voluntary, meaning that you may choose to take part or to not take part. Deciding not to participate in this study will **not** affect the regular health care you receive.

The study doctor will review your medical records, obtain additional history of your present illness, including detailed history of your prior and current treatments, duration and effects to determine if you are eligible to participate in the trial.

If you decide to take part, you will be asked to sign this consent form and will be given a copy to keep to show you agreed to participate. You are free to withdraw from the study at any time and do not have to give a reason for withdrawal. If you stop participating in the study, you would receive your normal standard of care.

1. **What is the purpose of the study?**

The purpose of this study is to see if psilocybin-assisted cognitive processing therapy is safe and well tolerated. Psilocybin-assisted cognitive processing therapy is a treatment approach that combines two elements. First, it involves the use of psilocybin, a psychedelic compound found in certain mushrooms, under controlled conditions with the guidance of trained professionals. Second, it incorporates cognitive processing therapy, which is a form of psychotherapy aimed at helping individuals process and make sense of traumatic experiences.

Researchers also want to know if psilocybin-assisted cognitive processing therapy can improve symptoms of chronic posttraumatic stress disorder. We will see if psilocybin-assisted cognitive processing therapy is safe and well tolerated by tracking changes in suicidal thoughts and behaviour, monitoring if any participants choose to stop participating in the study, and measuring any serious side effects, as well as how long they take to resolve. We will also see if posttraumatic stress disorder symptoms improve (or worsen) after psilocybin-assisted cognitive processing therapy is administered. Additional information about participants’ symptoms and side effects will also be measured during the study. At this time, the main purpose is to complete the study with 15 participants to learn whether it will be possible to plan for a larger study in the future.

**What is Psilocybin?**

Psilocybin is a controlled substance and a chemical compound found in some types of mushrooms. As an investigational drug, psilocybin cannot be prescribed for the treatment of posttraumatic stress disorder in Canada; however, Health Canada raised no objection to use of psilocybin in this research study.

Psilocybin is a “psychedelic” meaning that it creates an altered state of consciousness. The psychedelic experience is highly variable and may cause changes in perception, emotions, thoughts, feelings and social connectedness. Some may have an increased sensitivity to light and sound or an altered perception of time and colour. Profound experiences may occur, such as hallucinations, delusions, agitation, fearfulness, intense emotions, odd physical sensations, out of body experiences, the sensation that things are not real, entering a trance or dream like state or mystical experiences. Sometimes the experience can be scary, distressing and anxiety provoking. Some effects of psilocybin may include temporarily changing the way objects and people appear to you. For example, the size and shape of things can appear distorted, walls may appear to move as if they are made of fabric or as if they are ‘breathing’, shapes and colours may be seen on surfaces and the room may appear to get bigger or brighter. With your eyes closed, you may see shapes, colours, and unusual images as if you are dreaming. Time may appear to pass more slowly, and it may be hard to judge how much time has passed. Some people have reported visions of their pasts; for example, they might feel as if they are remembering or even reliving events from their childhood. These experiences normally resolve completely within six hours of ingesting psilocybin. Of note, these psychedelic effects might be important for the therapeutic effects. These psychedelic effects are believed to act as a catalyst to facilitate psychological shifts during psychotherapy to potentially change maladaptive thought patterns.

1. **Study Design and Duration**

**Study Design**

This is an open-label study, which means that both you and the study team know the dose and drug you are receiving. All participants in this study will receive a 25 mg dose of psilocybin. If you consent to participate in this study, you will also receive cognitive processing therapy. All study visits including therapy sessions and clinical assessments will be audio and video recorded. You will receive 12, 45 minutes cognitive processing therapy sessions, as well as a 45 minute preparatory session and a 1 hour integration session. Therapy sessions will be completed by one therapist who is a qualified mental health professional.

We are interested in the effects of psilocybin-assisted cognitive processing therapy on chronic posttraumatic stress disorder symptoms, but also other factors such as anxiety, depressive symptoms and quality of life. As such, questionnaires data will be done digitally through REDCap, a web-based platform that has fillable questionnaires for you to answer. During study visits, self-report and clinician-administered questionnaires will be completed to measure your symptoms.

Passive data will also be collected through a wearable device (Oura Ring).

**Oura Ring – The Wearable Device**

You will be required to use a wearable device (named “Oura Ring”) throughout the duration of your participation in this study. This device will consist of a ring which has its own mobile application (“Oura” app) that needs to be downloaded onto your smartphone from Google Play (Android) or the App store (Apple). The study team will provide you with a dummy email address and password that you will use to login to your Oura account. In this way, data collection on Oura Ring will be de-identified. Although the Oura app asks for your personal information when you first log in (e.g., sex, weight and height), **please DO NOT enter any personal information on the Oura app.**

The Oura Ring collects activity data and physiological signals (i.e., measurements related to body function such as heart rate or body temperature). The description of the passive data to be collected by Oura Ring is provided below as follows:

1. Sleep information: Oura Ring performs sleep analysis and stores a set of measurement parameters that summarize each period. The ring calculates the sleep period-specific parameters within four hours from the period end, but sleep analysis is always triggered when you open the application. These parameters include bedtime, total duration, awake, light, rapid eye movement (REM), and deep sleep durations, as well as heart rate, respiration rate, heart rate variability, and temperature changes.
2. Activity information: Activity summary contains daily activity summary values and detailed activity levels. Activity levels are expressed in metabolic equivalent of task minutes (MET mins). Oura tracks activity based on detected movement and presents the activity score based on duration of low, medium, and high activity levels. The movement is also used for step count and to estimate calories.
3. Readiness information: Readiness is interpreted from sleep, activity, resting heart rate, heart rate variability, recovery index and body temperature scores. A Readiness Score above 85% indicates that you're well recovered. A score below 70% usually means that an essential Readiness contributor, such as body temperature or previous night's sleep, falls outside your normal range, or clearly differs from recommended, science-based values.

**Participant Population and Study Enrollment**

**Who can take part in this study?**

Individuals must meet the following preliminary criteria to participate:

- Diagnosed with posttraumatic stress disorder by study psychiatrist;
- Duration of 6 months or longer of posttraumatic stress disorder;
- Are able to complete all protocol required assessment tools without any assistance or alteration to the assessments, and to comply with all study visits;
- Are in good physical health;
- Agree to not participate in any other clinical trials during this study.

If you agree to take part in this study, you will be screened according to further criteria to see if you are eligible to participate in the study.

**Participant Population and Study Enrollment**

This study will include 15 participants.

**Study Duration**

Overall, this study will run for about 17 weeks. Your involvement in this study will last for about 17 weeks.

1. **Description of Study Visits**

**What happens at the study visits?**

Participation in the study will involve a minimum of 25 visits (4 in-person, 21 virtual) and will last approximately 17 weeks.

Before you begin the study:

Research studies have strict requirements about who can join to make it safer for participants and the study results reliable. You will complete some tests and procedures to make sure that you meet the specific requirements of this study. You may find out that you are not eligible to take part. If you are found to not be eligible for this study, the study doctor will discuss alternative treatments with you.

During the screening visit, the following tests will be done:

Clinical and Psychiatric Assessments:

The research staff will ask you questions and you will be asked to complete standardized questionnaires about your health, medical history, medications, and mood. They will also ask you a set of interview questions that will assess and evaluate your current and past psychiatric history. In addition, the research staff will also record your height, weight, blood pressure, and heart rate.

Lab Tests:

In addition to the above eligibility assessments, you will also be asked to undertake lab tests prior to commencing the study. These samples include blood and urine samples as well as an electrocardiogram (ECG). Blood samples will be collected and processed at St. Michael's Hospital during your first screening visit. Approximately 20 mL (about 4 teaspoons) of blood will be taken for the blood sample. The results of the bloodwork will be used to determine if it is safe for you to participate in this study.

A urine sample will also be collected during your first screening visit. Urine samples are taken for routine tests and used to check for drug use and, for females, to check for pregnancy.

An ECG is a non-invasive test that examines the electrical activity of your heart. It is conducted by placing small electrodes on your body. You will undergo this test at your first screening visit.

The results of the bloodwork, urine sample, and ECG will be reviewed by the study psychiatrist to ensure that it is safe for you to participate in the study. There is a possibility that the routine tests may show something outside of the usual range.

A summary of activities at each visit is described below.

| Study visit | Timeline | Location | Main events | Duration |
| --- | --- | --- | --- | --- |
| Screening | ~ 4 weeks before baseline | Study center | 1. Medication review  2. Completion of questionnaires  3. Vital signs* and weight measured  4. Physical examination  5. Laboratory tests and electrocardiogram (ECG) | 2-3 hours |
| Eligibility confirmation | ~ 3 weeks before baseline | Study center | 1. Medication review  2. Laboratory tests result review  3. Oura ring | 1 hour |
| Pre-baseline wearable monitoring (Oura) (week 1) | ~ 3 weeks before baseline | Virtual | 1. Current medication review  2. Completion of questionnaires  3. Safety assessment | 1 hour |
| Pre-baseline wearable monitoring (Oura) (week 2) | ~ 2 weeks before baseline | Virtual | 1. Current medication review  2. Completion of questionnaires  3. Safety assessment | 1 hour |
| Pre-baseline wearable monitoring (Oura) (week 3) | ~ 1 weeks before baseline | Virtual | 1. Current medication review  2. Completion of questionnaires  3. Safety assessment | 1 hour |
| Baseline | One day before start of treatment week | Virtual | 1. Medication review  2. Completion of questionnaires | 1-2 hours |
| CPT sessions | Day 1 of treatment week | Virtual | 1. Medication review  2. Completion of questionnaires  4. 90 minutes CPT sessions 1 and 2 | 2-3 hours |
| Preparatory (session where participant get information about the study, discuss their health and expectations before they actually use psilocybin) and CPT sessions | Day 2 of treatment week | Virtual | 1. Medication review  2. Completion of questionnaires  3. 45 minutes preparatory session  4. 90 minutes CPT sessions 3 and 4 | 3-4 hours |
| Psilocybin dosing session | Day 3 of treatment week | Study center | 1. Current medication review  2.Completion of questionnaires  3.Vital signs and weight measured  4.Safety assessment  5. **Psilocybin dosing session**  6. Therapy | 6-8 hours |
| Integration and CPT sessions | Day 4 of treatment week | Virtual | 1. Medication review  2. Completion of questionnaires  3. 1 hour integration session  4. 90 minutes CPT sessions 5 and 6 | 3-4 hours |
| CPT sessions | Day 5 of treatment week | Virtual | 1. Medication review  2. Completion of questionnaires  3. 90 minutes CPT sessions 7 and 8 | 2-3 hours |
| CPT sessions | Day 6 of treatment week | Virtual | 1. Medication review  2. Completion of questionnaires  3. 90 minutes CPT sessions 9 and 10 | 2-3 hours |
| CPT sessions | Day 7 of treatment week | Virtual | 1. Medication review  2. Completion of questionnaires  3. 90 minutes CPT sessions 11 and 12 | 2-3 hours |
| Follow-up (week 2) | 1 week after treatment week | Virtual | 1. Completion of questionnaires  2. Safety assessment | 1 hour |
| Follow-up (week 3) | 2 week after treatment week | Virtual | 1. Completion of questionnaires  2. Safety assessment | 1 hour |
| Follow-up (week 4) | 3 week after treatment week | Virtual | 1. Completion of questionnaires  2. Safety assessment | 1 hour |
| Follow-up (week 5) | 4 week after treatment week | Virtual | 1. Current medication review  2. Completion of questionnaires  3. Safety assessment | 1-2 hours |
| Follow-up (week 6) | 5 week after treatment week | Virtual | 1. Completion of questionnaires  2. Safety assessment | 1 hour |
| Follow-up (week 7) | 6 week after treatment week | Virtual | 1. Completion of questionnaires  2. Safety assessment | 1 hour |
| Follow-up (week 8) | 7 week after treatment week | Virtual | 1. Completion of questionnaires  2. Safety assessment | 1 hour |
| Follow-up (week 9) | 8 week after treatment week | Virtual | 1. Completion of questionnaires  2. Safety assessment | 1 hour |
| Follow-up (week 10) | 9 week after treatment week | Virtual | 1. Completion of questionnaires  2. Safety assessment | 1 hour |
| Follow-up (week 11) | 10 week after treatment week | Virtual | 1. Completion of questionnaires  2. Safety assessment | 1 hour |
| Follow-up (week 12) | 11 week after treatment week | Virtual | 1. Completion of questionnaires  2. Safety assessment | 1 hour |
| Follow-up (week 13) | 12 week after treatment week | Study center | 1. Current medication review  2. Completion of questionnaires  3. Safety assessment | 1-2 hours |

*Vital signs: blood pressure, body temperature, pulse

Your participation in this study will last a total of 17 weeks. You will be asked to come to St. Michael’s Hospital for screening, eligibility confirmation, psilocybin dosing session and week 13 follow up. The therapy sessions and other study assessments will be completed remotely. On the eligibility confirmation visit, you will be given the Oura Ring and a member of the research team will assist you in the process of setting it up for usage. You will begin using the Oura Ring immediately after the eligibility confirmation visit. You will consistently use the Oura Ring throughout the study.

What can I expect on the day I receive psilocybin (Day 3)?

On the morning of the intervention, you should have a light breakfast 2 hours before your appointment. The intervention usually takes the whole day. You will receive the intervention in a calm environment, have eye-shades, and listen to relaxing music playing quietly for most of the drug experience. The effects of psilocybin usually start around 30 minutes after taking the capsules, become most intense in the first 2 hours, and then gradually subside. There will be a study therapist throughout the intervention and will be in the dosing room with you at all times. You will be asked to stay for a minimum of 5 hours, even though your experience may last less time than this.

At the intervention, the following will also be done:

- Your body temperature, blood pressure and heart rate will be checked
- Light snacks will be available during the day. You are welcome to bring a light lunch and/or snacks with you to the dosing session.
- Towards the end of the session, you will complete questionnaires asking about your experience

At the end of the intervention day and when the study team has confirmed it is safe for you, you will be released to home in the care of a family member or friend. It is important that a responsible adult accompanies you home and stays with you for at least 24 hours after you have received the intervention. The study team would like to have this person’s telephone number to ensure that we can get in contact with you that evening.

After leaving and for up to 24 hours after you received the intervention, do not operate heavy machinery, gym equipment, a motor vehicle, or bicycle. We would rather you did not go out, but instead have a relaxing time at home. Please do not drink alcohol or use medications not provided by the study team, or illicit or recreational drugs.

You will be given contact numbers in case you wish to get in contact with us, or someone who has an awareness of the study, at any time.

1. **Participant Responsibilities**

**What are my responsibilities as a study participant?**

You will be responsible for:

- Showing up for all study visits, on time;
- We will require you to discontinue some of your regular medications to participate in the study. Some common medications that we will require you stop taking include, for example, antidepressants, psychostimulants, and antipsychotics. The decision to stop taking your regular medications to participate in this study should be made by you and study psychiatrist.
- You must not use illicit substances (e.g. cocaine, ecstasy/MDMA, hallucinogens) for the during of the study (this will be checked with a urine screening);
- You must not use cannabis one week before and one week after psilocybin dosing session;
- You must not use alcohol for 14 hours before and 24 hours after psilocybin dosing session;
- You must not use benzodiazepines, hypnotics, and mood stabilizers for 12 hours before and 12 hours after psilocybin dosing session;
- Inform the study coordinator of all events that you think might affect your participation in the study;
- If you become pregnant or get someone pregnant while taking the study drug or afterward, you should immediately notify the study investigators, who will discuss next steps with you;
- Participants who are able to become pregnant or produce sperm must agree to both of the following while taking the study drug and for the length of time afterward: i) not to get pregnant or get someone pregnant and ii) to use an appropriate family planning method as discussed and decided upon in consultation with a study investigator;
- You must not drive or operate machinery for 24 hours after psilocybin dose;
- Upon discharge from the study setting, you must be discharged to the care of a responsible individual (i.e., family member, friend, chaperone) who can escort you home and observe you for the remainder of 24 hours after the dose was administered;
- Complete questionnaires when asked to do so;
- Inform the study team if anything about your health, prescription or non-prescription medications (e.g. vitamins and herbal supplements) has changed. This is for your safety.

1. **Potential Risks**

**Are there any risks of participating in this study?**

This study has risks. Some of these risks we know about. There is also a possibility of risks that we do not know about that have not been seen in study participants to date. Please call the study doctor if you have any side effects even if you do not think they have anything to do with this study. The risks we know of are:

**Risks of Psilocybin**

Although no serious side effects have been reported to date, there is insufficient data to rule out the possibility of serious side effects. The numbers in brackets show how often the side-effect was reported.

Common (over 50%):

1) Visual and other sensory distortions, feeling of unreality & changed sense of time

2) Anxiety at the onset of the drug effects

3) Increased heart rate and blood pressure

Less common (about 10-40%):

1) Nausea

2) Dizziness

3) Blurred vision

4) Drowsiness

5) Sleepiness

6) Headache

7) Temporary suspiciousness

Rare (less than 10%):

1) Transient recurrence of disturbances in perception that are reminiscent of those experiences during one or more earlier hallucinogen intoxications. This side effect is seen rarely with recreational use of psilocybin, and has not been reported in scientific studies done under supportive clinical conditions.

2) Worsening of mental state after psilocybin experience (very rare and not seen in similar studies).

It is essential to be informed of the potential risk of experiencing suicidal ideation and other serious adverse events related to the use of psilocybin. Although psilocybin has demonstrated therapeutic potential in various mental health conditions, it can also induce intense emotional and psychological effects. Some individuals may encounter heightened introspection and emotional sensitivity during the psilocybin sessions, which could lead to the emergence of distressing thoughts, including suicidal ideation, intentional self-injury, and even hospitalization in severe cases. You must be aware of this risk and understand the significance of open communication with the research team throughout the study. Strict safety protocols and regular monitoring will be in place to promptly identify and address any adverse reactions, ensuring your welfare and safety are prioritized at all times.

**Risk of drug-drug Interactions**

Psilocybin may interfere with medications, both prescribed and over the counter, that you might be taking. Psilocybin may also affect the way some medications and vaccinations work. Consult with your study doctor about whether the study drug may interfere with your medications as well as before you start taking any new medications.

We will require you to discontinue some of your regular medications to participate in the study. Some common medications that we will require you stop taking include, for example, antidepressants, psychostimulants, and antipsychotics.You will be asked to stop taking certain types of medication used to treat your depressive symptoms for a minimum period of 2-weeks, before baseline visit. The decision to stop taking your regular medications to participate in this study should be made by you and the study psychiatrist. The study psychiatrist will discuss the options of tapering off your medications with you and your healthcare provider. You will be given a choice of how quickly to come off the medications, but you will need to be off the medication for at least 2 weeks prior to baseline visit. Withdrawing from these medications can be challenging for some patients. One of the significant concerns is the possibility of experiencing withdrawal effects, such as nausea, headaches, dizziness, insomnia, and mood disturbances, which can cause discomfort and distress. Abruptly stopping psychotropic medications may also lead to a relapse of the underlying mental health condition, worsening symptoms, and affecting your overall well-being. It is crucial to consider the potential impact on your daily life, including work, relationships, and social activities, when making decisions about your participation in the trial. Additionally, discontinuation could increase the risk of suicidal thoughts or behaviors and trigger psychiatric decompensation, emphasizing the importance of close monitoring during the transition period. Please note that the risks and benefits of this decision will be thoroughly discussed with you to ensure your well-being throughout the trial. The study team and psychiatrist will support you during the period of medication withdrawal. If at any point you want to withdraw from the study and go back on your usual medication, you will be free to do so.

It is important to tell the study doctor right away if you have any problems with stopping or changing your regular medications.

**Possible Risks of Withdrawing from Medications:**

Withdrawing from medications can be challenging for some patients. Symptoms of withdrawal could include difficult sleeping, nausea, flu-like symptoms, and jitters. While these symptoms are generally not dangerous and usually pass in a few days, it is crucial to note that withdrawal from benzodiazepines or alcohol can be potentially hazardous and may require medical supervision. The study team and clinician will support you during the period of medication withdrawal. You will be seen for weekly appointments with the study psychiatrist during the tapering period to check how you are doing. If at any point you want to withdraw from the study and go back on your usual medication, you will be free to do so.

**Possible Risks of the Electrocardiogram (ECG):**

Skin irritation from the ECG electrode pads or pain when removing the sticky pads are possible side effects.

**Possible Risks of Blood Draws:**

The risks and discomforts of giving a blood sample are the same as those for any blood taken from a vein. There is a possibility of pain, bruising, swelling, or infection when giving blood. These discomforts usually go away quickly. In rare cases there may be local infection.

**Risk of Questionnaires**

There is a risk that you may feel uncomfortable discussing some of the topics in the clinical assessment and completing some of the questionnaires. You can skip questions that you do not wish to answer, or you can pause or stop your participation in the study. If you have any questions or concerns while answering these questions, the study team is available to discuss your concerns and/or to refer you to appropriate resources.

**Risk of Wearable Device (Oura Ring)**

If you agree to participate, you will be asked to use the wearable device and its app throughout the study. You may experience a burden or a negative reaction when using the wearable device and its app as you will be asked to wear the device whenever possible and to recharge it when necessary (approximately every 5-6 days). You may also experience negative feelings or reactions when accessing your data on the wearable app. If any of these things happen, you can pause or stop using the wearable device and wearable app, or you can also choose to withdraw from the study. If you choose to pause or stop your use of the wearable device and app, please inform a member of the study team. Additionally, you may have an allergic reaction while using the wearable device; in this case, you should stop using the device and contact your family physician. Please avoid handling batteries, or working on devices with machinery that contain batteries while wearing your Oura Ring. In certain cases, where both the cathode and the anode of another battery touch the ring, there is a risk of a short circuit which is similar to standard metallic rings. This can result in a potentially dangerous shock. Most importantly, if you experience a negative reaction or distress, you may contact the Study Coordinator, as they are available to answer any questions you might have regarding the study, the wearable device and its app, or where to seek assistance if needed. If you need immediate assistance, please call 911 and go to the Emergency Room closest to you.

1. **Risks Related to Pregnancy**

Psilocybin may be harmful to a fetus or sperm. Participants should not breastfeed while in this study because the drugs used in this study might be present in breast milk and could be harmful to a baby. Currently, we do not know all of the risks related to pregnancy or nursing while using psilocybin.

The effects that psilocybin may have on eggs (ova), sperm, or on an unborn baby (fetus) are unknown. You should not become pregnant or get someone pregnant while taking psilocybin. Participants who are able to become pregnant or produce sperm must agree to both of the following while taking psilocybin and for length of time afterward: i) not to get pregnant or get someone pregnant and ii) to use an appropriate family planning method as discussed and decided upon in consultation with a study investigator.

If you become pregnant or get someone pregnant while taking psilocybin or for length of time afterward, you should immediately notify the study investigator, who will discuss next steps with you. If you are able to become pregnant, the study investigator will order a blood/urine pregnancy test prior to the start of your participation in this study to confirm that you are not pregnant. To confirm that you have not become pregnant during the study, blood/urine pregnancy tests will be done throughout your participation in the study.

If you become pregnant or get someone pregnant while you are taking psilocybin, the study team may ask if you/the person who is pregnant would be willing to provide information about the pregnancy as part of this study. A separate consent document will be used to request permission to collect this information. You/The person who is pregnant may choose not to give consent for the collection of this information or may withdraw consent at any time without giving a reason. This decision will not affect your participation in this study and will not affect the health care that any person receives at Unity Health Toronto.

You should not nurse (breastfeed or chestfeed) an infant while in this study because psilocybin may be present in your milk and could be harmful to a nursing infant.

1. **Are there any benefits of participating in this study?**

You may or may not receive any direct benefit from being in this study. You may experience a decrease in symptoms of chronic posttraumatic stress disorder, but this is not guaranteed. It is possible that symptoms of chronic posttraumatic stress disorder may worsen while you are participating in this study. Information learned from this study may help other people with posttraumatic stress disorder in the future.

1. **What is my alternative to being in this study?**

You may opt to not participate in this study. You do not have to take part in this study to receive treatment for your condition. A number of other medications are available. The study doctor will discuss these alternative treatments with you, including their important potential risks and benefits.

1. **Privacy and Confidentiality of Your Personally Identifying Information and Study Data**

All persons involved in this study are committed to respecting your privacy. Other than the individuals or groups described in this section, no persons will have access to your personally identifying information without your consent, unless required by law. Personally identifying information is any information that could be used to identify you; this includes your name and address. Study data is information that is generated by and/or collected for a study that has been stripped of personally identifying information.

**Protecting Your Privacy**

The study team will make every effort to keep your personally identifying information private and confidential in accordance with all applicable privacy legislation, including the Personal Health Information Protection Act (PHIPA) of Ontario.

In addition to the study team, other authorized employees of Unity Health Toronto may have access to your personally identifying information so that they can carry out regulatory or institutionally required duties. Unity Health Toronto may also store personally identifying information that is collected or used for these duties for a period of time, in accordance with regulations and institutional policies. No personally identifying information will be allowed off site in any form, unless required by law or as described in this consent form.

**Medical Records**

**Accessing and collecting from your Unity Health Toronto medical records**

By signing this form, you are authorizing access to your medical records by the study personnel. The study team will also collect information from your medical record. The study personnel will use this information to conduct the study.

You are also authorizing access to your medical records by representatives of the Unity Health Toronto Research Ethics Board and by Health Canada. Such access will be used only to verify the authenticity and accuracy of the information collected for the study, without violating your confidentiality, to the extent permitted by applicable laws and regulations.

**Accessing and collecting information from your medical record at other institutions or providers**

By signing this form, you are giving us permission to access your medical records held by other institutions or health care providers. These other institutions or providers may ask you to give separate consent to allow them to release your medical information to us. The information that will be collected from other institutions or providers is described in the Description of Study Visits section. The study team will use this information to conduct this study.

**Will my participation in this study be kept confidential?**

Personal Health Information

If you agree to join this study, the study doctor and his study team will collect your personal health information that they need for the study. Personal health information is any information that could be used to identify you and includes your:

- Name
- Address
- Date of Birth
- New or existing medical records, which includes types, dates and results of medical tests or procedures.

Your research records will be kept for 25 years after study completion at St. Michael’s Hospital in a highly secure and confidential manner, as this is the length of time required by Health Canada. The information will then be anonymized (any link to you will be destroyed) or completely destroyed.

The study team can tell you what information about you will be stored electronically and may be shared outside of the study team.

The following people may look at the study records and at your personal health information to check that the information collected for the study is correct and to make sure the study followed proper laws and guidelines:

· The study sponsor or its representatives/partner companies.

· Representatives of the institutional review board (IRB) Services - an independent ethics committee that reviewed the ethical aspects of this study to help protect the rights and welfare of study participants.

· Representatives of Health Canada, or other regulatory bodies (groups of people who oversee research studies), outside of Canada, such as the United States Food and Drug Administration.

**Study Information That Does Not Identify You**

Some study information will be sent outside of the study team. De-identified safety data will be submitted to the manufacturer.

Any information about you that is sent out will have a code and will not show your name or address, or any information that directly identifies you. The code, with which your data is identifiable, will be stored for a minimum of 15 years.

All identifiable information collected during this study, including your personal health information, will be kept confidential and will not be shared with anyone outside the study unless required by law. You will not be named in any reports, publications, or presentations that may come from this study.

If you decide to leave the study, the information about you that was collected before you left the study will still be used. No new information will be collected without your permission.

**Are there any risks of using email for research study?**

There are common risks of using email and/or texting to communicate:

- Information travels electronically and is not secure in the way a phone call or regular mail would be.
- If someone sees these emails and/or texts, they may know that you are a participant in this study or see the health information included in the email and/or text.
- Emails and/or texts may be read or saved by your internet or phone provider (i.e., Rogers, your workplace, “free internet” providers).
- Copies of an email and/or text may continue to exist, even after efforts to delete the email and/or text have been made.
- There is always a chance with any unencrypted email and/or text, however remote, that it could be intercepted or manipulated.

Do not use email and/or text messaging for medical emergencies. If you require immediate help, call your clinic or care provider, or seek emergency services.

**Personally Identifying Information Storage and Retention**

All your personally identifying information will be collected on a database for analysis. The data will be stored on a secure internal hospital server and will be password protected so that it is only accessible to authorized research personnel.

Your personally identifying information will be kept by the Principal Investigator and Unity Health Toronto for 15 years after study completion at St. Michael’s Hospital in a highly secure and confidential manner, as per hospital requirements. After 15 years, any documents with personally identifying information will be destroyed.

**Study Data Storage and Retention**

As a reminder, study data is information that is generated by or collected for a study that has been stripped of personally identifying information. Study data will be securely stored at Unity Health Toronto. Study data may also be transferred outside of Unity Health Toronto and shared with others for purposes related to the conduct of this study. Study data may be kept indefinitely and may be used for other research or analyses by the study investigators and the study sponsor. Individual level study data may also be made available to scientific journals, their reviewers, other researchers inside or outside of Unity Health Toronto, or the public.

1. **Study Results and Registration**

**Results**

The results of this study may be presented at a scientific conference or published in a scientific journal. If you are interested in obtaining the results of this study, you can contact the study team. We estimate that the results of this study will be available in 1 year.

You will never be personally identified in any publication, report, or presentation that may come from this study.

**Registration**

A description of this clinical trial will be available on [http://www.ClinicalTrials.gov](http://www.clinicaltrials.gov). This website will not include information that can identify you. At most, the website will include a summary of the results. You can search this website at any time.

The registration number for this study is _____.

1. **Potential Costs and Reimbursement**

**Will I have to pay to participate in the study?**

There are no costs to you for participation in this study. You will not have to pay for any of the procedures involved in this study or for the study drug or therapy sessions.

**Will I be compensated for participating in the study?**

You will be compensated up to $30 per in-person visit to cover all travel expenses, but we are not able to provide compensation for additional expenses such as food. You will receive cash at the end of each in-person visit, and no receipts will need to be provided. If you withdraw from this study before completing it, you will receive compensation for the parts of the study that you have completed.

If a discovery is made or a commercial product or method is derived from this study, it will be the property of the study sponsor and you will not be entitled to any financial benefits resulting from it.

1. **What if I am hurt during my participation in the study?**

If you experience side effects from study interventions or study procedures, you should inform the study team as soon as possible. If you have any other concerns about safety or unexplained symptoms, contact us immediately.

**Compensation for injury:**

If you are injured because of your participation in this study, medical care will be provided to you in the same manner as you would ordinarily obtain any other medical treatment. In no way does signing this form waive your legal rights nor release the study doctor(s), sponsor, or involved institution(s) from their legal and professional responsibilities.

1. **Participation and Withdrawal**

**Participation in the study**

Your participation in this study is voluntary. If you choose not to participate, there will be no impact to the medical care received at, or other relationship with Unity Health Toronto now or in the future for you or your family.

**Withdrawal from the study**

You do not have to participate in this study. Your participation in this study is voluntary. If you choose to take part in this study, you can change your mind without giving a reason, and you may withdraw from this study at any time without any effect on the medical care, employment or other relationship you or your family have at or with Unity Health Toronto. You may decide not to be in this study, or you may decide to be in this study now and then change your mind (i.e., “withdraw”) at a later date. You may withdraw from this study at any point for any reason, or for no reason. If at any time you choose to withdraw from this study, please contact a member of the study team. If you withdraw from the study due to side effects, it is important to tell the study doctor.

You may be asked to stop participating in this study at any time by the study doctor, Sponsor, or regulatory body, without your consent, for any reason. Some reasons for this may be:

- If continuation in this study appears to be harmful to you
- If it is discovered that you do not meet the eligibility requirements
- You do not follow study instructions
- You need medicine that is not allowed while participating in this study
- You become (or get someone) pregnant during the study
- You have a serious or severe side effect
- The study doctor thinks it is in your best interest to stop participating in the study

If you are withdrawn from this study or if this study ends early, a study team member will discuss possible next steps with you.

**Continued collection and use of your data after withdrawal**

If you withdraw or are withdrawn from the study, no more data about you will be collected unless it is necessary to follow up on an adverse event that is not resolved at the time of your withdrawal. Any data collected from you up to that time will still be used for analysis. We may be required to retain the personally identifying information and study data that we have already collected until after the study (described in the Privacy and Confidentiality section).

Any data that has been added to your medical record be deleted from the record or withdrawn

Continued use of your samples after withdrawal

If you withdraw or are withdrawn from this study, data from samples that have already been analyzed will still be used. No further analysis will be done on your samples and any remaining samples will be destroyed.

After the Study

You will not have access to the study intervention after your involvement in the study.

1. **New Information about the Study**

We may make changes to the study. We may also learn new things about the study that you may need to know. Some of the new information or changes might affect your decision to take part in the study. If so, you will be notified about the new or changed information in a timely manner and we will ask you if you consent to remain in the study. You may be asked to sign a new consent form at that time.

1. **New Information about Your Health (Incidental findings)**

The tests or procedures that we conduct during this study might reveal medical information about you that is not part of the objectives of this study but may be relevant to your health. This type of medical information is called an incidental finding. Some incidental findings could be related to treatable conditions, or they could be related to factors that may affect your current or future health care. With your consent, we will communicate all medically actionable incidental findings to you.

1. **Study Contacts**

**Who do I contact about this study?**

If you have questions about taking part in this study, or if you suffer a research-related injury, you can talk to the research team, or the person who oversees the study at this institution. That person is:

**Venkat Bhat, MD, MSc, FRCPC, DABPN**

St. Michael’s Hospital and University of Toronto

24 Hour Contact: 416-360-4000 ext. 76404

(You will be asked to leave your phone number to be called)

**In case of an emergency, please go to the nearest hospital emergency department.**

**Research Ethics Board Contact**

If you have any questions regarding your rights as a research participant, you may contact the Unity Health Toronto Research Ethics Board Office at:

**Unity Health Toronto Research Ethics Board**

Monday to Friday, 9:00 a.m. to 5:00 p.m.

416-864-6060 ext. 42557

Unity Health Toronto is a health network that includes Providence Healthcare, St. Joseph’s Health Centre, and St. Michael’s Hospital.

The Unity Health Toronto Research Ethics Board is made up of a group of scientists, medical staff, and individuals from other backgrounds (including law and ethics) as well as members from the community. The Board was established by Unity Health Toronto to review studies for their scientific and ethical merit. The Board pays special attention to the potential risks and benefits to the research participant, as well as the potential benefit to society.

**You will be given a signed copy of this consent form.**

1. **Who is this study being organized and sponsored by?**

This study will be conducted with internal sources of funding. Psilocybin in kind contribution of Mydecine Innovations Group Inc.

**Signature Pages: Documentation of Informed Consent**

| **Study Title:** | **Psilocybin-Assisted Massed Cognitive Processing Therapy for Chronic Posttraumatic Stress Disorder: An Open-label Trial** |
| --- | --- |
| **Sponsor Investigator/Lead Investigator:** | Venkat Bhat, MD, MSc, FRCPC, DABPN  416-360-4000 ext. 76404 (24-hour contact) |

**Participant Statements of Consent**

Information regarding your Assessment and Treatment sessions using Zoom

The participant understands the following terms of use:

(a) You have read and understood these instructions for use of the Zoom application.

(b) In order to participate fully you are required to have access to the following equipment:

a. High-speed internet

b. Personal device - computer, tablet or smartphone with a microphone/speaker

c. Webcam (if not built-in to device)

(c) Technical difficulties may occur at times. Your assessor, therapist, or research coordinator will try and resolve the issue with you. If this fails, the session may take place via phone, or will be rescheduled for a later date and time.

(d) Tips for improve the quality of the virtual session:

a. Reduce the number of devices streaming at the time of your session.

b. Place your device on a stable surface.

c. Sit near your modem.

d. Turn on the lights.

e. Using headphones can both increase privacy and reduce echo.

(e) For safety reasons, the treating clinician must know where you are physically located. If your physical location is not the location the research study has on file, please inform the therapist of your location [KS1] .

(f) You need to be located in Ontario when you connect to your virtual session. Sessions cannot be conducted while you are out of the province.

(g) You are asked to participate in the virtual sessions as if they are being held in person. Protect the session time so that you are not interrupted or distracted, and do not engage in behaviours that you would not do during an in-person session (e.g., smoking, texting, cooking, walking around, etc).

(h) You shall not directly record the session in any way including by audio device, video device, photographing, or any other electronic device. You will be informed if the session is being recorded by the assessor or treating clinician.

(i) No other person(s) may be invited to view the Zoom session, unless that person is invited by the health care professional conducting the session, in consultation with the participant. You must be alone in the room during the sessions unless otherwise agreed upon.

(j) The participant understands and accepts the risks of participating in a Zoom session, including the risk that personal health information transmitted via Zoom may be accessible by an unauthorized user.

I have review and understand the Zoom application information provided above:

Name: __________________________________

Date: __________________________

[KS1] is this something we are collecting? If not, the therapist should ask at the first session.

**Consent for touch during therapy**

Informed consent for body and touch-oriented approaches within therapy is essential and out of respect for your right to choice and self-determination. Consent must be given voluntarily, knowingly and intelligently. Consent is active and you can change your mind at any time.

◻ Yes ◻ No I consent to touch during therapy

By signing this consent form, I acknowledge that:

- I understand that permission was given for me to participate in this study by my Substitute Decision Maker while I was unable to make my own decisions.
- This research study has now been explained to me, and my questions have been answered to my satisfaction.
- I have been informed of the alternatives to participation in this study.
- I know that I have the right not to continue participating in this study and the right to withdraw from this study without affecting the medical care received at, employment at, or other relationship with Unity Health now or in the future for me and my family.
- The potential risks and benefits (if any) of participating in this study have been explained to me.
- I have been told that I have not waived my legal rights nor released the study investigator, study sponsor, or involved institutions from their legal and professional responsibilities.
- I know that I may ask, now or in the future, any questions I have about this study.
- I have been told that information about me and my participation in this study will be kept confidential and that no personally identifying information will be disclosed without my permission unless required by law.
- I have been given sufficient time to read and understand the information in this consent form.
- I will be given a signed and dated copy of this consent form.

At this time, I am now able to make my own decisions and (initial as decided):

|  | YES, I agree to allow my collected study data to remain part of this study. |
| --- | --- |
|  | NO, I do not consent to allow my collected study data to remain part of this study |

If this study may reveal incidental findings:

Please initial one of the boxes below to indicate whether or not you want to be informed of all medically actionable incidental research findings.

|  | YES, I agree to be told about any medically actionable incidental findings. |
| --- | --- |
|  | NO, I do not want to be told about medically actionable incidental findings. |

|  |  |  |  |  |  |  |
| --- | --- | --- | --- | --- | --- | --- |
| Participant name (print) |  | Participant signature |  | Date |  | Time |

I have explained to the above-named participant the nature and purpose, the potential benefits, and possible risks of participation in this study. All questions that have been raised about this study have been answered.

|  |  |  |  |  |  |  |  |  |
| --- | --- | --- | --- | --- | --- | --- | --- | --- |
| Name of person obtaining consent (print) |  | Position/Title of person obtaining consent (print) |  | Signature of person obtaining consent |  | Date |  | Time |
