# Supplementary material for: Psilocybin-assisted massed cognitive processing therapy for chronic posttraumatic stress disorder: Protocol for an open-label pilot feasibility trial
Source: PLoS One. 2025 Jan 17;20(1):e0313741. doi: 10.1371/journal.pone.0313741 (PMC11741563; doi:10.1371/journal.pone.0313741)
Supplement: S2 File — (DOCX) [file pone.0313741.s002.docx]

**Clinical Study Protocol**

**Psilocybin-Assisted Massed Cognitive Processing Therapy for Chronic Posttraumatic Stress Disorder: An Open-label Trial**

| **Investigational Product:** | Psilocybin |
| --- | --- |
| **Indication:** | Chronic Posttraumatic Stress Disorder |
| **Study Design:** | Study type: Pilot open label trial  Allocation: N/A  Intervention model: Single arm  Primary outcome: Feasibility |
| **Principal Investigator:** | Dr. Venkat Bhat, MD MSc FRCPC DABPN  St. Michael's Hospital and University of Toronto  416-360-4000 Ext. 76404  v[enkat.bhat@utoronto.ca](mailto:Venkat.bhat@utoronto.ca) |
| **Co-Principal Investigator:** | Dr. Candice M. Monson, PhD  Professor of Psychology at Toronto Metropolitan University  416-979-5000 Ext. 556209  Email: [candice.monson@torontomu.ca](mailto:candice.monson@torontomu.ca) |
| **Co-Investigators:** | Dr. Perry Menzies  St. Michael's Hospital and the University of Toronto  416-864-3085  [perry.menzies@unityhealth.to](mailto:perry.menzies@unityhealth.to)  Dr. Rakesh Jetly, MD FRCPC  Associate Professor of Psychiatry at Dalhousie University and University of Ottawa  613-945-6817  [rakesh@drjetly.com](mailto:rakesh@drjetly.com)  Dr. Wendy Lou PhD  Professor of Biostatistics, Dalla Lana School of Public Health, University of Toronto  416-946-7804  [wendy.lou@utoronto.ca](mailto:wendy.lou@utoronto.ca)  Dr. Sridhar (Sri) Krishnan PhD, PEng, FCAE  Professor of Engineering, Signal Analysis Research Group, Toronto Metropolitan University  416-979-5000 Ext. 554931  [krishnan@ryerson.ca](mailto:krishnan@ryerson.ca) |
| **REB Study Number:** | 23-230 |
| **Clinical Trials.Gov #** | Not assigned |
| **Clinical Trial Phase:** | 2 |
| **Planned Clinical Start:** | Upon approval from REB |
| **Planned Clinical End:** | Dependent upon REB approval |
| **Date of Protocol:** | 11-Dec-2023 |
| **Version:** | 5 |

**Table of Contents**

[**List of Abbreviations**](#_heading=h.3dy6vkm)

[**List of Tables**](#_heading=h.tyjcwt)

[**1. Study Summary**](#_heading=h.1t3h5sf)

[**2. Introduction**](#_heading=h.2jxsxqh)

[**2.1. Background and Rationale**](#_heading=h.z337ya)

[**2.2. Pharmacokinetics**](#_heading=h.3j2qqm3)

[**2.3. Preclinical Pharmacology**](#_heading=h.1y810tw)

[**2.4. Clinical Adverse Event Profile**](#_heading=h.4i7ojhp)

[**2.5. Use During Pregnancy and Lactation**](#_heading=h.2xcytpi)

[**2.6. Study Objectives and Hypotheses**](#_heading=h.1ci93xb)

[2.6.1. Primary](#_heading=h.3whwml4)

[2.6.2. Secondary](#_heading=h.2bn6wsx)

[2.6.3. Exploratory](#_heading=h.3as4poj)

[2.6.3.1.Personal Digital Phenotype Profile Definition](#_heading=h.1pxezwc)

[2.6.4. Safety](#_heading=h.49x2ik5)

[**3. Methods**](#_heading=h.2p2csry)

[**3.1. Trial Design**](#_heading=h.147n2zr)

[**3.2. Population**](#_heading=h.32hioqz)

[3.2.1. Number of Participants](#_heading=h.1hmsyys)

[3.2.2. Eligibility Criteria](#_heading=h.41mghml)

[3.2.3. Inclusion Criteria](#_heading=h.2grqrue)

[3.2.4. Exclusion Criteria](#_heading=h.vx1227)

[3.2.5. Withdrawal Criteria](#_heading=h.3fwokq0)

[3.2.6. Deviation from Inclusion/Exclusion Criteria](#_heading=h.1v1yuxt)

[**3.3. Participant Screening**](#_heading=h.4f1mdlm)

[**3.4. Recruitment**](#_heading=h.2u6wntf)

[**3.5. Intervention**](#_heading=h.19c6y18)

[**3.6. Cognitive Processing Therapy**](#_heading=h.28h4qwu)

[**3.7. Oura Ring Wearable Device**](#_heading=h.nmf14n)

[**3.8. Duration of Treatment Period**](#_heading=h.37m2jsg)

[**3.9. Concomitant Medication**](#_heading=h.1mrcu09)

[**3.10. Outcome**](#_heading=h.46r0co2)

[3.10.1. Primary Outcome Measures](#_heading=h.2lwamvv)

[3.10.2. Secondary Outcome Measures](#_heading=h.111kx3o)

### [3.10.3. Exploratory Outcomes](#_heading=h.30j0zll) [Measures](#_heading=h.111kx3o)

[3.10.4. Safety Outcome Measures](#_heading=h.206ipza)

[**3.11. Quality Assurance**](#_heading=h.4k668n3)

[**3.12. Participant Timeline**](#_heading=h.2zbgiuw)

[**3.13. Trial Procedures and Evaluations**](#_heading=h.1egqt2p)

[**3.13.1. Pre-Study Screening**](#_heading=h.3ygebqi)

[**3.13.2. Screening**](#_heading=h.2dlolyb)

[**3.13.3. Eligibility confirmation**](#_heading=h.sqyw64)

[**3.13.4. Pre-baseline wearable monitoring (Oura)**](#_heading=h.3cqmetx)

[**3.13.5. Baseline**](#_heading=h.4bvk7pj)

[**3.13.6. Day 1, 2**](#_heading=h.2r0uhxc)

[**3.13.7. Day 3 (Dosing Day)**](#_heading=h.1664s55)

[**3.13.8. Day 4-7**](#_heading=h.3q5sasy)

[**3.13.9. Follow-up Assessments (Week 5-13)**](#_heading=h.25b2l0r)

**4**[**. Safety**](#_heading=h.kgcv8k)

**4**[**.1. Adverse Events**](#_heading=h.34g0dwd)

4[.1.1. Assessment of Intensity](#_heading=h.1jlao46)

4[.1.2. Assessment of Causality](#_heading=h.43ky6rz)

###### [4.1.3. Action Taken Regarding Investigational Product](https://docs.google.com/document/d/1Whj5CTpaL6qeVyV4FrTQ440LQkFKglaf/edit#heading=h.3hv69ve)

[4.1.4. Adverse Event Outcome](https://docs.google.com/document/d/1Whj5CTpaL6qeVyV4FrTQ440LQkFKglaf/edit#heading=h.1x0gk37)

4[.1.5. Severity versus Seriousness](#_heading=h.2iq8gzs)

4[.1.6. Recording of Adverse Events](#_heading=h.xvir7l)

4[.1.7. Overdose](#_heading=h.3hv69ve)

4[.1.8. Adverse Events of Special Interest](#_heading=h.1x0gk37)

4[.1.9. Adverse Event Follow-up](#_heading=h.4h042r0)

4[.1.10. Serious Adverse Events](#_heading=h.2w5ecyt)

4[.1.11. Reporting of Serious Adverse Events](#_heading=h.1baon6m)

**4**[**.2. Permissible Medications**](#_heading=h.3vac5uf)

**4**[**.3. Prohibited Medications**](#_heading=h.2afmg28)

**4**[**.4. Rescue Medications**](#_heading=h.pkwqa1)

**4**[**.5. ​​Women of Childbearing Potential and Acceptable Contraceptive Methods**](#_heading=h.39kk8xu)

**4**[**.6. Exposure in Utero During Clinical Studies**](#_heading=h.1opuj5n)

**4**[**.7. Auditing**](#_heading=h.48pi1tg)

**5.** [**Investigational Product Management**](#_heading=h.2nusc19)

**5**[**.1. Formulation**](#_heading=h.1302m92)

**5**[**.2. Storage**](#_heading=h.3mzq4wv)

[**6. Ethics and Responsibilities**](#_heading=h.2250f4o)

[**6.1. Good Clinical Practice**](#_heading=h.haapch)

[**6.2. Data and Safety Monitoring**](#_heading=h.319y80a)

[**6.3. Institutional Review Board/Independent Ethics Committee**](#_heading=h.1gf8i83)

[**6.4. Informed Consent**](#_heading=h.40ew0vw)

**6**[**.5. Protocol Deviations**](#_heading=h.2fk6b3p)

[**6.6. Protocol Amendments**](#_heading=h.upglbi)

[**6.7. Records Management**](#_heading=h.3ep43zb)

[**6.8. Source Documentation**](#_heading=h.1tuee74)

[**6.9. Participant Protection**](#_heading=h.4du1wux)

[**6.10. Confidentiality**](#_heading=h.2szc72q)

[**6.11. Study Discontinuation**](#_heading=h.184mhaj)

[**6.12. Dissemination Policy**](#_heading=h.3s49zyc)

[6.12.1. Trial Results](#_heading=h.279ka65)

[6.12.2. Authorship](#_heading=h.meukdy)

[**6.13. Declaration of Interests**](#_heading=h.36ei31r)

[**7. Statistics**](#_heading=h.1ljsd9k)

[**7.1. General Procedures and Statistical Methods**](#_heading=h.45jfvxd)

[**7.2. Sample Size**](#_heading=h.zu0gcz)

## [**8. Personal Digital Phenotype Analysis and Generation**](#_heading=h.1fob9te)

## [**9. Interpretation of the Personal Digital Phenotype**](#_heading=h.3znysh7)

**10**[**. Study Administration**](#_heading=h.2y3w247)

**10**[**.1 Key Contacts**](#_heading=h.1d96cc0)

[**10.2. Funding**](#_heading=h.3x8tuzt)

[**10.3. Roles and Responsibilities**](#_heading=h.2ce457m)

[10.3.1. Sponsor and Funder](#_heading=h.rjefff)

[10.3.2. Trial Committee](#_heading=h.3bj1y38)

######

###### **List of Tables**

**Table 1**. Description of the Oura Ring wearable device and data collection.

**Table 2**. Schedule of Assessment.

**Table 3.** User Experience Questionnaire.

**Table 4.** Prohibited Medications (Not an exhaustive list).

**List of Figures**

**Figure 1.** Study design with timepoints.

**Figure 2.** Summarizes the study aims and presents the outcome measures for each aim.

**Figure 3.** Block diagram representation of the proposed SID pipeline.

######

###### **List of Abbreviations**

**Abbreviation Definition**

ADHD Attention Deficit Hyperactivity Disorder

AE(s) Adverse Event(s)

AESI(s) Adverse Event of Special Interest(s)

AHRC Applied Health Research Centre

AMPA Amino-3-hydroxy-5-methyl-4-isoxazole propionic acid

APEQ Acceptance/Avoidance-Promoting Experiences

Questionnaire

API Application Programming Interface

ASC Altered States of Consciousness Rating Scale

BEAQ Brief Experiential Avoidance Questionnaire

CAPS-5 Clinician-Administered Posttraumatic Stress Disorder Scale for Diagnostic and Statistical Manual for Mental Disorders, Fifth Edition

CI Confidence Interval

CPT Cognitive Processing Therapy

CRF Case Report Form

CSSRS Columbia Suicide Severity Rating Scale

CTQ-SF Childhood Trauma Questionnaire-Short Form

DERS-SF Difficulties in Emotion Regulation Scale-Short Form

DES-II Dissociative Experiences Scale II

DPP Digital Phenotype Profile

DS-II Demoralization Scale-II

DSM-5 Diagnostic and Statistical Manual for Mental Disorders, Fifth Edition

EBI Emotional Breakthrough Inventory

eCRF Electronic Case Report Form

GABA Gamma-aminobutyric Acid

GAD-7 Generalized Anxiety Disorder Scale, 7-item

GCP Good Clinical Practice

HT Hydroxytryptamine

ICF Informed Consent Form

ICG-R Inventory of Complicated Grief-Revised

IPF Inventory of Psychosocial Functioning

IRB Institutional Review Board

MDMA 3,4-methylenedioxymethamphetamine

MINI Mini International Neuropsychiatric Interview

MPFI-24 Multidimensional Psychological Flexibility Inventory

NMDA N-methyl-D-aspartate

PCL-5 Posttraumatic Stress Disorder Checklist-5 (PCL-5)

pDPP Personalized Digital Phenotype Profile

PHQ-9 Patient Health Questionnaire-9

PID Participant Identification

PII Personal Identifiable Information

PIQ Psychological Insight Questionnaire

PMBS Posttraumatic Maladaptive Beliefs Scale

PSQI Pittsburgh Sleep Quality Index

PTSD Posttraumatic Stress Disorder

QRI Quality of Relationships Inventory

REDCap Research Electronic Data Capture

REB Research Ethics Board

RPCA Robust Principal Component Analysis

SAE(s) Serious Adverse Event(s)

SCS-SF Self-Compassion Scale-Short Form

SD Standard Deviation

SDS Sheehan Disability Scale

SID Statistics, Information Theory, Data-driven

SNRI Serotonin Norepinephrine Reuptake Inhibitor

SSRI Selective Serotonin Reuptake Inhibitor

TEAE(s) Treatment Emergent Adverse Event(s)

TRD Treatment Resistant Depression

WAI-SF Working Alliance Inventory-Short Form

WHO-5 World Health Organization Well-Being Index, 5-item

# **Study Summary**

| Name of Sponsor-Investigator:  Dr. Venkat Bhat, St. Michael’s Hospital | |
| --- | --- |
| Name of Investigational Product:  Psilocybin | Protocol Identification Code:  23-230 |

**ClinicalTrials.gov Number**: Not yet assigned

**Clinical Phase**: 2

| Title | Psilocybin-Assisted Massed Cognitive Processing Therapy for Chronic Posttraumatic Stress Disorder: An Open-label Trial |
| --- | --- |
| Sources of monetary or material support | Internal sources of funding |
| Brief title | Psilocybin-assisted CPT for PTSD |
| Indication: | Adults with Chronic Posttraumatic Stress Disorder (PTSD) |
| Condition(s) or focus of study | Posttraumatic Stress Disorder |
| Number of participants | 15 |
| Primary outcome | To determine the feasibility and tolerability of psilocybin-assisted cognitive processing therapy (CPT) in adults with chronic PTSD (i.e., recruitment rate, withdrawals, data completion, adherence, and number and nature of adverse events). |
| Secondary outcomes | 1. To examine the effect of psilocybin-assisted CPT on clinician-rated PTSD symptoms, self-reported treatment outcomes (e.g., depression and anxiety severity, psychological functioning, sleep quality) and putative mechanisms of change. |
| Exploratory outcomes | 1. To evaluate the effect of psilocybin-assisted CPT on digital physiological passive data collected through the use of a wearable device (Oura Ring). 2. To create a personal digital phenotype profile (pDPP) based on the wearable’s and clinical assessments’ data. |
| Safety outcome | 1. To evaluate the safety of psilocybin-assisted CPT in adults with chronic PTSD. |
| Study design | Study type: Open-label  Allocation: N/A  Intervention model: Single arm  Primary outcome: Feasibility  Phase: 2 |
| Masking | N/A |
| Date of enrollment | Start date: Upon REB Approval  End date: Dependent on REB Approval |
| Eligibility criteria | Please see full inclusion and exclusion criteria in section 3.2.2.  Inclusion Criteria:   1. Meet Diagnostic and Statistical Manual-5th edition (DSM-5) criteria for current PTSD with a duration of 6 months or longer; 2. Have a Clinician-Administered PTSD Scale for Diagnostic and Statistical Manual-5th edition (DSM-5) (CAPS-5) score of 50 or higher, indicating moderate to severe PTSD symptoms; 3. Adults between the ages of 18 and 65 (inclusive) who are capable of giving informed consent; 4. Willing to stop prohibited medications; 5. Able to understand and comply with the requirements of the study, as judged by the investigator(s); 6. Agree to video and audio recording of all study visits including therapy sessions and clinical assessments; 7. Ownership of a smartphone.   Exclusion Criteria:   1. Have a history of or a current primary diagnosis of psychotic disorder, schizophrenia, delusional disorder, borderline personality disorder, schizoaffective disorder, bipolar disorder or, dissociative identity disorder; 2. Unable to accurately and completely communicate study information, answer questions about the study, and obtain consent; 3. Have evidence or history of coronary artery disease or cerebral or peripheral vascular disease, hepatic disease with abnormal liver enzymes, or any other medical disorder judged by the investigator to significantly increase the risk of psilocybin administration; 4. First degree family history of bipolar I disorder, schizophrenia or any psychotic disorders, including bipolar disorder with psychotic features; 5. Lifetime history of substance-induced psychosis; 6. Lifetime history of substance use disorder with a hallucinogen. |
| Test products, dose, and mode of administration: | Intervention: 1 dose oral psilocybin 25 mg (5* 5 mg capsules).  Participants will receive 1 week of massed CPT including 12 CPT sessions and 1 preparatory and 1 integration sessions. |
| Follow-up: | Participants will be followed-up weekly from week 1 until 12 weeks post-treatment (weeks 2-13). |
|  |  |
|  |  |

## **2. Introduction**

### **2.1. Background and Rationale**

Posttraumatic stress disorder (PTSD) is a severe psychiatric disorder that may develop as a result of a severely stressful experience, such as interpersonal violence, combat, life-threatening accident, or natural disaster [(1)](https://www.zotero.org/google-docs/?yHsXtt). PTSD is associated with significant distress, functional impairment, health burden, and economic cost [(2)](https://www.zotero.org/google-docs/?y1VX73). The prevalence of PTSD is about 3.9% in the general population [(1,2)](https://www.zotero.org/google-docs/?ZaUF7w). In individuals who have experienced trauma, the prevalence rate is 5.6% [(2)](https://www.zotero.org/google-docs/?Gg8u0S). Women have a lifetime PTSD prevalence rate of 9.7%, compared to 3.6% in men [(3)](https://www.zotero.org/google-docs/?mjtpze). Sexual violence is the main cause of PTSD (33%), with 94% of rape victims exhibiting PTSD symptoms within two weeks after their traumatic experience [(4)](https://www.zotero.org/google-docs/?f3cdgn). PTSD symptoms include persistent intrusive recollections, avoidance of trauma-related stimuli, negative changes in cognitions and mood, and hyperarousal, as well as distressing and intrusive memories and nightmares of the trauma, irritability, hypervigilance, difficulty sleeping, poor concentration, and emotional withdrawal [(2,5)](https://www.zotero.org/google-docs/?0Hq147). PTSD is classified into two forms based on its duration: acute PTSD and chronic PTSD. Acute PTSD occurs when symptoms last less than three months; chronic PTSD occurs when symptoms last more than three months [(6,7)](https://www.zotero.org/google-docs/?xOMkVK).

There are several medications and evidence-based psychotherapies available to treat PTSD. Many patients, however, do not respond to these therapies, which puts them at risk for a chronic condition and poor long-term prognosis [(2,8)](https://www.zotero.org/google-docs/?GW9JOe). While many patients with PTSD achieve remission with or without treatment, a significant number, approximately 50%, struggle with recurrent or chronic PTSD [(9)](https://www.zotero.org/google-docs/?S1Nkus). This is comparable with the estimated 40–60% of patients who do not respond adequately to standard pharmacotherapies [(10)](https://www.zotero.org/google-docs/?EytMQe). Therefore, it is essential to investigate novel approaches as well as novel chemical compounds for the treatment of PTSD.

Although there are several evidence-based psychotherapeutic treatments for PTSD (e.g., prolonged exposure, cognitive processing therapy [CPT]), drop-out rates are relatively high, not all individuals respond to treatment, impairment persists even after remission of the diagnosis, and the onset of treatment effects is delayed [(1,11,12)](https://www.zotero.org/google-docs/?Evsxy2). To fill these gaps, massed trauma-focused interventions that provide daily treatment over the course of 1-3 weeks have been developed and show promising outcomes, including large effect sizes and high completion rates [(13–16)](https://www.zotero.org/google-docs/?g75eRb). Nonetheless, many do not respond to this delivery strategy and continue to meet criteria for PTSD [(17)](https://www.zotero.org/google-docs/?ODiOxw) and the majority of individuals (80-87%) exhibit residual PTSD and depressive symptoms post-treatment [(18)](https://www.zotero.org/google-docs/?Fyds27). Accordingly, there is a need for novel adaptations of massed PTSD treatment that may help accelerate mechanisms of change and optimize treatment outcomes [(19)](https://www.zotero.org/google-docs/?aITWfR).

Psilocybin is a 5-hydroxytryptaminergic (serotonergic) psychedelic and a non-selective serotonin 2A receptor (5-HT_2AR_) agonist that was first synthesized by Albert Hofmann in 1958 after being originally isolated from psilocybe mushrooms by him in 1957 [(20)](https://www.zotero.org/google-docs/?mpOL4o). Psilocybin was used in psychiatric research from the early to mid-1960s until it was designated a schedule 1 drug in the United States (US) in 1970 [(21,22)](https://www.zotero.org/google-docs/?5qnGXh). Research on the effects of psilocybin resumed in the mid-1990s, and it is currently the preferred compound for use in clinical research of 5-hydroxytryptaminergic psychedelics because it has a shorter duration of action and suffers from less notoriety and stigma than other similar drugs [(23,24)](https://www.zotero.org/google-docs/?q0UCMG). Extant literature investigating the use of psychedelic drugs such as psilocybin focuses on the delivery of these drugs under controlled and optimal conditions in order to produce therapeutic effects. These studies hope to harness the psychospiritual experiences that may be induced by drugs such as psilocybin and direct them into therapeutic experiences for individuals with disorders that may be difficult to treat [(25–28)](https://www.zotero.org/google-docs/?9uyuu9).

Psilocybin has a range of effects on emotion, perception, and cognition with transdiagnostic potential due to its rapid action (i.e., within one day of dosing), large effect sizes, and high response and remission rates across a variety of psychiatric disorders [(29–36)](https://www.zotero.org/google-docs/?LaiTdZ). Preliminary lines of research suggest that psilocybin may be a potentially effective treatment for PTSD [(37,38)](https://www.zotero.org/google-docs/?drepQY).

Anderson et al. (2020) conducted an open-label, pilot study using psilocybin-assisted group therapy in 18 individuals with long-term AIDS-related demoralization [(39)](https://www.zotero.org/google-docs/?lVMrjP). There was no control group. Over seven weeks, participants underwent three hours of individual psychotherapy, 12–15 hours of group psychotherapy, and one eight-hour individual psilocybin administration session (oral dose of psilocybin: 0.30-0.36 mg/kg). Feasibility of psilocybin was evaluated by recruitment and retention rates of enrolled participants. The primary outcome measure was changes in the Demoralization Scale-II (DS-II) from baseline to the 3-month follow-up. Patients were monitored for adverse events (AEs) during the dosing sessions and at subsequent clinic and remote follow-ups. The AEs noted were hypertension, anxiety/anxiety exacerbation, nausea, headache, paranoia/ideas of reference, motor agitation/restlessness, unsteady gait/ataxia, tachycardia, thought disorder, urinary incontinence, and visual changes. The results of this study indicated a significant change in demoralization from baseline to the 3-month follow-up (mean difference -5.78 [standard deviation (SD): 6.01], 90% confidence interval (CI): 0.21-0.60). Moreover, PTSD related symptoms (assessed using PTSD Checklist-5 (PCL-5)) and pathological grief (measured using the Inventory of Complicated Grief-Revised (ICG-R)) significantly improved following psilocybin administration [(39)](https://www.zotero.org/google-docs/?KHMsLX). Additionally, the mechanisms of change underlying psilocybin therapy, including reductions in avoidance [(40,41)](https://www.zotero.org/google-docs/?S5GqtA), fear extinction in mice [(42)](https://www.zotero.org/google-docs/?sSea7e), and decreased amygdala activity [(43–45)](https://www.zotero.org/google-docs/?yYw76H) show strong overlap with those essential to efficacious PTSD treatment. Considering the results of this study and psilocybin’s mechanism of action, psilocybin-assisted therapy may be a beneficial treatment for patients with PTSD.

Research on other psychedelic drugs is being examined in patients with PTSD [(46)](https://www.zotero.org/google-docs/?2BINaL). 3,4-methylenedioxymethamphetamine (MDMA) acts as both a stimulant and psychedelic and has been used in PTSD [(47,48)](https://www.zotero.org/google-docs/?IL7luV). Michell et al. (2021) conducted a randomized, double-blind, placebo-controlled phase 3 study to investigate the effect of MDMA-assisted psychotherapy in patients with severe PTSD [(48)](https://www.zotero.org/google-docs/?fQcaRi). Ninety individuals were randomly assigned to receive MDMA or placebo, along with manualized treatment that included three initial sessions of preparation therapy and nine sessions of integrative therapy. The Clinician-Administered PTSD Scale for Diagnostic and Statistical Manual of Mental Disorders, Fifth Edition (DSM-5) (CAPS-5) was used to measure PTSD symptoms as the primary outcome, and the Sheehan Disability Scale (SDS) was used to measure functional impairment two months following the final experimental session. Suicidality and AEs were monitored throughout the study. The Clinician-Administered PTSD Scale for DSM-5 (CAPS-5) score was found to be significantly and persistently reduced in the MDMA group compared to the placebo group (p< 0.001), and the Sheehan Disability Scale (SDS) total score was significantly lower in the MDMA group compared to the placebo group (p = 0.012) [(48)](https://www.zotero.org/google-docs/?N0HSlf). Mithoefer et al. (2018) conducted a randomized, double-blind phase 2 clinical trial in 26 veterans with chronic PTSD [(49)](https://www.zotero.org/google-docs/?grRt5C). Participants were randomly assigned to three different dose groups of MDMA (30 mg, 75 mg, or 125 mg) plus psychotherapy. The treatment course consisted of 18 hours of non-drug psychotherapy and 16-24 hours (2-3 sessions) of MDMA-assisted psychotherapy. The primary outcome of this study was changes in Clinician-Administered PTSD Scale for DSM-5 (CAPS-5) from baseline to 1 month after the second dosing session. Secondary outcomes were changes in depressive symptoms, sleep quality, perceived growth following trauma, personality factors and general psychological function. Seven participants received 30 mg of MDMA and seven and twelve received 75 mg and 125 mg MDMA, respectively. The average change in Clinician-Administered PTSD Scale for DSM-5 (CAPS-5) total score from baseline to 1 month following the second blinded experimental session of MDMA plus psychotherapy was -11.4, -58.3, and -44.3 for groups that received 30 mg, 75 mg, and 125 mg, respectively. The intensity of the PTSD symptoms decreased considerably in the 75 mg (p< 0.001) and 125 mg (p< 0.001) MDMA groups compared to the 30 mg MDMA group. One month after the second blinded experimental session, depressive symptoms in the 125 mg group were significantly reduced compared to the 30 mg group (mean change in Beck Depression Inventory-II score (BDI-II) -24.6 vs. -4.6) (p< 0.001). Similarly, depressive symptoms in the 75 mg group were lower than those in the 30 mg group, although this result was not statistically significant t (-15.4 vs. -4.6, p = 0.052). In terms of mean change in Pittsburgh Sleep Quality Index (PSQI) scores, the 75 mg group improved the most, followed by the 125 mg and 30 mg groups. MDMA was generally well tolerated, with the most frequently reported AEs including anxiety, headache, fatigue, and muscle tension [(49)](https://www.zotero.org/google-docs/?EEiLvf).

Taken together, preliminary studies support the safety and efficacy of psilocybin for PTSD. Psilocybin-assisted CPT for PTSD may be a cost-effective and rapid treatment that optimizes treatment for individuals with PTSD. Therefore, we plan to conduct an open-label pilot trial to assess the feasibility, tolerability, efficacy and safety of psilocybin-assisted CPT in patients with chronic PTSD.

#### **2.2. Pharmacokinetics**

Psilocybin is metabolized (dephosphorylated) in the liver and primarily transformed into the active hydroxy metabolite, psilocin, after being taken orally. As a result, psilocybin is primarily a prodrug, while psilocin is the pharmacologically active agent in systemic circulation [(50)](https://www.zotero.org/google-docs/?SEqND8). Oral psilocybin has a 50% bioavailability, and psilocin is detected in plasma within 15-20 minutes following parent compound administration [(51)](https://www.zotero.org/google-docs/?aqV9DQ). The average blood concentration of the active metabolite psilocin following oral administration of psilocybin (0.224 mg/kg) was determined to be 8.2±2.8 ng/mL after 105±37 minutes, resulting in an estimated psilocybin dose-normalized bioavailability of 52.7±20% [(50,51)](https://www.zotero.org/google-docs/?sDI6ZY).

The half-life of psilocin after oral administration of psilocybin was determined to be around 3±1.1 hours, and it is detectable for up to 24 hours after administration [(50,51)](https://www.zotero.org/google-docs/?Y9zTAX). Hasler et al. (2002) and Lindenblatt et al. (1998) found comparable but not identical results, with peak psilocin levels appearing between 80 and 105 minutes and psilocin half-life varying between 2.25 hours for 0.2 mg/kg and 2.7 hours for 0.22 mg/kg [(52,53)](https://www.zotero.org/google-docs/?xREDsg). The peak psilocin concentration was reached more gradually in some subjects than in others, suggesting that metabolism rates might vary between individuals [(50)](https://www.zotero.org/google-docs/?K1cp5y).

Eighty percent of the psilocin detected in plasma is conjugated. Psilocin (90-97%) and psilocybin (3-10%) are both detectable in human urine, both unmodified (only 3-10%) and particularly conjugated with glucuronic acid [(53)](https://www.zotero.org/google-docs/?ZI59nx). The majority of the psilocin recovered in urine is excreted after 3 hours of oral administration and is completely eliminated within 24 hours [(53)](https://www.zotero.org/google-docs/?WyOjJh). Psilocybin (as psilocin) pharmacokinetics are linear at a dose range of 0.3-0.6 mg/kg [(51–53)](https://www.zotero.org/google-docs/?nApt6A).

##### **2.3. Preclinical Pharmacology**

Psilocybin and its active metabolite psilocin directly affect a number of 5-hydroxytryptamine (5-HT) receptor subtypes without directly affecting other neurotransmitter systems. Psilocybin’s potential for use in treatment resistant depression (TRD) treatment lies in its ability to modulate 5-HT receptors. As a 5-HT_2AR_ agonist, the downregulation of 5-HT_2A_ receptors produces antidepressant and anxiolytic effects, which are evident in more commonly used antidepressants and atypical antipsychotics [(26)](https://www.zotero.org/google-docs/?ng5aE8). When ingested, psilocybin is metabolized by intestinal alkaline phosphatases and esterases, which rapidly dephosphorylate the alkaloid into psilocin [(31,32)](https://www.zotero.org/google-docs/?UXUVX3). The putative target of psilocin and psilocybin is the agonism of the 5HT_2_ receptor class; however, studies have confirmed that there is also affinity for 5HT_1_, 5HT_4_, 5HT_5_, 5HT_6_, 5HT_7_, Dopamine 1 and 3 receptors [(54)](https://www.zotero.org/google-docs/?7yrNNH). Psilocybin, a naturally occurring alkaloid, has been classified, along with its active metabolite psilocin, as a tryptamine [(55)](https://www.zotero.org/google-docs/?wDuML4).

The PTSD pathophysiology involves alterations in neurotransmitters such as gamma-aminobutyric acid (GABA), glutamate, serotonin, neuropeptide Y, and other endogenous opioids and neurohormonal functioning [(2,56)](https://www.zotero.org/google-docs/?rJ1Uq7). It has been proposed that 5-HT_2AR_ agonism raises extracellular glutamatergic concentrations in the prefrontal cortex [(57)](https://www.zotero.org/google-docs/?5L6qfk). Following glutamatergic modulation of amino-3-hydroxy-5-methyl-4-isoxazole propionic acid (AMPA) and N-methyl-D-aspartate (NMDA) receptors on cortical pyramidal cells, neurotrophin (i.e., brain derived neurotrophic factor) expression is upregulated [(57)](https://www.zotero.org/google-docs/?BsAb9f).

###### **2.4. Clinical Adverse Event Profile**

Psilocybin is generally tolerated well. Transient anxiety during psilocybin onset (20-100%), transient confusion or thought disorder (17-75%), mild transient nausea (15-33%), transient headache (33-75%), and transient elevated blood pressure (2-34%) or heart rate (6-8%) are the most common AEs with 20 mg to 30 mg of oral psilocybin [(58,59)](https://www.zotero.org/google-docs/?bXTxrX).

Onset of noticeable psychoactive effects typically occurs within one hour, peaks at about two hours after a dose, and loss occurs typically around six hours after the dose [(60)](https://www.zotero.org/google-docs/?icuc6I). Psilocybin reliably induces profound changes in sensory perception, emotion, thought, and sense of self, characterized by marked alterations in all mental functions, including perception, mood, volition, cognition and self-experience [(61,62)](https://www.zotero.org/google-docs/?aPKYWD). These profound changes are often referred to as mystical-type experiences which are defined as having profound and lasting personal value and spiritual significance toward which participants attributed prolonged beneficial improvements in attitudes, mood and behavior [(63,64)](https://www.zotero.org/google-docs/?Mf8JuE). Mystical-type experiences occurring during psilocybin treatment have often been observed to predict later effects on behavior and emotions [(34,63,64)](https://www.zotero.org/google-docs/?pboda4). However, in a study by Gukasyan et al. (2022) mystical experience after psilocybin dosing sessions only predicted self-reported increases in well-being, but did not predict improvement in depressive symptoms [(65)](https://www.zotero.org/google-docs/?UXvS4F).

Taken together, the safety of psilocybin should be considered in terms of benefit and risk. Within the context of psilocybin administration in a controlled setting, a participant may report visual or auditory disturbances, feelings of unreality, altered sense of time, and other changes in mood or affect. These effects are expected, and may be a necessary component of therapeutic response [(32)](https://www.zotero.org/google-docs/?o0HPa6). Investigators must follow regulatory guidance under 21 CFR 312.32(a) for AE reporting which addresses untoward medical occurrences associated with the use of a drug in humans, whether or not considered drug related. An AE can be any unfavorable and unintended sign (eg, an abnormal laboratory finding), symptom, or disease temporally associated with the use of a drug, without any judgment about causality. However, because self-reports of intrapsychic events associated with psilocybin usage may be neither unfavorable nor unintended, an investigator may not regard the self-reports as AEs but rather as an essential product attribute for therapeutic response. The reporting of psilocybin associated observations as AEs should be informed by this context.

###### **2.5. Use During Pregnancy and Lactation**

There have been no human case reports or studies involving the effects of psilocybin on pregnancy. It is recommended that women who are pregnant avoid using psilocybin. Women of childbearing potential who have a negative pregnancy test at screening will undergo repeated pregnancy testing prior to treatment administration, and only if the results are negative psilocybin will be administered. Any pregnancy occurring after study enrollment should be followed until an outcome is known (i.e., spontaneous miscarriage, elective termination, normal birth). All live births must be followed for a minimum of 30 days or to the first well-baby visit. Non-clinical and clinical data describing the effects of oral psilocybin on lactation, sperm, and teratogenicity are not available.

###### **2.6. Study Objectives and Hypotheses**

###### **2.6.1. Primary**

The primary objective of this clinical trial is to evaluate the feasibility and tolerability of psilocybin-assisted CPT in adults with chronic PTSD. Feasibility and tolerability will be determined using recruitment rates, withdrawal, data completion, adherence, number and nature of AEs. **H1**: It is hypothesized that psilocybin and study participation will be generally well tolerated with a 10% study dropout rate.

###### **2.6.2. Secondary**

The secondary objectives are to assess the efficacy of psilocybin-assisted CPT at point-of-care. Reduction in clinician-rated PTSD severity, self-reported treatment outcomes (e.g., PTSD severity, depression and anxiety severity, psychological function, sleep quality) and putative mechanisms of change will be assessed. **H2:** It is hypothesized that there will be a significant decrease in clinician-rated and self-reported PTSD severity. The following will be used as secondary outcomes:

- Assess changes in PTSD symptoms via Clinician-Administered PTSD Scale for DSM-5 (CAPS-5)
- Assess changes in self-reported PTSD symptoms as measured with the Posttraumatic Stress Disorder Checklist for the DSM-5 (PCL-5)
- Assess changes in self-reported depression symptoms with the Patient Health Questionnaire-9 (PHQ-9)
- Assess changes in self-reported anxiety symptoms with the Generalized Anxiety Disorder Scale, 7-item (GAD-7)
- Assess changes in self-reported dissociation symptoms with the Dissociative Experiences Scale II (DES-II)
- Assess changes in self-reported sleep quality with the Pittsburgh Sleep Quality Index (PSQI)
- Assess changes in self-compassion, measured with the Self-Compassion Scale short form (SCS-SF)
- Assess changes in experiential avoidance, measured with the Brief Experiential Avoidance Questionnaire (BEAQ)
- Assess changes in relationship satisfaction, measured with the Quality of Relationships Inventory (QRI)
- Assess avoidance, therapeutic alliance, emotion regulation using: Inventory of Psychosocial Functioning (IPF), 24 item Multidimensional Psychological Flexibility Inventory (MPFI-24), Difficulties in Emotion Regulation Scale-Short Form (DERS-SF), Posttraumatic Maladaptive Beliefs Scale (PMBS), Working Alliance Inventory-Short Form (WAI-SF), World Health Organization Well-Being Index, 5-item (WHO-5)
- Assess acute experiences following psilocybin session using: Psychological Insight Questionnaire (PIQ), Emotional Breakthrough Inventory (EBI), Altered States of Consciousness Rating Scale (ASC), Acceptance/Avoidance-Promoting Experiences Questionnaire (APEQ)

###### **2.6.3. Exploratory**

1. To evaluate the effect of psilocybin-assisted CPT on digital physiological passive data collected through the use of a wearable device (Oura Ring).
2. To create a personal digital phenotype profile (pDPP) based on the wearable’s and clinical assessments’ data.

###### **2.6.3.1. Personal Digital Phenotype Profile Definition**

We propose the definition of digital phenotype profile (DPP), which calculates an individual’s baseline and continuously monitors for changes in DPP characteristics derived from mathematical models. Digital phenotype profile (DPP) is a representation of a user's physical and behavioral health using the baseline data collected at the beginning of the study as reference. The models are then fine-tuned with the continuous incoming data streams. However, variations of the model representations require further investigation to ensure its accuracy and robustness. This work investigates the statistical aspect of the analysis pipeline to offer a robust DPP representation. Specifically the use of robust principal component analysis (RPCA) to extract sparse representation. The DPP is developed through our Statistical, Information Theory, and Data-driven (SID) pipeline, then is represented through the sparse-rank matrix using RPCA.

Once the DPP representation has been created, we additionally enhance this definition by proposing the term personal digital phenotype profile (pDPP) as a personalized version of the DPP, where we use collected longitudinal data to monitor the changes in physical and behavioral health of an individual participant.

The process of digital phenotyping involves building a mathematical model using the active and passive data collected from each participant. An individual model is built for each person by incorporating their physiological data together with their self-reported affective states collected from questionnaires. Once the personal profile/model has been constructed, physiological data (such as that collected from the Oura Ring) can be used to provide insight into a person's affective state, even without any active data. In the long run, such models could allow for insights into patients’mental well-being in a non-intrusive way that can improve mental health care and responses to stress.

###### **2.6.4. Safety**

The safety objectives of the study are to monitor and assure the safety of participants before, during and after the psilocybin dosing session and during the study period by assessing severity, incidence and frequency of physiological effects, psychological distress, spontaneously reported reactions, AEs, Treatment Emergent AEs (TEAEs), AEs of Special Interest (AESIs), serious AEs (SAEs), medical events, concomitant medication use, and suicidal ideation and behavior.

- Suicidality will be assessed using the Columbia Suicide Severity Rating Scale (C-SSRS) according to the schedule of assessment table.
- Before and after the experimental session vital signs including blood pressure, heart rate, and temperature will be collected.
- Medical events requiring a doctor’s visit, medications for treatment and psychiatric medications will be collected.
- Assess incidence of AEs by severity.
- Assess incidence of TEAEs by severity to analytically determine relationship to the psilocybin.
- Assess incidence of AESIs, defined as AEs specified in the protocol related to cardiac function, suicide risk, and abuse liability.
- Assess incidence of AEs.
- Assess incidence of SAEs.

###### **3. Method**

###### **3.1. Trial Design**

We propose to conduct an open-label trial in which 15 individuals with PTSD receive 1 week of massed CPT combined with a single dose of psilocybin (25 mg) (Figure 1). Participants will meet criteria for a diagnosis of chronic (i.e., at least six months duration) PTSD with a Clinician-Administered PTSD Scale for DSM-5 (CAPS-5) score of 50 or higher. CPT will be provided remotely twice daily (90 minutes total) over the course of a week. On the third day of the intervention, participants will come in for an in-person (6-8 hour) psilocybin dosing session. Participants will receive a total of 12 CPT sessions, 2 psychotherapy sessions related to psilocybin (preparatory and integration sessions), and one psilocybin dosing session over 7 days. The preparatory session will be completed on day 2 of the treatment week and the integration session will be completed a day after the dosing session (day 4). ​​The primary endpoint of this study will be 1 week after the end of the treatment week (week 2). All study visits including therapy sessions and psilocybin session as well as clinical assessments, will be audio and video recorded, with all recordings preserved for research, training, adherence monitoring and quality assurance purposes. We will measure clinician-assessed PTSD symptoms via Clinician-Administered PTSD Scale for DSM-5 (CAPS-5) global severity scores at baseline, the last treatment day (day 7), and at weeks 5 and 13 (follow-up). Additional outcome measures (i.e., self-reported PTSD symptoms, depression and anxiety severity, psychological function, sleep quality) and measures of putative psychological mechanisms of change (e.g., posttraumatic cognitions, experiential avoidance) will also be measured. Participants will be followed-up weekly from week 2 until week 13 (1 week until 12 weeks post-treatment). Participants will use a commercially available wearable device (Oura Ring) to collect passive data that is relevant to mental health and well-being such as physiological signals, sleep and activity patterns. For more details on the study schedule and administration of scales, please see Table 2.


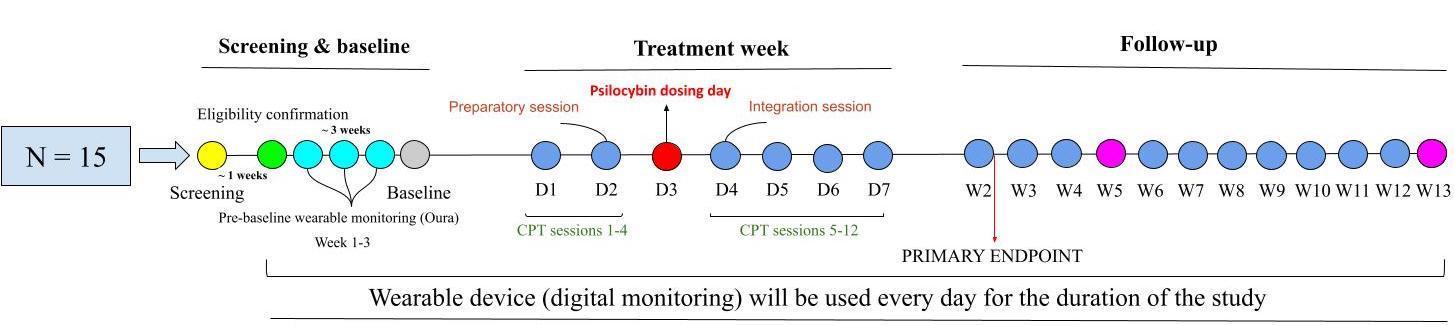


**Figure 1.** Study design with timepoints.

######

###### **3.2. Population**

###### **3.2.1. Number of Participants**

A total of 15 participants are planned to be enrolled in the study.

###### **3.2.2. Eligibility Criteria**

###### **3.2.3. Inclusion Criteria: Individuals eligible to be enrolled into this protocol are participants who:**

1. Meet Diagnostic and Statistical Manual-5th edition (DSM-5) criteria for current PTSD with a duration of 6 months or longer assessed by study psychiatrist;
2. Have a Clinician-Administered PTSD Scale for DSM-5 (CAPS-5) score of 50 or higher, indicating moderate to severe PTSD symptoms;
3. Between the ages 18 and 65, inclusive;
4. Are willing to refrain from taking any psychiatric medications during the study period, with the exception of gabapentin when prescribed for pain control. If the subject is on stimulants for attention-deficit/hyperactivity disorder (ADHD) at baseline, they can continue to use them at the same dose and frequency as long as they discontinue 5 half lives before the psilocybin session and do not restart for 10 days after psilocybin administration. Any psychoactive drugs, including stimulants, will be tapered in an appropriate fashion to avoid withdrawal effects. Medications will only be discontinued after consultation with the study psychiatrist;
5. Agree that, for one week preceding the psilocybin session, they will refrain from:
   1. taking any herbal supplement (except with prior approval of the research team);
   2. taking any nonprescription medications (with the exception of non-steroidal anti-inflammatory drugs or acetaminophen) unless given prior approval from the research team);
   3. taking any prescription medications (with the exception of birth control pills, thyroid hormones or other medications approved by the research team);
6. Refrain from the use of any psychoactive drug, with the exception of caffeine or nicotine, within 24 hours of the psilocybin session;
7. Agree not to use caffeine or nicotine for 2 hours before and 6 hours after the dose of psilocybin;
8. Agree not to use alcohol for 14 hours before and 24 hours after the dose of psilocybin;
9. Agree not to use benzodiazepines, hypnotics, and mood stabilizers for 12 hours before and 12 hours after the dose of psilocybin;
10. Agree not to use steroids for 2 weeks before and 2 weeks after the dose of psilocybin;
11. Agree not to use s-Adenosyl methionine (SAM-e), 5-Hydroxytryptophan (5-HTP), St. John’s Wort for 1 week before and 1 week after the dose of psilocybin;
12. Agree not to use cannabis for 1 week before and 1 week after the dose of psilocybin;
13. Agree not to receive vaccinations for 1 week before and 1 week after the dose of psilocybin;
14. Are willing to be driven home after the psilocybin session, either by a family member, friend, or chaperone and agree not to drive for at least 24 hours after the dosing;
15. Are willing to be contacted via telephone or virtual platform for all necessary contacts;
16. Must provide a contact (relative, spouse, close friend or other caregiver) who is willing and able to be reached by the investigators in the event of a participant becoming suicidal;
17. Must agree to inform the investigators within 48 hours of any medical conditions and procedures;
18. Are proficient in speaking and reading English;
19. Able to understand and comply with the requirements of the study, as judged by the investigator(s);
20. Agree to have all study visits including clinical assessments and therapy sessions including CPT, preparatory and integration sessions audio and video recorded;
21. Agree to not participate in any other interventional clinical trials during the duration of this study;
22. Agree to be monitored by a family member, friend, or chaperone for 24 hours after the dose was administered;
23. Agree to be accessible to the research team 24 hours of the 7 days of the week;
24. Are able to swallow capsules;
25. Ownership of a smartphone.

###### **3.2.4. Exclusion Criteria: Individuals not eligible to be enrolled into this protocol are those who:**

1. Are pregnant or nursing, or are women of child bearing potential who are not practicing an effective means of birth control. Women of childbearing potential (i.e. assigned female at birth, fertile, following menarche and until becoming postmenopausal unless permanently sterile) must have a highly sensitive negative pregnancy test at study entry and prior to psilocybin session, and must agree to use adequate birth control through 10 days after the psilocybin session. Adequate birth control methods include intrauterine device (IUD), injected, implanted, intravaginal, or transdermal hormonal methods, abstinence, oral hormones plus a barrier contraception, vasectomized sole partner, or double barrier contraception. Two forms of contraception are required with any barrier method or oral hormones (e.g., condom plus diaphragm, condom or diaphragm plus spermicide, oral hormonal contraceptives plus spermicide or condom);
2. Have a history of or a current primary diagnosis of psychotic disorder, schizophrenia, delusional disorder, borderline personality disorder, schizoaffective disorder, bipolar disorder or, dissociative identity disorder;
3. Have evidence or history of coronary artery disease or cerebral or peripheral vascular disease, hepatic disease with abnormal liver enzymes, or any other medical disorder judged by the investigator to significantly increase the risk of psilocybin administration;
4. Have hypertension using the standard criteria of the American Heart Association (values of 140/90 or higher assessed on three separate occasions;
5. History of seizure disorder;
6. Uncontrolled insulin-dependent diabetes;
7. Recent stroke, intracranial or subarachnoid hemorrhage (< 1 year from signing of informed consent form [ICF]), recent myocardial infarction (< 1 year from signing of ICF), clinically significant arrhythmia (< 1 year from signing of ICF);
8. Have liver disease with the exception of asymptomatic subjects with Hepatitis C who have previously undergone evaluation and successful treatment;
9. Lifetime history of substance-induced psychosis;
10. Lifetime history of substance use disorder with a hallucinogen;
11. History of alcohol use disorder in the past 3 months;
12. Would present a serious suicide risk, as determined through a psychiatric interview, responses to Columbia Suicide Severity Rating Scale (C-SSRS) (cut-off ⩾3 points) and through the clinical judgment of the investigator, or who, in the judgment of the investigator, are likely to require hospitalization during the course of the study;
13. Would present a serious risk to others as established through clinical interview and contact with treating psychiatrist;
14. Have used psilocybin at least once within 12 months of the psilocybin session;
15. Require ongoing concomitant therapy with a psychiatric drug;
16. Active substance abuse or dependence for any substance save caffeine or nicotine in the past 3 months;
17. Are not able to give adequate informed consent;
18. Positive urine drug screen for illicit drugs or drugs of abuse at screening (any positive urine drug test will be reviewed with participants to determine the pattern of use and eligibility will be determined at the investigator’s discretion);
19. Current enrolment in any investigational drug or device study or participation in such within 30 days of screening;
20. Abnormal and clinically significant results on the physical examination, vital signs, electrocardiogram, or laboratory tests at screening;
21. Any other clinically significant cardiovascular, pulmonary, gastrointestinal, hepatic, renal or any other major concurrent illness or concerns that in the opinion of the investigator, may interfere with the interpretation of the study results or constitute a health risk for the participant if he/she takes part in the study;
22. Have any current problem or a history of substance abuse which, in the opinion of the investigator or medical monitor, might interfere with participation in the protocol;
23. First degree family history of bipolar I disorder, schizophrenia or any psychotic disorders, including bipolar disorder with psychotic features;
24. Unable to accurately and completely communicate study information, answer questions about the study;
25. Have evidence or history of moderate and severe renal disease.

###### **3.2.5. Withdrawal Criteria**

Participants are free to withdraw their consent and/or end their participation in the research study at any time without penalty or loss of benefits or treatment to which they are otherwise entitled. Participants may be withdrawn from the study by the study’s doctor if they feel that it is in their best interest.

Participants may also be discontinued if:

- They fail to adhere to the study’s responsibilities;
- The participant meets any exclusion criteria (either newly developed or not previously recognized);
- They present with any AE or unknown allergic reaction to psilocybin;
- Anything, in the opinion of the investigator, that would place the participant at increased risk or preclude the participant’s full compliance with or completion of the study;
- They are pregnant or of childbearing potential and not willing to use an approved method of contraception during the study.

If a participant experiences an AE they will receive the necessary treatments to alleviate any untoward event and will be monitored by the study physician until it is safe for them to be discharged. Participants will be followed up the next day to monitor the progression of their condition. Data from a participant who has been withdrawn will be collected up to the next day after the withdrawal to ensure that the participant has no delayed complications. If a participant withdraws from the study at any time, the reasons for withdrawal will be collected and documented as part of the feasibility outcome for this study. Data collected up to the point of withdrawal will be used for analysis.

If a subject’s participation in the study is terminated prematurely for any reason, the reason for early discontinuation should be thoroughly documented in the case report form (CRF), and the end-of-study assessments should be completed where possible. A termination CRF page should be completed for every participant who receives a dose of psilocybin.

Reasons for a participant’s premature study discontinuation may be categorized as follows:

- Screen Failure: The participant signed the ICF but was deemed ineligible for the study during the screening process;
- AE: An untoward clinical or laboratory event that, in the medical opinion of the investigator, are grounds for early study termination to protect the best interests of the participant. This includes serious and non serious AEs, regardless of their causal relationship to psilocybin;
- Withdrawal of Consent: The participant withdrew their consent from the study after signing the ICF. Every reasonable effort should be made by study personnel to determine the cause for withdrawal of consent, and the reason should be documented accordingly in the participant's CRF;
- Protocol Violation: The participant’s conduct failed to meet protocol entry criteria, or the participant did not meet study requirements (i.e., non-compliance). The protocol violation necessitated early termination from the study;
- Lost to Follow-Up: The participant stopped attending study visits, and study personnel were unable to contact the participant. The study personnel should make a reasonable attempt to establish contact with the participant if they become lost to follow-up;
- Other: The participant was discontinued for a reason other than those listed above.

###### **3.2.6. Deviation from Inclusion/Exclusion Criteria**

No deviations will be permitted from the inclusion or exclusion criteria.

###### **3.3. Participant Screening**

Participants will be screened at the Interventional Psychiatry Program at St. Michael’s Hospital, Unity Health Toronto. Before screening, participants will sign a print version of the ICF. Then, participants will undergo screening to confirm eligibility. Participants who are not eligible during the screening will be allowed to re-try on a case by case basis.

###### **3.4. Recruitment**

Participants will be recruited from Interventional Psychiatry Program, St. Michael’s Hospital and referred from PTSD Psychotherapy Program lead by Dr Monson at Toronto Metropolitan University. The target recruitment rate is at least 2-3 participants per month for a total of 15 subjects recruited over 7 months. Participants who experience chronic PTSD and are interested in the study will be directed to the study coordinator. The study coordinator will then reach out to the potential participants through a phone or Zoom meeting, where they will be provided with a brief background about the study and the required time commitment before scheduling the screening visit.

###### **3.5. Intervention**

Participants will receive 25 mg (five capsules of 5mg) of psilocybin. The dosing session will be scheduled on day 3 of the treatment week. The study coordinator and the therapist with whom they have been completing CPT sessions will be present on site for the session. After the acute effects of the psilocybin pass, participants will be evaluated for safety and discharged home with a chaperone. The participant should have a light breakfast two hours before receiving psilocybin. Under the direct supervision of the therapist, 25 mg (5 capsules of 5 mg) of psilocybin will be swallowed. The dosing session will take 6-8 hours. Psilocybin effects usually begin 20-30 minutes after administration and peak at 90-120 minutes post-administration. Within 5-6 hours, the effects eventually subside. To minimize distraction and interruption, the participant is instructed to lie down on a couch in a room with low lighting after taking the psilocybin. To encourage inward reflection, the participant will listen to a pre-programmed selection of music during the session. A therapist specialized in providing reassurance and a safe environment will accompany the participant until the effects of the dose have passed. A physician will be available on site in case of an emergency. Moreover, the study coordinator will be at the study center during the psilocybin dosing session to support the therapist if needed. The participant must remain in the room for the whole duration of the session, regardless of intensity. Unless the participant has to communicate discomfort, ask for support from the therapist, or use the toilet, the participant should stay lying down. The therapist is to check-in on how the participant is doing every 1-2 hours following dosing. The participant will be served a light meal and fruit. The therapist will review the psilocybin administration experience with the participant 5 to 6 hours after dosing. When the therapist determines the acute effects of the psilocybin have subsided, which should occur between 6 and 8 hours after taking the dose, the participant will be discharged. The participant will be discharged from the study setting into the care of a responsible person (such as a family, friend, or chaperone) who will accompany the participant home and monitor them for 24 hours after the dose was given. Research coordinator will complete pre dose and post dose assessments. Post dose assessment will be completed after acute effects of psilocybin are resolved. Upon completion of the post-dose assessments, the participant will be assessed for safe discharge under the supervision of a designated chaperone.

###### **3.6. Cognitive Processing Therapy**

Participants will receive a total of 12 CPT sessions, 2 psychotherapy sessions related to psilocybin, and one psilocybin dosing session over 7 days. Therapy sessions will be completed by one therapist. On days 1 and 2, CPT sessions 1-4 and the preparatory session will be delivered virtually. These sessions include psychoeducation about PTSD, reviewing the impact the trauma has had on the individual, identifying maladaptive trauma-related beliefs, and beginning cognitive restructuring. The participant will have one preparatory session with a trained therapist to build rapport, assess patient readiness and prepare the participant for the psilocybin session. The preparatory session will be completed on day 2 and will last approximately 45 minutes. On day 3, the psilocybin dosing session (6-8 hours) will be completed in person. During the psilocybin session, the therapist’s goal should be directed toward reducing prolonged symptoms of anxiety or agitation. The therapist should be directive during the session when they feel it is necessary to ensure participant safety, but will avoid active coaching or voicing of interpretations. On the day after the dosing session (day 4), integration session and CPT sessions 5 and 6 will be completed virtually. These sessions include continued cognitive restructuring, with an emphasis on challenging blame-related beliefs. In an integration session which will last 1 hour, participants will discuss their experience during the psilocybin session with the therapist. CPT sessions 7–12 will be completed virtually twice a day until day 7. These sessions focus on PTSD relevant themes (safety, trust, power/control, esteem, and intimacy).

All therapy sessions will be audio and video recorded for research, training, adherence monitoring and quality assurance purposes. The recordings are necessary for developing the experimental treatment and assessing adherence to the Treatment Manual. If the videos are being used for matters outside of research and training, participants will be notified and contacted to request permission for use. Participants will have control over any presentation of this material beyond viewing by researchers or regulatory agencies.

The study will use encrypted, secure technology to transfer and store recordings, but there is always a risk of a security breach. The sponsor-investigator is committed to taking preventative measures to avoid such an event. In the case of a security breach, the participant will be notified and all efforts will be made to minimize the dissemination of recorded content.

###### **3.7. Oura Ring Wearable Device**

At least three weeks before baseline, participants will use a commercially available wearable device (Oura Ring) (<https://ouraring.com>) to collect passive data that is relevant to mental health and well-being such as physiological signals, sleep and activity patterns. This device is a passive wearable device that can be used without obstructing one’s daily life. The Oura Ring device has its own mobile app for data visualization and self-management.

The Oura Ring has been successfully used in previous research studies to provide information on the behavior of body systems (e.g., autonomic nervous system, sleep/circadian rhythm) in real-time through accurate measures of physiological parameters, sleep and activity [(66–68)](https://www.zotero.org/google-docs/?BuYY1P). In the proposed study, we will use Oura Ring (Generation 3) to continuously collect activity levels, sleep staging, heart rate (HR), heart rate variability (HRV), respiratory rate, body temperature variability (delta), respiratory rate, and oxygen saturation (SpO2). The description of the physiological signals to be collected by Oura Ring is provided below as follows:

1. Sleep information: Oura Ring performs sleep analysis and stores a set of measurement parameters that summarize each period. The ring calculates the sleep period specific parameters within four hours from the period end, but sleep analysis is always triggered when you open the application. These parameters include bedtime, total duration, awake, light, rapid eye movement (REM), and deep sleep durations, and also heart rate, breathing, temperature changes during sleep.
2. Activity information: Activity summary contains daily activity summary values and detailed activity levels. Activity levels are expressed in metabolic-equivalent of task minutes (MET mins). Oura tracks activity based on the movement and presents the activity score based on duration of low, medium, and high activity levels. The movement is also used for step count and to estimate calories.
3. Readiness information: Readiness score is interpreted from sleep, activity, resting heart rate, heart rate variability, recovery index and temperature scores. A Readiness Score above 85% indicates that you're well recovered. A score below 70% usually means that an essential Readiness Contributor, such as body temperature or previous night's sleep, falls outside your normal range, or clearly differs from recommended, science-based values.

Further information on the features to be collected by the Oura Ring are summarized in Table 1. Data collection via the Oura Ring will occur from screening and continue throughout the end of study. Data collection via Oura will also be de-identified. To be more specific, each participant will be assigned a dummy email address and password which will be used to login to their account on the Oura app. This will ensure that participants are de-identified on this third-party platform (Oura). The wearable device privacy policy will be made available for all potential users to read. The following steps will outline the data collection process of the Oura Ring device.

1. The account and device will be set up by a research member with a corresponding unidentified ID. The data will be anonymous and only identifiable by research members.
2. The raw data (e.g., accelerometer) and derived metrics will be collected to the local devices and synced to its respective server. The Oura Ring platform has its own secure server where the data will be stored independently from active data.
3. The research team will have access to the Oura Ring Application Programming Interface (API) to extract data from its respective server. The API will securely connect to the respective servers to extract available information. Raw data will be inaccessible to the research team and securely stored on Oura’s respective servers. The resultant metrics (e.g., sleep score) that are derived from the raw data will be available to the research team for analysis and be extracted using the Oura Application Programming Interface (API). The data will be securely extracted and stored on the Unity Health Toronto servers.

After follow-up is complete, the device will be returned to the study coordinator and will be cleaned and sanitized before being given to another participant.

**Table 1**. Description of the Oura Ring wearable device and data collection.

| **Description** | **Data Transfer** | **Data elements** |
| --- | --- | --- |
| The Oura Ring is a smart ring that is worn on the finger and passively collects physiological parameters and information on sleep and activity levels of an individual. It is considered a smart ring that uses advanced sensor technology to allow for precise, personalized health insights about one’s body. The device measures daily activity, oxygen saturation, blood pressure, and skin temperature. Additionally, the device scores an individual’s readiness, sleep, and activity levels. | Participants will download and log on to the Oura Ring app. The Oura Ring is to be connected via Bluetooth to the participant's smartphone. The Oura Ring will collect raw data locally and send it to the mobile app when synced. Once the Oura Ring app has access, the data will be stored onto its respective servers. For data analysis, only authorized research members will have access to the data collected by Oura Ring through its respective application programming interface (API). | Physiological and activity data:   - Heart rate - Heart Rate Variability (HRV) - Respiration rate - Daily Activity levels - Oxygen Saturation (SpO2) - Body temperature variability (delta) - Readiness score - Calories   Sleep data:   - Sleep stages: deep sleep, REM, light sleep, and awake periods - Sleep score - Nap detection |

###### **3.8. Duration of Treatment Period**

The intervention will involve 1 dosing day and 14 additional psychotherapy sessions spanning 1 week. Follow-up sessions will be completed weekly from week 2 until week 13 (1 week until 12 weeks post-treatment).

###### **3.9. Concomitant Medication**

Participants will be asked to remain on stable doses of concomitant medications. No medication changes, unless approved or requested by research staff, are allowed during the study. If any changes to concomitant psychotropic medications are required per the patient’s standard of care, the participant will be withdrawn from the study. All prescription and non-prescription medications (including over-the-counter medications, vitamins, and herbal supplements) taken by the participant within 30 days prior to the screening period will be assessed and documented. Concomitant medications will be assessed and documented from the time the participant signs the ICF to their final study visit. Any changes, additions, or discontinuations of any medications are to be documented at each study visit, or as needed. Please see permissible and prohibited medications in section 4.2 and 4.3.

###### **3.10. Outcomes**

###### **3.10.1. Primary Outcome Measures**

Feasibility & tolerability will be measured by:

1. Recruitment rate (minimum threshold: 2-3 participants/month)
2. Withdrawal rate (maximum threshold: 20%)
3. Data completion rate (minimum threshold: 80%)
4. Adherence rate (minimum threshold: 80%)
5. Percentage of participants with AEs (maximum threshold: 20%)

###### **3.10.2. Secondary Outcome Measures**

The secondary outcomes measures include: Clinician-Administered PTSD Scale for DSM-5 (CAPS-5), PTSD Checklist-5 (PCL-5), Patient Health Questionnaire-9 (PHQ-9), Generalized Anxiety Disorder Scale, 7-item (GAD-7), Dissociative Experiences Scale II (DES-II), Pittsburgh Sleep Quality Index (PSQI), World Health Organization Well-Being Index, 5-item (WHO-5), Quality of relationships inventory (QRI), Inventory of psychosocial functioning (IPF), Posttraumatic Maladaptive Beliefs Scale (PMBS), Brief Experiential Avoidance Questionnaire (BEAQ), 24-items Multidimensional Psychological Flexibility Inventory (MPFI-24), Working Alliance Inventory- Short Form (WAI-SF), Difficulties in Emotion Regulation Scale-Short Form (DERS-SF), Self-Compassion Scale-Short Form (SCS-SF), Psychological Insight Questionnaire (PIQ), Emotional Breakthrough Inventory (EBI), Altered States of Consciousness Rating Scale (ASC), and Acceptance/Avoidance-Promoting Experiences Questionnaire (APEQ) (Figure 2).

The **Clinician-Administered PTSD Scale for DSM-5 (CAPS-5)** is a semi-structured clinical interview used to assess index history of DSM-5-defined traumatic event exposure (American Psychiatric Association, 2013), including the most distressing event, time since exposure, and total number of exposures, as well as frequency and severity of posttraumatic stress symptoms, as evidenced by CAPS-5 total score. The CAPS-5 provides diagnostic status (presence versus absence) of PTSD as well as PTSD symptom severity. The CAPS-5 has shown good psychometric properties, including strong internal consistency, interrater reliability, and test-retest reliability [(69)](https://www.zotero.org/google-docs/?nzbbb3).

The **PTSD Checklist-5 (PCL-5)**, a self-report measure designed to follow DSM-5 criteria for assessing PTSD. The PCL-5 is a 20-item self-report questionnaire in which respondents indicate the presence and severity of PTSD symptoms, derived from the DSM-5 symptoms of PTSD (American Psychiatric Association, 2013). Participants indicate how much distress they have experienced due to symptoms such as “Repeated, disturbing memories, thoughts, or images of a stressful experience from the past,” “Trouble remembering important parts of a stressful experience from the past,” and “Feeling irritable or having angry outbursts” on a five-point Likert-type scale (1 = not at all, 5 = extremely). The total PCL-5 score (a sum of all 20 items) provides an index of overall PTSD symptom severity. The PCL-5 has been found to be psychometrically sound, with strong internal consistency, high test-retest reliability, good convergent and discriminant validity [(70,71)](https://www.zotero.org/google-docs/?vUUrVD).

The **Patient Health Questionnaire-9 (PHQ-9)** is used to assess depressive symptom severity. The PHQ-9 includes 9 items which correspond with the nine diagnostic criteria for DSM-5 major depressive disorder. Scores can indicate either no depression, minimal, mild, moderate, moderately severe, or severe depression. There is strong evidence supporting the validity of the PHQ-9 in both the general population and in medical settings. The PHQ-9 also demonstrates good sensitivity to treatment-associated changes in depressive symptom severity [(72,73)](https://www.zotero.org/google-docs/?16FRtA).

The **Generalized Anxiety Disorder Scale, 7-item (GAD-7)** is useful in primary care and mental health settings as a screening tool and symptom severity measure for the seven most common anxiety disorders. Participants choose one of 4 severity scores associated with problems related to the common anxiety disorders and then indicate the degree to which these problems caused functional and/or social difficulties. Scores are determined by calculating the values for each column. A total score is obtained by the sum of all total column values [(74)](https://www.zotero.org/google-docs/?EtQP45).

The **Dissociative Experiences Scale II (DES-II)** is a 28-item, self-report measure of dissociative experiences. Dissociation is often considered a psychological defense mechanism for victims of traumatizing events, and the scale is of particular use in measuring dissociation among people with PTSD [(75)](https://www.zotero.org/google-docs/?rwaM9V).

The **Pittsburgh Sleep Quality Index (PSQI)** is a self-rated questionnaire which assesses sleep quality and disturbances over a 1-month time interval. It consist of 19 items which are grouped into 7 components, including (1) sleep duration, (2) sleep disturbance, (3) sleep latency, (4) daytime dysfunction due to sleepiness, (5) sleep efficiency, (6) overall sleep quality, and (7) sleep medication use. Each of the sleep components yields a score ranging from 0 to 3, with 3 indicating the greatest dysfunction. The sleep component scores are summed to yield a total score ranging from 0 to 21 with the higher total score (referred to as global score) indicating worse sleep quality. In distinguishing good and poor sleepers, a global PSQI score > 5 yields a sensitivity of 89.6% and a specificity of 86.5% [(76,77)](https://www.zotero.org/google-docs/?Am3at3).

The **World Health Organization Well-Being Index, 5-item (WHO-5)** is a short and generic global rating scale measuring subjective well-being. The WHO-5 items are: (1) ‘I have felt cheerful and in good spirits', (2) ‘I have felt calm and relaxed', (3) ‘I have felt active and vigorous', (4) ‘I woke up feeling fresh and rested' and (5) ‘My daily life has been filled with things that interest me'. The respondent is asked to rate how well each of the 5 statements applies to him or her when considering the last 14 days. Each of the 5 items is scored from 5 (all of the time) to 0 (none of the time). The raw score therefore theoretically ranges from 0 (absence of well-being) to 25 (maximal well-being) [(78)](https://www.zotero.org/google-docs/?b01brn).

The **Quality of relationships inventory (QRI)** is a self-report questionnaire used to determine the quality relationships consisting of 25 items that are evaluated on a 4-point Likert scale ranging from 1 = not true to 4 = almost always true. The 25 items yield three dimensions: Support (7 items, e.g., ‘To what extent could you turn to this person for advice about problems?’), conflict (12 items, e.g., ‘How often do you have to work hard to avoid conflict with this person?’), and depth (6 items, e.g., ‘How significant is this relationship in your life?’). The QRI takes about 5 minutes to administer [(79)](https://www.zotero.org/google-docs/?yKuTDH).

The **Inventory of psychosocial functioning (IPF)** is an 80-item self-report instrument measuring PTSD-related functional impairment in the past 30 days. There are seven functional domains evaluated: romantic relationships, family relationships, work, friendships and socializing, parenting, education, and self-care. The IPF was developed to have high content validity, to not confound PTSD symptoms and related impairment, and to not require respondent attributions regarding the cause of impairment [(80)](https://www.zotero.org/google-docs/?HTmiCY).

The **Posttraumatic Maladaptive Beliefs Scale (PMBS)** is a 15-item scale that was developed to measure maladaptive beliefs about life circumstances that may occur following trauma exposure. The scale comprises three subscales: Threat of Harm, Self-Worth and Judgment, and Reliability and Trustworthiness of Others. Scores on each subscale can be derived by summing items within each subscale. A list of subscale items and reverse-code directions are indicated on the measure. Possible scores range from 15-105, and subscale scores range from 5-35 [(81)](https://www.zotero.org/google-docs/?Ptlf2e).

The **Brief Experiential Avoidance Questionnaire (BEAQ)** is a 15-item self-report measure of experiential avoidance, including items such as “The key to a good life is never feeling any pain” and “I work hard to keep out upset feelings”. Participants rate the extent to which they agree with each item on a 6-point scale from 1 (strongly disagree) to 6 (strongly agree), with one item reverse scored. The BEAQ was developed as a short-form of the original Multidimensional Experiential Avoidance Questionnaire (MEAQ). The BEAQ has been shown to have adequate internal consistency (Cronbach’s alpha = 0.80 to 0.84) and good discriminant validity [(82)](https://www.zotero.org/google-docs/?wswq7t).

The **24-items Multidimensional Psychological Flexibility Inventory (MPFI-24)** is a 24-item self-report scale developed to assess the specific components of psychological flexibility and inflexibility proposed in the Hexaflex model of Acceptance and Commitment Therapy (ACT) [(83)](https://www.zotero.org/google-docs/?fX9mcg).

The **Working Alliance Inventory- Short Form (WAI-SF)** is a patient-rated measure of the alliance between a therapist and client. The measure contains a total score and three subscales: agreement on tasks (e.g., "There is a need to clarify the purpose of the session."), agreement on goals (e.g., "There is a shared perception of the client’s goals in therapy"), and development of bonds (e.g., "There is a sense of discomfort in the relationship") [(84)](https://www.zotero.org/google-docs/?2W4edB).

The **Difficulties in Emotion Regulation Scale-Short Form (DERS-SF)** is an 18-item measure used to identify emotional regulation issues in adults. The measure covers 4 dimensions of emotional regulation: awareness and understanding of emotions; acceptance of emotions; the ability to engage in goal-directed behavior and refrain from impulsive behavior when experiencing negative emotions; and access to emotion regulation strategies perceived as effective. The measure has six subscales: Nonacceptance of emotional responses, difficulty engaging in goal-directed behavior, impulse control difficulties, lack of emotional awareness, limited access to emotion regulation strategies, and lack of emotional clarity [(85)](https://www.zotero.org/google-docs/?54nHp0).

The **Self-Compassion Scale-Short Form (SCS-SF)** is a 12-item self-report measure of self-compassion. The measure can be calculated as a total score of self-compassion or the following subscales: Self-Kindness, Self-Judgment, Common Humanity, Isolation, Mindfulness, and Over-Identification. Items are responded to on a scale from 1 (almost never) to 5 (almost always). The SCS-SF was developed as a short-form of the original Self-Compassion Scale (Neff, 2003). The SCS-SF has been shown to have strong validity, including a strong relationship with subscales from the original SCS (i.e., r > .97), as well as good internal consistency (Cronbach’s alpha > 0.86) [(86)](https://www.zotero.org/google-docs/?zgBQEf).

The **Psychological Insight Questionnaire (PIQ)** is a 23-item self-report measure of acute experiences of insight (e.g. awareness into emotions, behaviors, beliefs, memories, or relationships) after taking a psychedelic. The PIQ yields two subscales: (a) avoidance and maladaptive patterns insights (e.g., "Discovered I could explore uncomfortable or painful feelings I previously avoided") and (b) goals and adaptive patterns insights (e.g., "Awareness of my life purpose, goals, and/or priorities"). Participants rate the intensity with which they experienced these forms of insight during their acute psychedelic experience on a Likert-type scale from 0 (No, not at all) to 5 (Extremely). Higher scores indicate a higher level of acute psychological insight. The PIQ shows good internal consistency, discriminant validity from other measures of acute psychedelic experiences, and strong predictive validity in terms of post-psychedelic improvements in well-being and life satisfaction [(87)](https://www.zotero.org/google-docs/?5LBQJH).

The **Emotional Breakthrough Inventory (EBI)** is a recently validated measure of emotional release/breakthrough experienced during the acute psychedelic state. It is a reliable and validated scale that is positively associated with increases in well-being after a psychedelic experience. The EBI consists of eight statements such as “I felt able to explore challenging emotions and memories” and asks about “emotional release”, “closure”, “emotional breakthrough” and “resolution of conflict”. Participants rated the extent to which they agreed with each statement on a 0–10 scale (with 0 being “No, not more than usually” and 10 being “Yes, entirely or completely”) [(88)](https://www.zotero.org/google-docs/?wVWX99).

The **Altered States of Consciousness Rating Scale (ASC)** measures altered states of consciousness. ASC is used to quantify the qualitative subjective experience of psychedelics [(89)](https://www.zotero.org/google-docs/?wqYQ95).

The **Acceptance/Avoidance-Promoting Experiences Questionnaire (APEQ)** is a psychometric self-report tool for measuring aspects of the acute psychedelic experience that are associated with longer-term changes in psychological flexibility [(90)](https://www.zotero.org/google-docs/?k5yh0b).

**3.10.3. Exploratory Outcomes Measures**

1. Digital physiological passive data collected through the use of a wearable device (Oura Ring).
2. Digital phenotype profile (DPP):
3. Personal digital phenotype profile (pDPP) will be constructed based on the wearable and clinical assessment data.

See section (3.7) for the measures used to evaluate the effect of psilocybin-assisted CPT on digital physiological passive data collected through the use of a wearable device (Oura Ring).

###### **3.10.4. Safety Outcome Measures**

Safety measures will be applied as described below to minimize risks associated with study participation. The safety of participants will be assured before, during and after the psilocybin dosing session and during the study period by assessing physiological effects, psychological distress, concomitant medication use, medical events, spontaneously reported reactions, and suicidality. Therapists and study physician will be available via mobile phone throughout the study to ensure participant safety. Vital Signs (blood pressure, pulse, and temperature), Columbia Suicide Severity Rating Scale (C-SSRS), AEs and spontaneously reported reactions will be collected. All AEs, TEAEs, SAEs and AESIs will be collected from day 1 of treatment week until study termination. AEs may be collected during in-person visits or over the telephone or Zoom.

The **Columbia-Suicide Severity Rating Scale (C-SSRS)** is a clinician-administered measure of suicidal behavior devised to detect potential suicidal thoughts or behaviors during a clinical trial. It consists of a “Baseline” form that will assess suicidal ideation, ideation intensity, and behavior in the past 6 months, and a form for assessing current suicidal ideation and behavior. The C-SSRS consists of a series of questions and can be administered during a face-to-face interview or over the telephone. Suicidal ideation and behavior will be assessed at all visits. The C-SSRS Intensity scale for Lifetime obtained a Cronbach’s alpha of 0.93 and 0.94 for the Since Last Visit form, and Last Visit C-SSRS severity scores were positively correlated with the BDI “suicide thoughts” item [(91)](https://www.zotero.org/google-docs/?6SV43q).

**Figure 2.** Summarizes the study aims and presents the outcome measures for each aim.


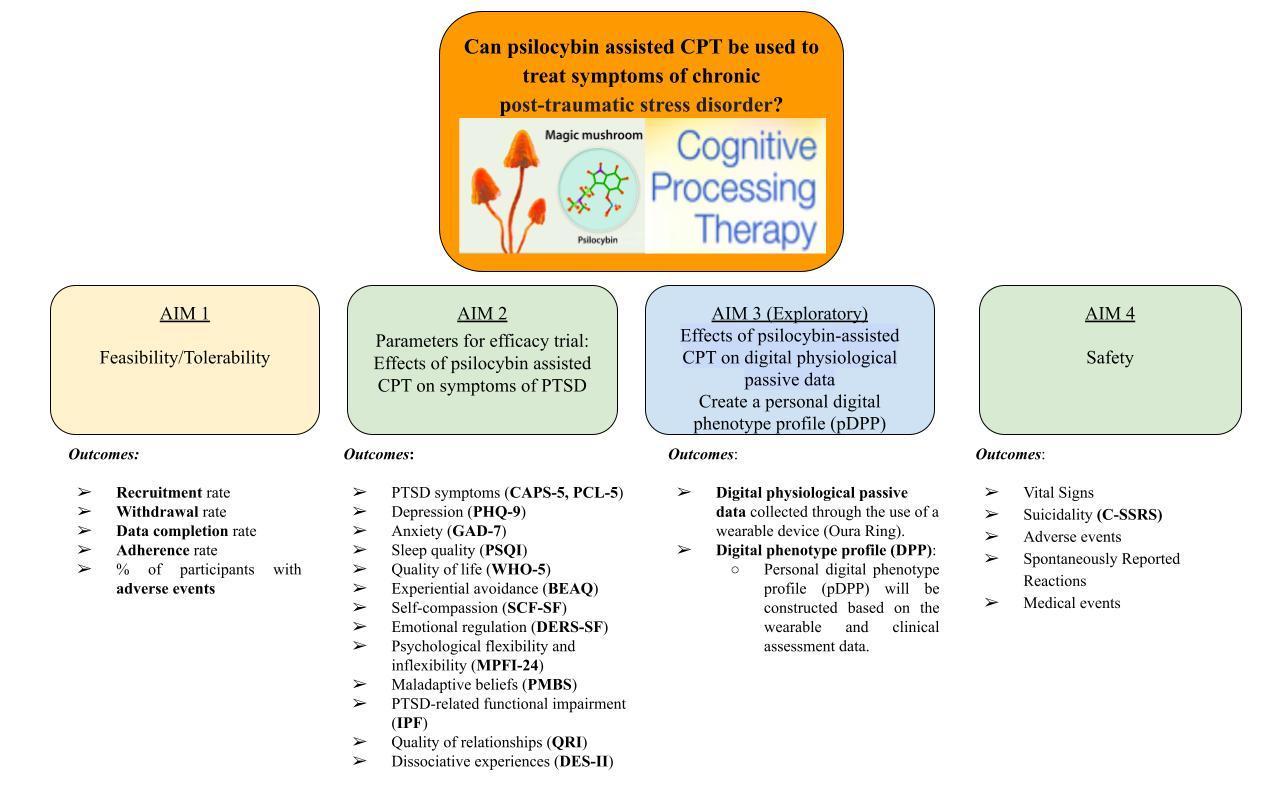


###### **3.11. Quality Assurance**

Methods for quality assurance will be strictly observed. A start-up meeting involving all study site research coordinators, co-investigators, and partners will be held prior to the study launch. Further training will be held at each site prior to the commencement of the study. During training, great care will be taken to fully explain the study procedures and CRF completion.

###### **3.12. Participant Timeline**

**Table 2**. Schedule of Assessment.

| **Study Phases** | **Screening** | **Eligibility confirmation** | **Pre-baseline wearable monitoring (Oura)**** | | | **Baseline** | **Treatment Phase** | | | | | | | | **Follow-up** | | | | | | | | | | | |
| --- | --- | --- | --- | --- | --- | --- | --- | --- | --- | --- | --- | --- | --- | --- | --- | --- | --- | --- | --- | --- | --- | --- | --- | --- | --- | --- |
|  |  |  | **Week 1** | **Week 2** | **Week 3** |  | **Pre-Dose Period** | | **Dosing Day** | | **Post-Dose Period** | | | |  |  |  |  |  |  |  |  |  |  |  |  |
| **Assessment Days** |  |  |  |  |  |  | **Day 1** | **Day 2** | **Day 3**  **pre dose** | **Day 3 post dose** | **Day 4** | **Day 5** | **Day 6** | **Day 7** | **Week 2** | **Week 3** | **Week 4** | **Week 5** | **Week 6** | **Week 7** | **Week 8** | **Week 9** | **Week 10** | **Week 11** | **Week 12** | **Week 13** |
| **Screening** | | | | | | | | | | | | | | | | | | | | | | | | | | |
| **ICF signed** | **╳** |  |  |  |  |  |  |  |  |  |  |  |  |  |  |  |  |  |  |  |  |  |  |  |  |  |
| **Exclusion/inclusion criteria** | **╳** | **╳** |  |  |  |  |  |  |  |  |  |  |  |  |  |  |  |  |  |  |  |  |  |  |  |  |
| **Patient demographics and medical history** | **╳** |  |  |  |  |  |  |  |  |  |  |  |  |  |  |  |  |  |  |  |  |  |  |  |  |  |
| **Physical examination*** | **╳** |  |  |  |  |  |  |  |  |  |  |  |  |  |  |  |  |  |  |  |  |  |  |  |  |  |
| **Vital signs** | **╳** |  |  |  |  |  |  |  | **╳** | **╳** |  |  |  |  |  |  |  |  |  |  |  |  |  |  |  |  |
| **Contraceptive method review** | **╳** | **╳** |  |  |  | **╳** |  |  | **╳** |  |  |  |  |  |  |  |  |  |  |  |  |  |  |  |  |  |
| **Pregnancy screening** | **╳** |  |  |  |  |  |  |  | **╳** |  |  |  |  |  |  |  |  |  |  |  |  |  |  |  |  |  |
| **Urine & Blood Tests *** | **╳** |  |  |  |  |  |  |  |  |  |  |  |  |  |  |  |  |  |  |  |  |  |  |  |  |  |
| **C-SSRS lifetime and past month** | **╳** |  |  |  |  |  |  |  |  |  |  |  |  |  |  |  |  |  |  |  |  |  |  |  |  |  |
| **MINI** | **╳** |  |  |  |  |  |  |  |  |  |  |  |  |  |  |  |  |  |  |  |  |  |  |  |  |  |
| **Intervention** | | | | | | | | | | | | | | | | | | | | | | | | | | |
| **Psilocybin** |  |  |  |  |  |  |  |  | **╳** | **╳** |  |  |  |  |  |  |  |  |  |  |  |  |  |  |  |  |
| **Therapeutic Sessions** | | | | | | | | | | | | | | | | | | | | | | | | | | |
| **CPT (x2)** |  |  |  |  |  |  | **╳** | **╳** |  |  | **╳** | **╳** | **╳** | **╳** |  |  |  |  |  |  |  |  |  |  |  |  |
| **Preparatory Session** |  |  |  |  |  |  |  | **╳** |  |  |  |  |  |  |  |  |  |  |  |  |  |  |  |  |  |  |
| **Integration Session** |  |  |  |  |  |  |  |  |  |  | **╳** |  |  |  |  |  |  |  |  |  |  |  |  |  |  |  |
| **Efficacy** | | | | | | | | | | | | | | | | | | | | | | | | | | |
| **CAPS-5** | **╳** |  |  |  |  | **╳** |  |  |  |  |  |  |  | **╳** | **╳** |  |  | **╳** |  |  |  |  |  |  |  | **╳** |
| **PCL-5** | **╳** |  | **╳** | **╳** | **╳** | **╳** | **╳** | **╳** | **╳** |  | **╳** | **╳** | **╳** | **╳** | **╳** | **╳** | **╳** | **╳** | **╳** | **╳** | **╳** | **╳** | **╳** | **╳** | **╳** | **╳** |
| **PHQ-9** |  |  | **╳** | **╳** | **╳** | **╳** | **╳** | **╳** | **╳** |  | **╳** | **╳** | **╳** | **╳** | **╳** | **╳** | **╳** | **╳** | **╳** | **╳** | **╳** | **╳** | **╳** | **╳** | **╳** | **╳** |
| **GAD-7** |  |  | **╳** | **╳** | **╳** | **╳** | **╳** | **╳** | **╳** |  | **╳** | **╳** | **╳** | **╳** | **╳** | **╳** | **╳** | **╳** | **╳** | **╳** | **╳** | **╳** | **╳** | **╳** | **╳** | **╳** |
| **WHO-5** |  |  | **╳** | **╳** | **╳** | **╳** | **╳** | **╳** | **╳** |  | **╳** | **╳** | **╳** | **╳** | **╳** | **╳** | **╳** | **╳** | **╳** | **╳** | **╳** | **╳** | **╳** | **╳** | **╳** | **╳** |
| **WAI-SF** |  |  |  |  |  |  | **╳** | **╳** | **╳** | **╳** | **╳** | **╳** | **╳** | **╳** |  |  |  |  |  |  |  |  |  |  |  |  |
| **DES-II** |  |  |  |  |  | **╳** |  |  |  |  |  |  |  | **╳** |  |  |  | **╳** |  |  |  |  |  |  |  | **╳** |
| **PSQI** |  |  |  |  |  | **╳** |  |  |  |  |  |  |  | **╳** |  |  |  | **╳** |  |  |  |  |  |  |  | **╳** |
| **QRI** |  |  |  |  |  | **╳** |  |  |  |  |  |  |  | **╳** |  |  |  | **╳** |  |  |  |  |  |  |  | **╳** |
| **IPF** |  |  |  |  |  | **╳** |  |  |  |  |  |  |  | **╳** |  |  |  | **╳** |  |  |  |  |  |  |  | **╳** |
| **PMBS** |  |  |  |  |  | **╳** |  |  |  |  |  |  |  | **╳** |  |  |  | **╳** |  |  |  |  |  |  |  | **╳** |
| **BEAQ** |  |  |  |  |  | **╳** |  |  |  |  |  |  |  | **╳** |  |  |  | **╳** |  |  |  |  |  |  |  | **╳** |
| **MPFI-24** |  |  |  |  |  | **╳** |  |  |  |  |  |  |  | **╳** |  |  |  | **╳** |  |  |  |  |  |  |  | **╳** |
| **DERS-SF** |  |  |  |  |  | **╳** |  |  |  |  |  |  |  | **╳** |  |  |  | **╳** |  |  |  |  |  |  |  | **╳** |
| **SCS-SF** |  |  |  |  |  | **╳** |  |  |  |  |  |  |  | **╳** |  |  |  | **╳** |  |  |  |  |  |  |  | **╳** |
| **CTQ-SF** |  |  |  |  |  | **╳** |  |  |  |  |  |  |  |  |  |  |  |  |  |  |  |  |  |  |  |  |
| **PIQ** |  |  |  |  |  |  |  |  |  | **╳** |  |  |  |  |  |  |  |  |  |  |  |  |  |  |  |  |
| **EBI** |  |  |  |  |  |  |  |  |  | **╳** |  |  |  |  |  |  |  |  |  |  |  |  |  |  |  |  |
| **ASC** |  |  |  |  |  |  |  |  |  | **╳** |  |  |  |  |  |  |  |  |  |  |  |  |  |  |  |  |
| **APEQ** |  |  |  |  |  |  |  |  |  | **╳** |  |  |  |  |  |  |  |  |  |  |  |  |  |  |  |  |
| **Oura Ring** |  | **╳** | **╳** | **╳** | **╳** | **╳** | **╳** | **╳** | **╳** | **╳** | **╳** | **╳** | **╳** | **╳** | **╳** | **╳** | **╳** | **╳** | **╳** | **╳** | **╳** | **╳** | **╳** | **╳** | **╳** | **╳** |
| **Qualitative survey** |  |  |  |  |  |  |  |  |  |  |  |  |  |  |  |  |  | **╳** |  |  |  |  |  |  |  | **╳** |
| **Safety and tolerability** | | | | | | | | | | | | | | | | | | | | | | | | | | |
| **Adverse events** |  |  |  |  |  |  | **╳** | **╳** | **╳** | **╳** | **╳** | **╳** | **╳** | **╳** | **╳** | **╳** | **╳** | **╳** | **╳** | **╳** | **╳** | **╳** | **╳** | **╳** | **╳** | **╳** |
| **Concomitant medications** | **╳** | **╳** | **╳** | **╳** | **╳** | **╳** | **╳** | **╳** | **╳** |  | **╳** | **╳** | **╳** | **╳** | **╳** | **╳** | **╳** | **╳** | **╳** | **╳** | **╳** | **╳** | **╳** | **╳** | **╳** | **╳** |
| **C-SSRS since last visit** |  | **╳** | **╳** | **╳** | **╳** | **╳** | **╳** | **╳** | **╳** | **╳** | **╳** | **╳** | **╳** | **╳** | **╳** | **╳** | **╳** | **╳** | **╳** | **╳** | **╳** | **╳** | **╳** | **╳** | **╳** | **╳** |

**Abbreviations:** CAPS-5: Clinician-Administered PTSD Scale for DSM-5, PCL-5: PTSD Checklist-5, PHQ-9: Patient Health Questionnaire-9, DES-II: Dissociative Experiences Scale II, PSQI: Pittsburgh Sleep Quality Index, QRI: Quality of Relationships Inventory, IPF: Inventory of psychosocial functioning, PMBS: Posttraumatic Maladaptive Beliefs Scale, BEAQ: Brief Experiential Avoidance Questionnaire, MPFI-24: 24 items Multidimensional Psychological Flexibility Inventory, WAI-SF: Working Alliance Inventory- Short Form, DERS-SF: Difficulties in Emotion Regulation Scale-Short Form, SCS-SF: Self-compassion scale short form, PIQ: Psychological Insight Questionnaire, EBI: Emotional Breakthrough Inventory, ASC: Altered States of Consciousness Rating Scale, APEQ: Acceptance/Avoidance-Promoting Experiences Questionnaire, GAD-7: Generalized Anxiety Disorder Scale, 7-item, CTQ-SF: Childhood Trauma Questionnaire-short form, WHO-5: World Health Organization Well-Being Index, 5-item, CPT: Cognitive Processing Therapy, MINI: Mini International Neuropsychiatric Interview. * See section 3.13.2 for a thorough description. **Participants require to use a wearable (Oura) at least 3 weeks before baseline.

###### **3.13. Trial Procedures and Evaluations**

###### **3.13.1. Pre-Study Screening**

Potential participants will access a screening survey through REDCap which will be a preliminary measure of their eligibility for the study. Once a participant has completed the REDCap screening form, participants who appear eligible will be contacted via phone for a pre-screening call to give them additional background information on the study and associated participant responsibilities. The study coordinator will also complete the Clinician-Administered PTSD Scale for DSM-5 (CAPS-5) and Posttraumatic Stress Disorder Checklist-4 (PCL-4) over the phone. Participants will be sent the informed consent form (ICF) via email after the call. If participants agree to participate in the study, they will email the signed ICF to the study team. Participants who meet all major inclusion/exclusion criteria will be invited to the Interventional Psychiatry Program at St. Michael’s Hospital for an in-person screening. Eligibility will be assessed during the screening and eligibility confirmation visits, which will be scheduled during the pre-screening call. Any questions will be answered at this point. The timeline of visits will then be explained. Additionally, the use of the wearable device will be explained. The participant will be asked if they have any additional questions. Once all questions have been answered, the participant will be asked if they are still interested in participating in the study. The in-person screening visit will then be scheduled.

###### **3.13.2. Screening**

Participants will be screened initially to evaluate eligibility for the study at the Interventional Psychiatry Program at St. Michael’s Hospital. All participants will be seen approximately four weeks prior to the first therapy session. The screening visit will last around 2-3 hours. Before any study procedures are undertaken, participants will be given time to ask any questions, and written consent will be obtained by the study coordinator. The participant will also be sized for the wearable device to confirm that the appropriate size is available. However, they will not be given the ring at this visit - should they meet eligibility criteria after review, they will be given the appropriate sized wearable device at their next visit (see section 3.13.3 Eligibility Confirmation).

During the screening visit, all participants that have consented to receiving psilocybin-assisted CPT will:

1. Undergo a physical examination and the measurement of vitals. The examination will involve the following procedures:

- Blood pressure
- Pulse
- Height
- Weight
- Body temperature
- Examination of head, eyes, ears, nose, throat, skin, heart, lungs, abdomen, and extremities
- Brief neurological exam (cranial nerves 2-12, sensory, motor, reflexes and cerebellar function)
- Electrocardiogram (ECG) and 1-minute rhythm strip

1. Provide demographic information and review of medical, psychiatric, and medication history, including review and confirmation of diagnosis.
2. Complete pre-study screening blood work including:

- Hematology: hemoglobin, haematocrit, red blood cell count, white blood cell count (with differential), and platelet count.
- Biochemistry: albumin, alkaline phosphatase, alanine aminotransferase (ALT), amylase, aspartate aminotransferase (AST), bilirubin (total), calcium, cholesterol (HDL, LDL), creatinine, glucose, glycated hemoglobin (HbA1c), potassium, protein (total), triglycerides, sodium, urea (blood urea nitrogen), and uric acid. hCG pregnancy screen (if applicable).
- Immunoassay: insulin.
- Immunology: C-reactive protein.

1. Complete urine to test for certain substances such as non-prescribed drugs. Drug screen results will be kept confidential and will only be disclosed if required by law.
2. Complete the Clinician-Administered PTSD Scale for DSM-5 (CAPS-5). In order for patients to be eligible for the study, their total score should be ≥ 50 (moderate-to-severe PTSD).
3. Complete Columbia Suicide Severity Rating Scale (C-SSRS) lifetime and past month.
4. Complete the Mini International Neuropsychiatric Interview (MINI).
5. Complete Posttraumatic Stress Disorder Checklist-5 (PCL-5).
6. Contraceptive method review.

The routine blood and urine tests will be done to ensure that there are no medical reasons that may make participants ineligible for the study. The purpose of this laboratory test is not for further research, but rather for safety and risk monitoring. If a participant tests positive for pregnancy, they will not be enrolled in the study. Any positive urinary drug test findings that cannot be attributed to pre-approved concomitant medications or diet will be reviewed by the study physician to assess compliance with ongoing eligibility criteria. A full list of screening assessments can be found in Table 2.

###### **3.13.3. Eligibility confirmation**

Eligible participants will be seen in person approximately a week after the screening visit (approximately 3 weeks before baseline) to confirm eligibility and receive the wearable device and access to the wearable app and web-based platform. During or prior to this visit, the study doctor will review laboratory test results and confirm that eligibility criteria are met. Once eligibility is confirmed, the study coordinator will assist the participant with the setup of the wearable app and web-based platform. Participants will be given study email credentials (dummy email address and password), which they will use to log-in to the Oura App. Email forwarding from the dummy email to the participants preferred email account will be set up. The study coordinator will provide an explanation of how to use the Oura app and the web-based platform and answer any questions the participant might have. Participants will wear the ring for approximately 3 weeks after this visit before beginning treatment, which will be referred to as the baseline period. This baseline period is necessary to ensure accurate data collection by the Oura Ring and to establish baseline parameters. Moreover, during this visit, the following items will be collected:

- Concomitant medications
- Columbia Suicide Severity Rating Scale (C-SSRS) since last visit
- Contraceptive method review
- Exclusion/inclusion criteria review

###### **3.13.4. Pre-baseline wearable monitoring (Oura)**

Participants will complete the following scales weekly (week 1-3) between eligibility confirmation and baseline visits for digital monitoring:

- Concomitant medications
- Columbia Suicide Severity Rating Scale (C-SSRS) since last visit
- Posttraumatic Stress Disorder Checklist for the DSM-5 (PCL-5)
- Patient Health Questionnaire-9 (PHQ-9)
- Generalized Anxiety Disorder Scale, 7-item (GAD-7)
- World Health Organization Well-Being Index, 5-item (WHO-5)

###### **3.13.5. Baseline**

Participants will complete baseline assessments one day before day 1. During this visit, baseline scores for the following items will be collected:

- Concomitant medications
- Contraceptive method review
- Columbia Suicide Severity Rating Scale (C-SSRS) since last visit
- Clinician-Administered PTSD Scale for DSM-5 (CAPS-5)
- Posttraumatic Stress Disorder Checklist for the DSM-5 (PCL-5)
- Patient Health Questionnaire-9 (PHQ-9)
- Generalized Anxiety Disorder Scale, 7-item (GAD-7)
- Dissociative Experiences Scale II (DES-II)
- Pittsburgh Sleep Quality Index (PSQI)
- Quality of Relationships Inventory (QRI)
- World Health Organization Well-Being Index, 5-item (WHO-5)
- Psychosocial Functioning (IPF)
- Posttraumatic Maladaptive Beliefs Scale (PMBS)
- Brief Experiential Avoidance Questionnaire (BEAQ)
- 24 items Multidimensional Psychological Flexibility Inventory (MPFI-24)
- Difficulties in Emotion Regulation Scale-Short Form (DERS-SF)
- Self-Compassion Scale-Short Form (SCS-SF)
- Childhood Trauma Questionnaire-short form (CTQ-SF)

###### **3.13.6. Day 1, 2**

On days 1 and 2, CPT sessions 1-4 and the 45-minute preparatory session (on day 2) will be completed. Also, PTSD Checklist-5 (PCL-5), Patient Health Questionnaire-9 (PHQ-9), Working Alliance Inventory-Short Form (WAI-SF), World Health Organization Well-Being Index, 5-item (WHO-5), Generalized Anxiety Disorder Scale, 7-item (GAD-7), Columbia Suicide Severity Rating Scale (C-SSRS) since last visit, AEs and concomitant medications review will be administered virtually by the study coordinator.

###### **3.13.7. Day 3 (Dosing Day)**

25 mg psilocybin will be administered on day 3 (6-8 hours). The following scales will be collected in person:

| **Pre-dose assessments** | **Post-dose assessments** |
| --- | --- |
| - **AEs** - **Concomitant medications** - **Vital signs** - **Contraceptive method review** - **Pregnancy screening through a personal urine test** - **Columbia Suicide Severity Rating Scale (C-SSRS) since last visit** - **Posttraumatic Stress Disorder Checklist for the DSM-5 (PCL-5)** - **Patient Health Questionnaire-9 (PHQ-9)** - **Working Alliance Inventory- Short Form (WAI-SF)** - **World Health Organization Well-Being Index, 5-item (WHO-5)** - **Generalized Anxiety Disorder Scale, 7-item (GAD-7)** | - **Columbia Suicide Severity Rating Scale (C-SSRS) since last visit** - **AEs** - **Vital signs** - **Working Alliance Inventory- Short Form (WAI-SF)** - **Psychological Insight Questionnaire (PIQ)** - **Emotional Breakthrough Inventory (EBI)** - **Altered States of Consciousness Rating Scale (ASC)** - **Acceptance/Avoidance-Promoting Experiences Questionnaire (APEQ)** |

Post dose assessments will be completed after the acute effects of psilocybin are resolved. Upon completion of the post-dose assessments, the participant will be assessed for safe discharge under the supervision of a designated chaperone.

###### **3.13.8. Day 4-7**

The 1-hour integration session and CPT sessions 5 and 6 will be completed virtually on the day after the dosing session (day 4). Moreover, CPT sessions 7–12 will be completed virtually twice from day 5 to day 7. The follow-up assessments are specified in the Schedule of Assessments (Table 2).

###### **3.13.9. Follow-up Assessments**

The follow-up assessments as specified in the Schedule of Assessments (Table 2) will be conducted weekly from week 2 until week 13. Week 2 to week 12 follow-up assessments will be conducted virtually, and week 13 will be conducted in person. A narrative, qualitative survey at weeks 5 and 13 will evaluate participant experiences with psilocybin-assisted CPT and the wearable device. Participants will return Oura to study coordinator or research assistant at week 13. The interviews will be conducted by the study coordinator or research assistant and take approximately 45-60 minutes to complete. We will also use User Experience Questionnaire.

Qualitative survey questions will include:

1. Think back to yourself before you heard about the study—Can you briefly describe what your experience of posttraumatic stress disorder was like?

2. What happened during the session when you were given the psilocybin? Please describe your experience.

3. What were cognitive processing therapy sessions like for you? Please describe your experience.

4. Has the treatment had any effect, positive or negative, on your posttraumatic stress disorder and life? Have you noticed any changes in yourself, your thinking, your emotions, or your behaviors?

5. What do you attribute those changes to? How do you think the psilocybin experience contributed to these changes? How do you feel the cognitive processing therapy sessions contributed to these changes?

6. Is there anything that you would change about the intervention? What was it like for you to do an intensive one week treatment?

7. Anything else you would like to add about your experience with the treatment or about its effects on your posttraumatic stress disorder?

8. You have also been using the Oura Ring over the past ______ weeks. What was your experience like with this platform? Were there any specific barriers to use that you found? Did you find this platform helped you in understanding your mental state and well-being over the past few weeks?

**Table 3. User Experience Questionnaire.**

|  | 1 | 2 | 3 | 4 | 5 | 6 | 7 |  |
| --- | --- | --- | --- | --- | --- | --- | --- | --- |
| annoying | ⚪ | ⚪ | ⚪ | ⚪ | ⚪ | ⚪ | ⚪ | enjoyable |
| not understandable | ⚪ | ⚪ | ⚪ | ⚪ | ⚪ | ⚪ | ⚪ | understandable |
| creative | ⚪ | ⚪ | ⚪ | ⚪ | ⚪ | ⚪ | ⚪ | dull |
| easy to learn | ⚪ | ⚪ | ⚪ | ⚪ | ⚪ | ⚪ | ⚪ | difficult to learn |
| valuable | ⚪ | ⚪ | ⚪ | ⚪ | ⚪ | ⚪ | ⚪ | inferior |
| boring | ⚪ | ⚪ | ⚪ | ⚪ | ⚪ | ⚪ | ⚪ | exciting |
| not interesting | ⚪ | ⚪ | ⚪ | ⚪ | ⚪ | ⚪ | ⚪ | interesting |
| unpredictable | ⚪ | ⚪ | ⚪ | ⚪ | ⚪ | ⚪ | ⚪ | predictable |
| fast | ⚪ | ⚪ | ⚪ | ⚪ | ⚪ | ⚪ | ⚪ | slow |
| inventive | ⚪ | ⚪ | ⚪ | ⚪ | ⚪ | ⚪ | ⚪ | conventional |
| obstructive | ⚪ | ⚪ | ⚪ | ⚪ | ⚪ | ⚪ | ⚪ | supportive |
| good | ⚪ | ⚪ | ⚪ | ⚪ | ⚪ | ⚪ | ⚪ | bad |
| complicated | ⚪ | ⚪ | ⚪ | ⚪ | ⚪ | ⚪ | ⚪ | easy |
| unlikable | ⚪ | ⚪ | ⚪ | ⚪ | ⚪ | ⚪ | ⚪ | pleasing |
| usual | ⚪ | ⚪ | ⚪ | ⚪ | ⚪ | ⚪ | ⚪ | leading edge |
| unpleasant | ⚪ | ⚪ | ⚪ | ⚪ | ⚪ | ⚪ | ⚪ | pleasant |
| secure | ⚪ | ⚪ | ⚪ | ⚪ | ⚪ | ⚪ | ⚪ | not secure |
| motivating | ⚪ | ⚪ | ⚪ | ⚪ | ⚪ | ⚪ | ⚪ | demotivating |
| meets expectations | ⚪ | ⚪ | ⚪ | ⚪ | ⚪ | ⚪ | ⚪ | does not meet expectations |
| inefficient | ⚪ | ⚪ | ⚪ | ⚪ | ⚪ | ⚪ | ⚪ | efficient |
| clear | ⚪ | ⚪ | ⚪ | ⚪ | ⚪ | ⚪ | ⚪ | confusing |
| impractical | ⚪ | ⚪ | ⚪ | ⚪ | ⚪ | ⚪ | ⚪ | practical |
| organized | ⚪ | ⚪ | ⚪ | ⚪ | ⚪ | ⚪ | ⚪ | cluttered |
| attractive | ⚪ | ⚪ | ⚪ | ⚪ | ⚪ | ⚪ | ⚪ | unattractive |
| friendly | ⚪ | ⚪ | ⚪ | ⚪ | ⚪ | ⚪ | ⚪ | unfriendly |
| conservative | ⚪ | ⚪ | ⚪ | ⚪ | ⚪ | ⚪ | ⚪ | innovative |

###### **4. Safety**

The safety of participants will be assured before, during and after the psilocybin dosing session and during the study period by assessing physiological effects, psychological distress, medical events, AEs, TEAEs, SAEs, AESIs, spontaneously reported reactions, concomitant medication use, and suicidal ideation and behavior.

- Suicidality will be assessed with the Columbia Suicide Severity Rating Scale (C-SSRS) throughout the study.
- Vital signs including blood pressure, heart rate and temperature will be measured before and after the dosing session.
- SAEs, TEAEs and SAEs will be collected. All medical events and spontaneously reported reactions will be collected throughout the study.
- Events requiring medical attention will be collected through termination.
- Any event leading to withdrawal from the protocol will be collected throughout the study.
- All events related to changes in psychiatric status will be collected throughout the study.
- Baseline medications and changes to psychiatric medications will be collected throughout the study.

###### **4.1. Adverse Events**

An AE is the development of an undesirable medical condition or the deterioration of a pre-existing medical condition following or during exposure to a pharmaceutical product, whether or not considered causally related to the product. An undesirable medical condition can be symptoms (e.g., nausea, chest pain), signs (e.g., tachycardia, enlarged liver) or the abnormal results of an investigation (e.g., laboratory findings, electrocardiogram). In clinical studies, an AE can include an undesirable medical condition occurring at any time, including run-in or washout periods, even if no study treatment has been administered.

###### **4.1.1. Assessment of Intensity**

Each AE will be classified according to the following criteria:

- Mild: The AE does not interfere in a significant manner with the participant’s normal level of functioning.
- Moderate: The AE produces some impairment of functioning, but is not hazardous to the participant’s health.
- Severe: The AE produces significant impairment of functioning or incapacitation and is a definite hazard to the participant’s health.

When changes in the intensity of an AE occur more frequently than once a day, the maximum intensity for the experience should be noted. If the intensity category changes over several days, those changes should be recorded separately (with distinct onset dates).

###### **4.1.2. Assessment of Causality**

All AEs will be assessed by a blinded study investigator as to whether they can be explained by the participant’s underlying condition. If they are not explainable, then the study investigator will make a determination of the relationship of the AE with psilocybin as follows:

- **Probable:** Reports including good reasons and sufficient documentation to assume a causal relationship, in the sense of plausible, conceivable, likely, but not necessarily highly probable. A reaction that follows a reasonable temporal sequence from administration of psilocybin; that follows a known or expected response pattern to the suspected medicine; or that is confirmed by stopping or reducing the dosage of the medicine and that could not reasonably be explained by known characteristics of the participant’s clinical state.
- **Possible:** Reports containing sufficient information to accept the possibility of a causal relationship, in the sense of not impossible and not unlikely, although the connection is uncertain or doubtful, for example because of missing data or insufficient evidence. A reaction that follows a reasonable temporal sequence from administration of the psilocybin; that follows a known or expected response pattern to the suspected medicine; but that could readily have been produced by a number of other factors.
- **Unlikely:** Reports not following a reasonable temporal sequence from psilocybin administration. An event which may have been produced by the participant’s clinical state or by environmental factors or other therapies administered.
- **Not related (unrelated):** Events for which sufficient information exists to conclude that the etiology is unrelated to the psilocybin.
- **Unclassified:** Reports which for one reason or another are not yet accessible (e.g., because of outstanding information (can only be a temporary assessment)).

###### 4.1.3. Action Taken Regarding Investigational Product

Dose modifications of IP (ie, dose not changed, drug withdrawn, drug interrupted, or dose increased) are not applicable as this is a single dose study.

- Not Applicable: Participant died, study treatment had been completed prior to reaction/event, or reaction/event occurred prior to start of treatment.

Other Action Taken for Event

- 1 = None (ie, no treatment was required)
- 2 = Medication required (ie, prescription and/or OTC medication was required to treat the AE)
- 3 = Hospitalisation or prolongation of hospitalisation required (ie, hospitalisation was required or prolonged because of the AE, whether medication was required)
- 4 = Other

##### 4.1.4. Adverse Event Outcome

- 1 = Recovered/Resolved (ie, the participant fully recovered from the AE with no residual effect observed)
- 2 = Recovering/Resolving (ie, the AE improved but has not fully resolved)
- 3 = Not Recovered/Not Resolved (ie, the AE itself is still present and observable)
- 4 = Recovered/Resolved with Sequelae (ie, the residual effects of the AE are still present and observable, including sequelae/residual effects)
- 5 = Fatal (ie, ‘fatal’ should be used when death is a direct outcome of the AE)
- 6 = Unknown

###### **4.1.5. Severity versus Seriousness**

It is important to distinguish between serious and severe AEs. Severity is used to describe the intensity of a specific event while the event itself, however, may be of relatively minor medical significance (such as a severe headache). This is not the same as "seriousness," which is based on participant/event outcome at the time of the event. An AE of severe intensity need not necessarily be considered serious. For example, nausea that persists for several hours may be considered severe nausea, but not an SAE. On the other hand, a stroke that results in only a limited degree of disability may be considered a mild stroke but would be an SAE. Symptoms associated with overdose should be reported as AEs. For further information regarding overdose, see section 4.1.5.

###### **4.1.6. Recording of Adverse Events**

AEs will be collected from day 1 of treatment week until the end of the study. At each visit, subjects will be asked if they have had any health problems since the previous visit. All AEs will be recorded appropriately, whether or not considered related to the investigational product. This will include AEs spontaneously reported by the patient and/or observed by the staff as well as AEs reported in response to a direct question e.g. “Have you had any health problems since your last visit?”

For each AE, the following parameters will be described: discovery (i.e., spontaneous or solicited); start; end date or ongoing; action taken with regards to investigational product; outcome if the AE caused the patient to discontinue; a statement if the AE fulfills the criteria for a SAE or not; the investigator’s assessment of the causal relationship between the event and the investigational product; intensity of the AE, i.e., mild (awareness of sign or symptom, but easily tolerated), moderate (discomfort sufficient to cause interference with normal activities), severe (incapacitating, with inability to perform normal activities); and resolution.

###### **4.1.7. Overdose**

Any instance of overdose (suspected or confirmed) must be fully documented as an AE or SAE if it meets the SAE criteria. Details of any signs or symptoms and their management should be recorded.

###### **4.1.8. Adverse Events of Special Interest**

An adverse event of special interest (AESI) is an AE (serious or non-serious) of scientific and medical concern specific to the study drug, for which ongoing monitoring and immediate notification by the investigator to the sponsor is required. Such AEs may require further investigation to characterize and understand them.

The following events will be reported as AESI:

- Euphoric mood
- Dissociative disorder
- Hallucination
- Psychotic disorder
- Cognitive disorder
- Disturbance in attention
- Mood altered
- Psychomotor skills impaired
- Inappropriate affect
- Overdose
- Intentional product misuse

###### **4.1.9. Adverse Event Follow-up**

All AEs will be followed until resolved or stable and the outcome documented on the CRF. If the investigator detects an AE in a study participant after the last scheduled follow-up visit and considers the event possibly related or related to prior study treatment, the investigator will document it.

###### **4.1.10. Serious Adverse Events**

A serious adverse event is an AE that fulfills one or more of the following criteria:

- results in death
- is life-threatening
- requires in-patient hospitalization or prolongation of existing hospitalization
- results in persistent or significant disability or incapacity
- is a congenital abnormality or birth defect in the offspring of a participant who received the investigational product
- is an important medical event that may jeopardize the patient or may require medical intervention to prevent one of the outcomes listed above.

Any serious, untoward event that may occur subsequent to the reporting period that the investigator assessed as related to psilocybin should also be reported and managed as an SAE. The investigator should follow participants with AEs until the event has resolved or the condition has stabilized. In case of unresolved AEs, including significant abnormal clinical laboratory values at the end of study assessment, these events will be followed until resolution or until they become clinically not relevant.

Disability/incapacitating: An AE is incapacitating or disabling if the experience results in a substantial and/or permanent disruption of the participant's ability to carry out normal life functions.

###### **4.1.11. Reporting of Serious Adverse Events**

Reporting of SAEs to regulatory authorities will be done by the investigator in accordance with C.05.014 (1) of the Food and Drug Regulations. A copy of the report will also be sent to the manufacturer of the investigational product.

All serious unexpected adverse drug reactions in respect of a drug during the course of this clinical trial will be reported to Health Canada in an expedited manner. Information about serious unexpected adverse drug reactions that are neither fatal nor life threatening will be submitted to Health Canada within 15 calendar days after becoming aware of the event. All serious unexpected adverse drug reactions that are fatal or life threatening will be reported to Health Canada within 7 calendar days after becoming aware of the event. In cases where the event is fatal or life threatening, the sponsor will submit a complete report to Health Canada within 8 calendar days after the first notification (initial report) to Health Canada of the event. Follow-up reports of fatal or life threatening reactions will include an assessment of the importance of the event and the implication of any findings, including relevant previous experience with the same or similar drugs.

###### **4.2. Permissible Medications**

The following restrictions may apply to the use of non-psychiatric drugs that may have a psychotropic impact, as well as medications for the management of concurrent anxiety and sleeplessness. From screening to the end-of-study visit (week 13), participants are permitted adjunctive use of benzodiazepines: up to 2 mg of lorazepam or equivalent per day for anxiety and insomnia, though these medications must be held for 12 hours prior to psilocybin administration. Prescription and nonprescription medications with potential psychotropic effects used for nonpsychiatric conditions (e.g., pseudoephedrine for cold and allergy symptoms) should not be used more than two times per week and must be held for 24 hours prior to psilocybin administration. Documentation of adjunctive anxiolytics, hypnotics, or medications with psychotropic properties (including over-the-counter medications) will be recorded in the concomitant medication log at each study assessment. Rescue medications used in the event of AE management as specified in this protocol are permissible and will be recorded in the concomitant medication log.

###### **4.3. Prohibited Medications**

Psilocybin is contraindicated in participants who are on monoamine oxidase inhibitors or who have a known sensitivity to the drug or its metabolites. It is contraindicated in participants taking medications that are known as uridine diphosphate glucuronosyltransferase enzyme modulators. It is contraindicated in patients with schizophrenia or bipolar disorder, or in those with first degree relatives with these disorders. The concurrent use of selective serotonin reuptake inhibitors (SSRIs), serotonin-norepinephrine reuptake inhibitors (SNRIs) medications is assumed to be contraindicated due to the potential to increase the risk of serotonin syndrome and/or to attenuate the binding of psilocin to the HT2A receptor.

Participants must discontinue medications of medication classes listed in Table 4 at least 5 half-lives before baseline. Since the anticipated half-life of psilocybin is approximately 3 hours, no known issues related to pharmacokinetics or pharmacodynamic interactions are expected within approximately 7 days post-dosing. Common medications in these classes are noted in Table 4, but this list is not exhaustive. These medications must not be reintroduced to the participant until after end-of-study. The decision to discontinue these medications will be made by the participant and study psychiatrist.

**Table 4.** Prohibited Medications (Not an exhaustive list).

| Class | Common Medications |
| --- | --- |
| Selective-Serotonin Reuptake Inhibitors (SSRIs) | Citalopram  Escitalopram  Fluoxetine  Fluvoxamine  Paroxetine  Sertraline  Vilazadone  Vortioxetine |
| Selective-Norepinephrine Reuptake Inhibitors (SNRIs) | Desvenlafaxine  Duloxetine  Levomilnacipran  Milnacipran  Venlafaxine |
| Tricyclic Antidepressants (TCAs) | Amitriptyline  Amitriptylinoxide  Amoxapine  Clomipramine  Desipramine  Dibenzepin  Dimetacrine  Dosulepin  Doxepin  Imipramine  Lofepramine  Maprotiline  Melitracen  Nitroxazepine  Nortriptyline  Noxiptiline  Opipramol  Pipofezine  Protriptyline  Trimipramine |
| Other Antidepressants | Bupropion  Buspirone  Maprotiline  Mianserin  Mirtazapine  Nefazodone  Reboxetine  Setiptiline  Teniloxazine  Trazodone  Viloxazine |
| Monoamine Oxidase Inhibitors (MAOIs) | Bifemelane  Caroxazone  Isocarboxazid  Metralindole  Moclobemide  Moclobemide  Phenelzine  Pirlindole  Segeline  Toloxatone  Tranylcypromine  Rasagiline |
| Antipsychotics | Amisulpride  Aripiprazole  Asenapine  Brexpiprazole  Clozapine  Lurasidone  Olanzapine  Quetiapine  Risperidone  Trifluoperazine  Ziprasidone |
| Psychostimulants | Lisdexamfetamine (Vyvanse)  Dextroamphetamine (Dexedrine)  Amphetamine/dextroamphetamine (Adderall)  Methylphenidate (Concerta, Foquest, Ritalin)  Atomoxetine (Strattera) |
| Other | Ondansetron  Metoclopramide  Tramadol  Methadone  Buspirone  Triptans (sumatripan, rizatriptan, naratriptan, eletriptan, donitriptan, almotriptan, frovatriptan, avitriptan, zolmitriptan)  Tryptophan  Linezolid  Dextromethorphan  Meperidine |

The following medications and substances must be held as indicated below.

**Medication/substance**: Instructions for use during study participation

1. **Alcohol:** No alcohol use permitted 14 hours prior to psilocybin dose, until 24 hours post-dose.
2. **Benzodiazepines, hypnotics, mood stabilizers:** Benzodiazepines, hypnotics, and mood stabilizers must be held 12 hours prior to psilocybin dose and may be resumed 12 hours post-dose.
3. **Cannabis (marijuana, weed):** Participants will be asked about their cannabis use at screening. For participants who have used cannabis within the past 12 months, cannabis use will be assessed at each scheduled study visit as part of the concomitant medication assessment. No cannabis use is permitted for 1 week prior and 1 week after the psilocybin dose.
4. **Steroids:** Steroids use is not permitted for 2 weeks before to 2 weeks after psilocybin dose.
5. **Vaccinations:** Vaccine status will be assessed at each scheduled study visit as part of the concomitant medication assessment. No vaccinations are permitted 7 days prior and 7 days after psilocybin dose.
6. **Other:** S-Adenosyl methionine (SAM-e), 5-Hydroxytryptophan (5-HTP), St. John’s Wort are not permitted for 1 week prior and 1 week after the psilocybin dose.

Please note this is not an exhaustive list. Every participant will be individually assessed and their medical history will be thoroughly reviewed during the screening visit to ensure there are no medications and substances side effects.

###### **4.4. Rescue Medications**

Regardless of the fact that there haven't been any reports of their use in well-reported clinical studies using oral psilocybin, medications need to be accessible for the treatment of severe psychosis, agitation, or symptomatic hypertension. These supplies are often two-dose units of lorazepam and/or diazepam, as well as risperidone or another orally disintegrating antipsychotic. We shall also screen all patients for potential contraindications to rescue drugs prior to administration (e.g., allergies, asthma, recent use of contraindicated medications). During and after the psilocybin session, rescue medications may be administered. In the case of acute psychological distress, benzodiazepine anxiolytics are the preferred pharmacological intervention (e.g., medications such as lorazepam or alprazolam that have a rapid onset, a short time until peak plasma concentration, and a short duration of therapeutic action; the oral route is preferred because intravenous injection procedures may exacerbate the participant's anxiety). Antipsychotic medications (for example, risperidone) should be provided in case an adverse reaction develops into severe psychosis. If the participant develops acute anxiety or psychotic symptoms that require pharmacological intervention, he or she will stay on site until the symptoms have completely resolved (overnight if required). When the physician believes that the participant's condition has stabilized, he or she may be discharged. The participant must be accompanied home by a family member, friend, or chaperone.

Management of Mild to Moderate Treatment Emergent Hypertension:

- Asymptomatic with blood pressure (BP) > 180/100:
  - Reassure the participant, ensure lights are dimmed, tilt head of bed up by 15 degrees and continue to monitor
  - Increase blood pressure measurement frequency to every 15 minutes until BP has partly normalized (sBP = 100-159; dBP = 60-99)
- Asymptomatic with BP persisting at >180/100 for >30 minutes (e.g., 2 repeat readings):
  - Administer captopril* 12.5mg PO/SL x 1 with an MD order
  - Increase blood pressure measurement frequency to every 15 minutes until BP has partly normalized (sBP = 100-159; dBP = 60-99)
- Asymptomatic with BP persisting at > 180/100 for >60 minutes post-dose, despite administering first captopril dose:
  - Consider transfer to emergency department
  - Administer 2nd dose of captopril 12.5mg x 1 with doctor’s order

Management of Severe Treatment Emergent Hypertension:

- BP > 200/110 for > 15 minutes:
  - Consider potential transfer to emergency department
  - Administer captopril 25 mg PO/SL x 1
  - Call 911 immediately for patients experiencing symptoms of a hypertensive crisis (e.g., chest pain, shortness of breath) or hypertensive encephalopathy (e.g., sudden severe headache, visual disturbances, seizures, diminished consciousness, or focal neurological deficits)

*Note: If there are contraindications to captopril, substitute for hydralazine 10 mg PO.

###### **4.5. ​​Women of Childbearing Potential and Acceptable Contraceptive Methods**

A woman is defined as being of childbearing potential from menarche until becoming postmenopausal, unless permanently sterilized (i.e., hysterectomy, bilateral salpingectomy, or bilateral oophorectomy). A woman is considered to be postmenopausal and not of childbearing potential after a minimum of 12 months without menstruation.

The following methods of contraception are considered to be highly effective if used properly:

- Combined estrogen- and progestogen-containing hormonal contraception associated with inhibition of ovulation (oral, intravaginal, or transdermal)
- Progestogen-only hormonal contraception associated with inhibition of ovulation (oral, injectable, or implantable)
- Intrauterine device
- Intrauterine hormone-releasing system
- Bilateral tubal occlusion and litigation
- Vasectomized partner
- Sexual abstinence

Please note that two forms of contraception are required with any barrier method or oral hormones (ie.g., condom plus diaphragm, condom or diaphragm plus spermicide, oral hormonal contraceptives plus spermicide or condom).

Periodic abstinence (i.e., calendar, symptothermal, or post-ovulation methods) is not an acceptable form of contraception for this study.

These methods of contraception also apply to partners of male participants.

The investigator and each participant will determine the appropriate method of contraception for the participant during the study, which will be documented at screening. If a participant or the partner of a male participant becomes pregnant during the study, the investigator must notify the sponsor immediately after the pregnancy is confirmed, as described in the ‘Exposure in Utero During Clinical Studies’ section.

###### **4.6. Exposure in Utero During Clinical Studies**

If a participant, or a male participant’s partner, becomes pregnant within 30 days of receiving the psilocybin, the sponsor must be notified. Once made aware of a pregnancy, the investigator or a designated representative must report it within five days of knowledge using the CRF. Reporting after the follow-up visit or early termination (ET) is done voluntarily by the investigator.

Reports regarding the pregnancy of a male participant’s partner should be identified using the participant’s randomisation number, initials, and date of birth.

###### **4.7. Auditing**

For this feasibility trial there will be no pre-specified independent audit. Representatives of the St. Michael's Hospital including the St. Michael's Hospital research ethics board (REB) may look at the study records and at personal health information to verify that the information collected for the study is correct and to make sure the study is following proper laws and guidelines.

###### **5. Investigational Product Management**

###### **5.1. Formulation**

Mydecine Innovations Group Inc. manufactured psilocybin capsules.

###### **5.2. Storage**

Psilocybin will be maintained at room temperature in a locked secure location at the site in accordance with regulations. The study personnel who will have access to the psilocybin inventory will be defined and recorded.

###### **6. Ethics and Responsibilities**

###### **6.1. Good Clinical Practice**

The trial will be conducted in accordance with this study protocol and in keeping with Health Canada/ICH Good Clinical Practices (GCP) guidelines. This study will also be conducted in accordance with the 2013 version of the World Medical Association Declaration of Helsinki guidelines.

###### **6.2. Data and Safety Monitoring**

Safety data will be reported to the psilocybin manufacturer.

###### **6.3. Institutional Review Board/Independent Ethics Committee**

Conduct of the study must be approved by an appropriately constituted institutional review board (IRB). Approval is required for the study protocol, protocol amendments, written informed consent forms, consent form updates that are proposed for use in the trial, participant information sheets to be provided to participants, and subject recruitment procedures (e.g., advertisements), as outlined by the Good Clinical Practice (GCP) guidelines. The IRB will also obtain the Investigator’s Brochure, available safety information, the investigator’s current curriculum vitae and/or documentation evidencing qualifications, and any other documents that the IRB may need.

###### **6.4. Informed Consent**

Participants will receive all information required by Health Canada/ICH guidelines, and must be informed that they may withdraw from the study at any time. Individuals must provide written, signed, and dated informed consent to participate in the study. Participants will not be coerced to complete study procedures. The study coordinator will provide them with information about the purpose of the study. The participants will be given sufficient time to read the informed consent form and ask any questions before deciding to participate. Participants who consent to participate in the study will then be asked to sign an informed consent form (ICF). Participants will sign the informed consent as the first step of the screening visit, before doing any study related activities. Additionally, at this visit, participants will be given the credentials of a dummy email account (an email address and password) created by the study team, which they will use to login to the Oura App (if applicable).

The specific consent process is as follows:

1. Informed consent must be obtained prior to any study-specific procedure being conducted on the patient.
2. Obtaining personal written informed consent:
   1. The information is to be given to the subject in written format, the ICF
3. Verify that the ICF given to the potential subject is the most recent REB-approved version of the consent for that clinical trial.
4. The ICF can be first presented to the patient by the study coordinator or other qualified personnel.
   1. The potential subject must be given ample time to read and understand the ICF.
   2. Study staff will then return and discuss the ICF with the potential patient, making sure to highlight important areas of the consent such as:
      1. The purpose of the study.
      2. Explain how many subjects will be involved.
      3. Discuss the duration of the study.
      4. Explain how many visits the subject will make.
      5. Discuss procedures; study intervention, device.
      6. Risks and possible benefits.
      7. Responsibilities of the investigator and subject.
      8. Any compensation to the subject.
      9. Notification of any other treatment available for the condition.
      10. That the subject has the right to withdraw.
      11. Confidentiality.

4. Promote the exchange of information with the subject and encourage any questions.

5. The principal investigator (or a co-investigator) should then join the potential subject and the subject is then given an additional opportunity to ask questions.

6. The ICF will then be signed by the subject and study staff presenting the ICF.

- 1. The original ICF will be kept separate from any documentation

containing the subject’s study code.

- 1. A copy of the ICF will be given to the subject.

###### **6.5. Protocol Deviations**

All protocol deviations will be assessed and documented on a case-by-case basis before the database lock, and deviations considered having a serious impact on the efficacy results will lead to the relevant participant being excluded from the analysis. Protocol deviations will be summarized by center and grouped into different categories, as follows: those who entered the study even though they did not satisfy the entry criteria; those who developed withdrawal criteria during the study but were not withdrawn; those who received the wrong treatment or incorrect dose; those who took any prohibited medications during the study.

###### **6.6. Protocol Amendments**

Before any changes to the study are implemented, besides those to eliminate immediate hazards to study participants, an amendment to the study will be reviewed and approved by the REB and Health Canada as applicable.

###### **6.7. Records Management**

Participant records will be distinguished using participant identification (PID) numbers.

Demographic data and active data will be collected electronically through Research Electronic Data Capture (REDCap) separately from the data collected on the Oura App [(92)](https://www.zotero.org/google-docs/?YeAogr). Personal information will be stored at Unity Health Toronto. Demographic and active data will be sent to and stored on a password-protected server maintained by Unity Health Toronto and will be linked by a study participant ID for final analysis. Access to this data will only be granted to study personnel for data management and analysis, as well as database administrators for technical support (Christopher Ducharme – [Christopher.Ducharme@unityhealth.to](mailto:Christopher.Ducharme@unityhealth.to)).

Research Electronic Data Capture (REDCap; <https://www.project-redcap.org>) software supported by Applied Health Research Centre (AHRC) at Unity Health Toronto, will be used for data collection and overall study data management over the course of this project. REDCap is an open-source, web-based clinical data management and electronic data capture system and database. The system is developed and managed in compliance with Unity Health Toronto privacy, the Health Insurance Portability and Accountability Act, the Personal Information Protection and Electronic Documents Act, and Food and Drug Administration 21 Code of Federal Regulations Part 11 regulations, providing functions such as defined user roles and privileges, user authentication and encryption for in-transit data, de-identification of protected health information and comprehensive auditing features to record and monitor access and changes to data. This system will be used to send scheduled questionnaires to participants and store active data and data monitoring and for the query and export of datasets for statistical analysis and modeling.

Access to REDCap will be secured through a secure web portal and protected by multiple levels of authentication. A project coordinator will be assigned the project administrative privileges for study configuration, data collection management, and quality control.

Participants will use the Oura Ring wearable device to collect passive data. The device and its respective app will have a specific user ID that will be set up by the research team so that the participant remains de-identified on this platform. Data collected from the Oura Ring wearable device will be made accessible through the Oura Application Programming Interface (API) (Oura Teams). Each participant’s data will be exported and de-identified before it is stored on Unity Health Toronto servers, and the data will only be associated with a participant identification number.

The wearable device will collect various parameters related to mental health and wellness, such as heart rate variability (HRV) and the Oura Ring Activity Score. The de-identified data will be stored on Oura servers, with derived metrics extracted and stored within the Unity Health Toronto servers. No personal information will be collected through the Oura digital platform. Only specific members of the project will be able to access the data, which is transferred over the Transport Layer Security protocol layer, hence is by default encrypted by Rivest–Shamir–Adleman algorithm Asymmetric Encryption Algorithm.

The Oura Ring will collect passive and continuous data through its sensors. The raw data will be collected by the Oura app, wherefrom it will be transferred to and stored on its respective server. The digital platform will be set up to comply with de-identification of the data and protection of privacy of the participant. We will use the Oura Application Programming Interface (API) to extract available metrics from their servers and to export the data to be stored on the Unity Health Toronto secure server, with backup on a secure external hard drive located at the Interventional Psychiatry Program. No personal health or clinical information will be shared with Oura.

###### **6.8. Source Documentation**

A wide variety of original documents, data, and records will be considered as source documents in this study. Source data is all information, original records of clinical findings, observations, or other activities in a clinical trial necessary for the reconstruction and evaluation of the trial. Source data are contained in source documents.

Source data include:

REB Documents

- All REB correspondences are documented
- The study staff is REB approved prior to performing any study procedures
- AEs and deviations are reported to the REB per current guidelines and stored appropriately
- All versions of the REB protocols and informed consent forms are on file

Informed Consent

- Ensure that participant identification is not recorded on the ICF (i.e., no participant ID)
- There is documentation that the participant is given a copy of the consent form
- The participant and study representative signed and dated the consent form for themself
- The participant initialed and dated all appropriate pages on the ICF

###### **6.9. Participant Protection**

Participants will not be placed at any risk as a result of the study. Information obtained will be

maintained in a secure and confidential fashion. No participant will be coerced and/or placed

under duress to complete study procedures.

###### **6.10. Confidentiality**

The confidentiality of the data collected and the identity of the individuals participating in this study will be strictly maintained. All files pertaining to subjects in the study will be assigned a unique ID. Personal identifiable information (PII), such as name, address, telephone number, email address, and date-of-birth, will not be accessible to anyone who is not authorized in the study conduct. Only the research coordinator who needs to have direct communications with the participants are authorized to access the PII. Source documents will always be kept in a locked filing cabinet to limit access, and in the case of electronic source documents, files will be password-protected and saved in a secure server. However, our CRFs will not contain any personal health information. Only the unique ID will be recorded in the CRF, and if the subject name appears on any other document (e.g., laboratory report), it must be de-identified and replaced with the unique ID from the copy of the document retained in the Trial Master File or made available for audit.

Study findings stored on a computer will be stored in accordance with Unity Health Toronto data and privacy regulations. The research coordinator, research assistants, Principal Investigator and co-investigators will have access to all source documents collected over the course of the study. Participants will be informed that representatives of other parties (including pharmaceutical companies), Research Ethics Board (REB), or regulatory authorities may inspect their records to verify that all information collected and made available for inspection will be handled in the strictest confidence and in accordance with Unity Health Toronto data and privacy regulations. The investigator will maintain a personal subject identification list (unique ID with the corresponding participant names) to enable records to be identified and retrieved.

Data collected by the Oura Ring will be de-identified on the Oura platform. Each participant’s account on Oura will be created using a dummy email address and password with an associated unique ID. This deidentification will protect participant’s privacy. Subsequently, the data will be transferred to an external encrypted and password-protected hard drive.

During data analysis, de-identified data will be provided to the analysts. This will be achieved using a two-zone approach. The two-zone approach divides study team members into two groups; identified and de-identified. As the names suggest, the identified group will have access to identified (i.e. demographic data) and de-identified information and thus is able to re-identify the data to provide data management, quality and data linkage functions (on active and passive data) whereas the de-identified group will only have access to the de-identified information. This will ensure that the data analysts of the team only have access to de-identified data but will not have access to any of the participant’s identifiers to protect participant privacy.

###### **6.11. Study Discontinuation**

In the event that the study is discontinued, subjects who have completed or who are still enrolled in the study will be notified. Any new information gained during the course of the study that might affect subjects’ safety or willingness to continue participation in the study will be communicated to participants by the study coordinator within 2 days after the principal investigator learns this information.

###### **6.12. Dissemination Policy**

###### **6.12.1. Trial Results**

The results of this trial will be published in peer-reviewed journals and presented at scientific conferences/meetings.

###### **6.12.2. Authorship**

To be eligible for authorship on any resultant publications, all potential contributors must fulfill all criteria as set forth by the International Committee of Medical Journal Editors.

###### **6.13. Declaration of Interests**

Dr. Venkat Bhat is supported by an Academic Scholar Award from the University of Toronto Department of Psychiatry and has received research support from the Canadian Institutes of Health Research, Brain & Behaviour Foundation, Ministry of Health Innovations Funds, Royal College of Physicians and Surgeons of Canada, Department of National Defence (Canada) and an investigator-initiated trials from Roche Canada.

###### **7. Statistics**

###### **7.1. General Procedures and Statistical Methods**

Feasibility outcomes will be reported with descriptive statistics (i.e., counts and proportions). A minimum of 2-3 participants per month (at our single site) will be considered a sufficient recruitment rate to indicate the feasibility of a future larger multi-site trial. We will estimate the proportions of participants adherent with the protocol, that withdraw, and for data completion with a 95% CI. A full trial with this design will be deemed feasible if the lower 95% confidence limit for the rates of adherence and data completion are **≥** 80%, and if the upper limit of 95% confidence interval for the withdrawal rate is ≤ 20%. Frequencies and proportions of AEs and serious AEs will be tabulated for the total sample for each AE type. For continuous outcomes, we will report the means and standard deviations of the score and its change from baseline until the end of treatment and over the follow-up visits. We will estimate the standard deviation of PTSD severity [CAPS-5] and its within person correlation between the baseline and the end of treatment scores. The study will examine the effect size associated with psilocybin-assisted CPT in participants with PTSD, including the durability of effects on PTSD symptoms. The main analysis will be a comparison of PTSD symptoms before and after treatment. There will be analyses of changes in symptoms of depression, psychological functioning, sleep quality and PTSD severity. In week 2, the primary endpoint assessment will take place for all subjects. An independent rater not involved in therapy will administer or collect some outcome measures, and others will be based on subjects’ self-report. Baseline will be compared to the primary end point and follow up time points. This study will provide data on the impact of psilocybin in conjunction with massed CPT. Based on the outcomes, this study will primarily detect effects due to psilocybin-assisted CPT on PTSD symptoms through calculating effect sizes and significance testing. A Wilcoxon signed rank test comparing Clinician-Administered PTSD Scale for DSM-5 (CAPS-5) scores from the baseline to the primary endpoint will be the main analysis. Durability of effects will be examined by comparing changes from baseline to the assessments at 1 until 12 weeks after the final CPT session (day 7) for each PTSD subject using Friedman test, which is a non-parametric test method for repeated measures. Effects on depression symptoms, psychological functioning, trauma-related beliefs and sleep quality will also be assessed for exploratory purposes. Changes in these measures will be compared from baseline to primary endpoint for participants using Wilcoxon signed rank tests. Analyses will examine changes from baseline up to week 13 using Friedman tests. Effect sizes for the psilocybin-assisted CPT will be calculated at the primary endpoint and follow-up time points.

Passive data is effective at identifying behaviors and trends in activity but is poor in measuring people's internal states, motivation, and attitude, whereas active data is the opposite. Developing a methodology for integrating the two data sources can mitigate their respective weaknesses. The passive data capturing activity, sleep and physiological data collected by the Oura Ring will be correlated and fused with the active data collected by the clinical data. The motivation to fuse active and passive data includes the cross-validation and improvement of measurements, the explanation of human behavior, and novel opportunities to improve representative models in experimental settings. Techniques such as feature extraction, dimension reduction, feature relevance estimation for ranking, statistical analysis, and data fusion will be used on the passive and active.

The active data will serve as a ground truth to validate the passive data from the wearable device for monitoring anxiety and depression. We will use generalized machine learning models to detect and predict distress among participants and develop personalized time series machine learning models for personalized application. This will involve creating predictive models based on a participant's data and implementing the model suited for the participant. This proposal will be an iterative process. To mitigate missing data, suitable filtering and encoders may be used to interpolate and predict the expected data accordingly.

During the analysis, we will extract features from the passive physiological data for better representation. Trends, classifications, and relationships will be determined using different machine learning techniques. Implementations of pre-processing, feature extraction and machine learning will be conducted in a numerical program for efficient and powerful processing that will achieve impactful results.

###### **7.2. Sample Size**

Because the anticipated effect size is unknown, a formal sample size calculation cannot be completed. For this trial, it is anticipated that 15 participants will be recruited.

###### **8. Personal Digital Phenotype Analysis and Generation**

Our goal is to have a systematic investigation of DPP with an emphasis on robustness for long-term utility. We envision the DPP to be represented as a multi-dimensional vector composed of passive and active data, where each profile is unique to one another. To do so, we propose a Statistics, Information Theory, and Data-driven (SID) pipeline to develop the foundation of the DPP. The proposed SID pipeline is encapsulated in Figure 3.

## **
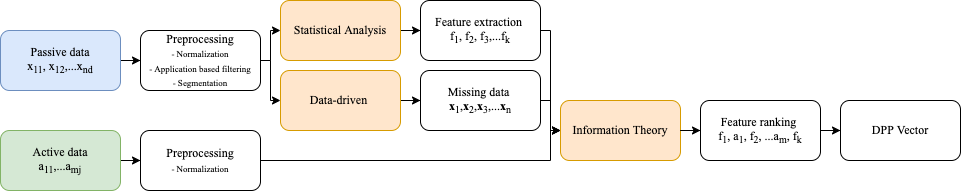
**

**Figure 3.** Block diagram representation of the proposed SID pipeline.

Digital Phenotype Profile (DPP) will be created through the use of SID, and will be a representation for each individual user (93). Once the Digital Phenotype Profile (DPP) is created, we enhance the analysis by proposing the personalized digital phenotype profile (pDPP) where we develop individual models for each person by incorporating their physiological data together with their self-reported affective states collected from questionnaires. Once the personal profile/model has been constructed, physiological data (such as that collected from the Oura Ring) can be used to provide insight into a person's affective state, even without any active data. The personalized digital phenotype profile (pDPP) will be used for long-term utility where we monitor the changes in physical and behavioral health of an individual participant. The application of personalized digital phenotype profile (pDPP) could allow for insights into nurses' mental well-being in a non-intrusive way that can improve mental health care and responses to stress.

###### **9. Interpretation of the Personal Digital Phenotype**

Our goal is to have a systematic investigation of personalized digital phenotype profile (pDPP) with an emphasis on robustness for long-term utility. We envision the pDPP to be represented as a multi-dimensional vector composed of passive and active data, where each profile is unique to one another. We will use analytical techniques such as robust principal component analysis (RPCA) to represent the data. In addition, robust principal component analysis (RPCA) decomposes the matrices to low- and sparse-rank representation where the low-rank representation can be considered the outliers and sparse-rank can be considered as the concise representation of the personalized digital phenotype profile (pDPP) vector. We plan to develop an algorithm to analyze the low-rank representation for anomaly detection where new additional samples are added (passive or active data) or when the personalized digital phenotype profile (pDPP) baseline varies past an accepted threshold, an alert of potential change in physical and behavioral health can be announced.

###### **10. Study Administration**

###### **10.1 Key Contacts**

**Principal Investigator**

Dr. Venkat Bhat, MD MSc FRCPC DABPN

St. Michael's Hospital and the University of Toronto

416-360-4000 Ext. 76404

[Venkat.bhat@utoronto.ca](mailto:Venkat.bhat@utoronto.ca)

**Co-Principal Investigator**

Dr. Candice Monson, PhD

Professor of Psychology at Toronto Metropolitan University

416-979-5000 Ext. 556209

[candice.monson@torontomu.ca](mailto:candice.monson@torontomu.ca)

**Co-Investigators**

Dr. Perry Menzies

St. Michael's Hospital and the University of Toronto

416-864-3085

[perry.menzies@unityhealth.to](mailto:perry.menzies@unityhealth.to)

Dr. Rakesh Jetly, MD FRCPC

Associate Professor of Psychiatry at Dalhousie University and University of Ottawa

613-945-6817

[rakesh@drjetly.com](mailto:rakesh@drjetly.com)

Dr. Wendy Lou PhD

Professor of Biostatistics, Dalla Lana School of Public Health, University of Toronto

416-946-7804

[wendy.lou@utoronto.ca](mailto:wendy.lou@utoronto.ca)

Dr. Sridhar (Sri) Krishnan PhD, PEng, FCAE

Professor of Engineering, Signal Analysis Research Group, Toronto Metropolitan University

416-979-5000 Ext. 554931

[krishnan@ryerson.ca](mailto:krishnan@ryerson.ca)

###### **10.2. Funding**

This study will be conducted with internal sources of funding. Psilocybin in kind contribution of Mydecine Innovations Group Inc.

###### **10.3. Roles and Responsibilities**

###### **10.3.1. Sponsor and Funder**

The study funder has no role in study design, collection, management, analysis, and interpretation of data; writing of the report; and the decision to submit the report for publication.

###### **10.3.2. Trial Committees**

The steering committee of the trial will consist of the investigators.

**References**

1. Bryant, Richard A. “Post‐traumatic Stress Disorder: A State‐of‐the‐art Review of Evidence and Challenges.” *World Psychiatry* 18, no. 3 (October 2019): 259–69.<https://doi.org/10.1002/wps.20656>.
2. Yehuda, Rachel, Charles W. Hoge, Alexander C. McFarlane, Eric Vermetten, Ruth A. Lanius, Caroline M. Nievergelt, Stevan E. Hobfoll, Karestan C. Koenen, Thomas C. Neylan, and Steven E. Hyman. “Post-Traumatic Stress Disorder.” *Nature Reviews Disease Primers* 1, no. 1 (October 8, 2015): 15057.<https://doi.org/10.1038/nrdp.2015.57>.
3. Goldstein, Risë B., Sharon M. Smith, S. Patricia Chou, Tulshi D. Saha, Jeesun Jung, Haitao Zhang, Roger P. Pickering, W. June Ruan, Boji Huang, and Bridget F. Grant. “The Epidemiology of DSM-5 Posttraumatic Stress Disorder in the United States: Results from the National Epidemiologic Survey on Alcohol and Related Conditions-III.” *Social Psychiatry and Psychiatric Epidemiology* 51, no. 8 (August 2016): 1137–48.<https://doi.org/10.1007/s00127-016-1208-5>.
4. Scott, K. M., K. C. Koenen, A. King, M. V. Petukhova, J. Alonso, E. J. Bromet, R. Bruffaerts, et al. “Post-Traumatic Stress Disorder Associated with Sexual Assault among Women in the WHO World Mental Health Surveys.” *Psychological Medicine* 48, no. 1 (January 2018): 155–67.<https://doi.org/10.1017/S0033291717001593>.
5. Brewin, Chris R. “Episodic Memory, Perceptual Memory, and Their Interaction: Foundations for a Theory of Posttraumatic Stress Disorder.” *Psychological Bulletin* 140, no. 1 (2014): 69–97.<https://doi.org/10.1037/a0033722>.
6. Bisson, Jonathan I, Sarah Cosgrove, Catrin Lewis, and Neil P Roberts. “Post-Traumatic Stress Disorder.” *BMJ*, November 26, 2015, h6161.<https://doi.org/10.1136/bmj.h6161>.
7. Cahill, Shawn P., and Kristin Pontoski. “Post-Traumatic Stress Disorder and Acute Stress Disorder I: Their Nature and Assessment Considerations.” *Psychiatry (Edgmont (Pa.: Township))* 2, no. 4 (April 2005): 14–25.
8. Hudson, Peter. “Effective Treatments for PTSD: Practice Guidelines from the International Society for Traumatic Stress Studies.” *British Journal of Guidance & Counselling* 39, no. 2 (April 2011): 194–95.<https://doi.org/10.1080/03069885.2010.550798>.
9. Fletcher, Susan, Mark Creamer, and David Forbes. “Preventing Post Traumatic Stress Disorder: Are Drugs the Answer?” *Australian & New Zealand Journal of Psychiatry* 44, no. 12 (December 2010): 1064–71.<https://doi.org/10.3109/00048674.2010.509858>.
10. Korte, Kristina J., Nicholas P. Allan, Daniel F. Gros, and Ron Acierno. “Differential Treatment Response Trajectories in Individuals with Subclinical and Clinical PTSD.” *Journal of Anxiety Disorders* 38 (March 2016): 95–101.<https://doi.org/10.1016/j.janxdis.2016.01.006>.
11. Lewis, Catrin, Neil P. Roberts, Martin Andrew, Elise Starling, and Jonathan I. Bisson. “Psychological Therapies for Post-Traumatic Stress Disorder in Adults: Systematic Review and Meta-Analysis.” *European Journal of Psychotraumatology* 11, no. 1 (2020): 1729633.<https://doi.org/10.1080/20008198.2020.1729633>.
12. Bryant, Richard A., Alexander C. McFarlane, Derrick Silove, Meaghan L. O’Donnell, David Forbes, and Mark Creamer. “The Lingering Impact of Resolved PTSD on Subsequent Functioning.” *Clinical Psychological Science* 4, no. 3 (May 2016): 493–98.<https://doi.org/10.1177/2167702615598756>.
13. Goetter, Elizabeth M., Allyson M. Blackburn, Cory Stasko, Yijie Han, Lauren H. Brenner, Simon Lejeune, Kaloyan S. Tanev, Thomas J. Spencer, and Edward C. Wright. “Comparative Effectiveness of Prolonged Exposure and Cognitive Processing Therapy for Military Service Members in an Intensive Treatment Program.” *Psychological Trauma: Theory, Research, Practice, and Policy* 13, no. 6 (September 2021): 632–40.<https://doi.org/10.1037/tra0000956>.
14. Foa, Edna B., Carmen P. McLean, Yinyin Zang, David Rosenfield, Elna Yadin, Jeffrey S. Yarvis, Jim Mintz, et al. “Effect of Prolonged Exposure Therapy Delivered Over 2 Weeks vs 8 Weeks vs Present-Centered Therapy on PTSD Symptom Severity in Military Personnel: A Randomized Clinical Trial.” *JAMA* 319, no. 4 (January 23, 2018): 354.<https://doi.org/10.1001/jama.2017.21242>.
15. Ehlers, Anke, David M. Clark, Ann Hackmann, Nick Grey, Sheena Liness, Jennifer Wild, John Manley, Louise Waddington, and Freda McManus. “Intensive Cognitive Therapy for PTSD: A Feasibility Study.” *Behavioural and Cognitive Psychotherapy* 38, no. 4 (July 2010): 383–98.<https://doi.org/10.1017/S1352465810000214>.
16. Galovski, Tara E., Kimberly B. Werner, Terri L. Weaver, Kris L. Morris, Katherine A. Dondanville, John Nanney, Rachel Wamser-Nanney, Gina McGlinchey, Catherine B. Fortier, and Katherine M. Iverson. “Massed Cognitive Processing Therapy for Posttraumatic Stress Disorder in Women Survivors of Intimate Partner Violence.” *Psychological Trauma: Theory, Research, Practice, and Policy* 14, no. 5 (July 2022): 769–79.<https://doi.org/10.1037/tra0001100>.
17. Brown, Lily A., Joshua D. Clapp, Joshua J. Kemp, Jeffrey S. Yarvis, Katherine A. Dondanville, Brett T. Litz, Jim Mintz, et al. “The Pattern of Symptom Change during Prolonged Exposure Therapy and Present-Centered Therapy for PTSD in Active Duty Military Personnel.” *Psychological Medicine* 49, no. 12 (September 2019): 1980–89.<https://doi.org/10.1017/S0033291718002714>.
18. Kovacevic, Merdijana, Karyna Bravo, and Philip Held. “Posttraumatic Stress Disorder and Depression Residual Symptoms among Veterans and Service Members Who Completed a 3-Week Cognitive Processing Therapy-Based Intensive Treatment Program.” *Psychological Trauma: Theory, Research, Practice, and Policy*, December 19, 2022.<https://doi.org/10.1037/tra0001197>.
19. Held, Philip, Merdijana Kovacevic, Kelsey Petrey, Enya A. Meade, Sarah Pridgen, Mauricio Montes, Brianna Werner, et al. “Treating Posttraumatic Stress Disorder at Home in a Single Week Using 1‐week Virtual Massed Cognitive Processing Therapy.” *Journal of Traumatic Stress* 35, no. 4 (August 2022): 1215–25.<https://doi.org/10.1002/jts.22831>.
20. Held, Philip, Alyson K. Zalta, Dale L. Smith, Jenna M. Bagley, Victoria L. Steigerwald, Randy A. Boley, Michelle Miller, Michael B. Brennan, Rebecca Van Horn, and Mark H. Pollack. “Maintenance of Treatment Gains up to 12-Months Following a Three-Week Cognitive Processing Therapy-Based Intensive PTSD Treatment Programme for Veterans.” *European Journal of Psychotraumatology* 11, no. 1 (August 12, 2020): 1789324.<https://doi.org/10.1080/20008198.2020.1789324>.
21. Hofmann, A., R. Heim, A. Brack, and H. Kobel. “Psilocybin, ein psychotroper Wirkstoff aus dem mexikanischen RauschpilzPsilocybe mexicana Heim.” *Experientia* 14, no. 3 (March 1958): 107–9.<https://doi.org/10.1007/BF02159243>.
22. Passie, Torsten, Juergen Seifert, Udo Schneider, and Hinderk M. Emrich. “The Pharmacology of Psilocybin.” *Addiction Biology* 7, no. 4 (October 2002): 357–64.<https://doi.org/10.1080/1355621021000005937>.
23. Carter, Olivia L., David C. Burr, John D. Pettigrew, Guy M. Wallis, Felix Hasler, and Franz X. Vollenweider. “Using Psilocybin to Investigate the Relationship between Attention, Working Memory, and the Serotonin 1A and 2A Receptors.” *Journal of Cognitive Neuroscience* 17, no. 10 (October 1, 2005): 1497–1508.<https://doi.org/10.1162/089892905774597191>.
24. Carhart-Harris, Robin L, and Guy M Goodwin. “The Therapeutic Potential of Psychedelic Drugs: Past, Present, and Future.” *Neuropsychopharmacology* 42, no. 11 (October 2017): 2105–13.<https://doi.org/10.1038/npp.2017.84>.
25. Goldberg, Simon B., Brian T. Pace, Christopher R. Nicholas, Charles L. Raison, and Paul R. Hutson. “The Experimental Effects of Psilocybin on Symptoms of Anxiety and Depression: A Meta-Analysis.” *Psychiatry Research* 284 (February 2020): 112749.<https://doi.org/10.1016/j.psychres.2020.112749>.
26. Yoon, Hyo Shin, Chung Hwan Cho, Myeong Sik Yun, Sung Jae Jang, Hyun Ju You, Jun-hyeong Kim, Dohyun Han, et al. “Akkermansia Muciniphila Secretes a Glucagon-like Peptide-1-Inducing Protein That Improves Glucose Homeostasis and Ameliorates Metabolic Disease in Mice.” *Nature Microbiology* 6, no. 5 (April 5, 2021): 563–73.<https://doi.org/10.1038/s41564-021-00880-5>.
27. Romeo, Bruno, Laurent Karila, Catherine Martelli, and Amine Benyamina. “Efficacy of Psychedelic Treatments on Depressive Symptoms: A Meta-Analysis.” *Journal of Psychopharmacology* 34, no. 10 (October 2020): 1079–85.<https://doi.org/10.1177/0269881120919957>.
28. Mithoefer, Michael C, Charles S Grob, and Timothy D Brewerton. “Novel Psychopharmacological Therapies for Psychiatric Disorders: Psilocybin and MDMA.” *The Lancet Psychiatry* 3, no. 5 (May 2016): 481–88.<https://doi.org/10.1016/S2215-0366(15)00576-3>.
29. Kočárová, Rita, Jiří Horáček, and Robin Carhart-Harris. “Does Psychedelic Therapy Have a Transdiagnostic Action and Prophylactic Potential?” *Frontiers in Psychiatry* 12 (July 19, 2021): 661233.<https://doi.org/10.3389/fpsyt.2021.661233>.
30. Carhart-Harris, Robin, Bruna Giribaldi, Rosalind Watts, Michelle Baker-Jones, Ashleigh Murphy-Beiner, Roberta Murphy, Jonny Martell, Allan Blemings, David Erritzoe, and David J. Nutt. “Trial of Psilocybin versus Escitalopram for Depression.” *New England Journal of Medicine* 384, no. 15 (April 15, 2021): 1402–11.<https://doi.org/10.1056/NEJMoa2032994>.
31. Davis, Alan K., Frederick S. Barrett, Darrick G. May, Mary P. Cosimano, Nathan D. Sepeda, Matthew W. Johnson, Patrick H. Finan, and Roland R. Griffiths. “Effects of Psilocybin-Assisted Therapy on Major Depressive Disorder: A Randomized Clinical Trial.” *JAMA Psychiatry* 78, no. 5 (May 1, 2021): 481.<https://doi.org/10.1001/jamapsychiatry.2020.3285>.
32. Goodwin, Guy M., Scott T. Aaronson, Oscar Alvarez, Peter C. Arden, Annie Baker, James C. Bennett, Catherine Bird, et al. “Single-Dose Psilocybin for a Treatment-Resistant Episode of Major Depression.” *New England Journal of Medicine* 387, no. 18 (November 3, 2022): 1637–48.<https://doi.org/10.1056/NEJMoa2206443>.
33. Rotz, Robin von, Eva M. Schindowski, Johannes Jungwirth, Anna Schuldt, Nathalie M. Rieser, Katharina Zahoranszky, Erich Seifritz, et al. “Single-Dose Psilocybin-Assisted Therapy in Major Depressive Disorder: A Placebo-Controlled, Double-Blind, Randomised Clinical Trial.” *EClinicalMedicine* 56 (February 2023): 101809.<https://doi.org/10.1016/j.eclinm.2022.101809>.
34. Ross, Stephen, Anthony Bossis, Jeffrey Guss, Gabrielle Agin-Liebes, Tara Malone, Barry Cohen, Sarah E Mennenga, et al. “Rapid and Sustained Symptom Reduction Following Psilocybin Treatment for Anxiety and Depression in Patients with Life-Threatening Cancer: A Randomized Controlled Trial.” *Journal of Psychopharmacology* 30, no. 12 (December 2016): 1165–80.<https://doi.org/10.1177/0269881116675512>.
35. Griffiths, Roland R, Matthew W Johnson, Michael A Carducci, Annie Umbricht, William A Richards, Brian D Richards, Mary P Cosimano, and Margaret A Klinedinst. “Psilocybin Produces Substantial and Sustained Decreases in Depression and Anxiety in Patients with Life-Threatening Cancer: A Randomized Double-Blind Trial.” *Journal of Psychopharmacology* 30, no. 12 (December 2016): 1181–97.<https://doi.org/10.1177/0269881116675513>.
36. Bogenschutz, Michael P., Stephen Ross, Snehal Bhatt, Tara Baron, Alyssa A. Forcehimes, Eugene Laska, Sarah E. Mennenga, et al. “Percentage of Heavy Drinking Days Following Psilocybin-Assisted Psychotherapy vs Placebo in the Treatment of Adult Patients With Alcohol Use Disorder: A Randomized Clinical Trial.” *JAMA Psychiatry* 79, no. 10 (October 1, 2022): 953.<https://doi.org/10.1001/jamapsychiatry.2022.2096>.
37. Bird, Catherine I. V., Nadav L. Modlin, and James J. H. Rucker. “Psilocybin and MDMA for the Treatment of Trauma-Related Psychopathology.” *International Review of Psychiatry* 33, no. 3 (April 3, 2021): 229–49.<https://doi.org/10.1080/09540261.2021.1919062>.
38. Krediet, Erwin, Tijmen Bostoen, Joost Breeksema, Annette van Schagen, Torsten Passie, and Eric Vermetten. “Reviewing the Potential of Psychedelics for the Treatment of PTSD.” *International Journal of Neuropsychopharmacology* 23, no. 6 (June 24, 2020): 385–400.<https://doi.org/10.1093/ijnp/pyaa018>.
39. Anderson, Brian T, Alicia Danforth, Prof Robert Daroff, Christopher Stauffer, Eve Ekman, Gabrielle Agin-Liebes, Alexander Trope, et al. “Psilocybin-Assisted Group Therapy for Demoralized Older Long-Term AIDS Survivor Men: An Open-Label Safety and Feasibility Pilot Study.” *EClinicalMedicine* 27 (October 2020): 100538.<https://doi.org/10.1016/j.eclinm.2020.100538>.
40. Watts, Rosalind, Camilla Day, Jacob Krzanowski, David Nutt, and Robin Carhart-Harris. “Patients’ Accounts of Increased ‘Connectedness’ and ‘Acceptance’ After Psilocybin for Treatment-Resistant Depression.” *Journal of Humanistic Psychology* 57, no. 5 (September 2017): 520–64.<https://doi.org/10.1177/0022167817709585>.
41. Wolff, Max, Ricarda Evens, Lea J. Mertens, Michael Koslowski, Felix Betzler, Gerhard Gründer, and Henrik Jungaberle. “Learning to Let Go: A Cognitive-Behavioral Model of How Psychedelic Therapy Promotes Acceptance.” *Frontiers in Psychiatry* 11 (February 21, 2020): 5.<https://doi.org/10.3389/fpsyt.2020.00005>.
42. Zeifman, Richard J., Anne C. Wagner, Ros Watts, Hannes Kettner, Lea J. Mertens, and Robin L. Carhart-Harris. “Post-Psychedelic Reductions in Experiential Avoidance Are Associated With Decreases in Depression Severity and Suicidal Ideation.” *Frontiers in Psychiatry* 11 (August 7, 2020): 782.<https://doi.org/10.3389/fpsyt.2020.00782>.
43. Catlow, Briony J., Shijie Song, Daniel A. Paredes, Cheryl L. Kirstein, and Juan Sanchez-Ramos. “Effects of Psilocybin on Hippocampal Neurogenesis and Extinction of Trace Fear Conditioning.” *Experimental Brain Research* 228, no. 4 (August 2013): 481–91.<https://doi.org/10.1007/s00221-013-3579-0>.
44. Kraehenmann, Rainer, Katrin H. Preller, Milan Scheidegger, Thomas Pokorny, Oliver G. Bosch, Erich Seifritz, and Franz X. Vollenweider. “Psilocybin-Induced Decrease in Amygdala Reactivity Correlates with Enhanced Positive Mood in Healthy Volunteers.” *Biological Psychiatry* 78, no. 8 (October 2015): 572–81.<https://doi.org/10.1016/j.biopsych.2014.04.010>.
45. Raut, Sanket B., Padmaja A. Marathe, Liza van Eijk, Rajaraman Eri, Manoj Ravindran, David M. Benedek, Robert J. Ursano, Juan J. Canales, and Luke R. Johnson. “Diverse Therapeutic Developments for Post-Traumatic Stress Disorder (PTSD) Indicate Common Mechanisms of Memory Modulation.” *Pharmacology & Therapeutics* 239 (November 2022): 108195.<https://doi.org/10.1016/j.pharmthera.2022.108195>.
46. Sessa, Ben. “MDMA and PTSD Treatment.” *Neuroscience Letters* 649 (May 2017): 176–80.<https://doi.org/10.1016/j.neulet.2016.07.004>.
47. Wagner, Anne C., Rachel E. Liebman, Ann T. Mithoefer, Michael C. Mithoefer, and Candice M. Monson. “Relational and Growth Outcomes Following Couples Therapy With MDMA for PTSD.” *Frontiers in Psychiatry* 12 (June 28, 2021): 702838.<https://doi.org/10.3389/fpsyt.2021.702838>.
48. Mitchell, Jennifer M., Michael Bogenschutz, Alia Lilienstein, Charlotte Harrison, Sarah Kleiman, Kelly Parker-Guilbert, Marcela Ot’alora G., et al. “MDMA-Assisted Therapy for Severe PTSD: A Randomized, Double-Blind, Placebo-Controlled Phase 3 Study.” *Nature Medicine* 27, no. 6 (June 2021): 1025–33.<https://doi.org/10.1038/s41591-021-01336-3>.
49. Mithoefer, Michael C, Ann T Mithoefer, Allison A Feduccia, Lisa Jerome, Mark Wagner, Joy Wymer, Julie Holland, et al. “3,4-Methylenedioxymethamphetamine (MDMA)-Assisted Psychotherapy for Post-Traumatic Stress Disorder in Military Veterans, Firefighters, and Police Officers: A Randomised, Double-Blind, Dose-Response, Phase 2 Clinical Trial.” *The Lancet Psychiatry* 5, no. 6 (June 2018): 486–97.<https://doi.org/10.1016/S2215-0366(18)30135-4>.
50. Brown, Randall T., Christopher R. Nicholas, Nicholas V. Cozzi, Michele C. Gassman, Karen M. Cooper, Daniel Muller, Chantelle D. Thomas, et al. “Pharmacokinetics of Escalating Doses of Oral Psilocybin in Healthy Adults.” *Clinical Pharmacokinetics* 56, no. 12 (December 2017): 1543–54.<https://doi.org/10.1007/s40262-017-0540-6>.
51. Hasler, F., D. Bourquin, R. Brenneisen, T. Bär, and F.X. Vollenweider. “Determination of Psilocin and 4-Hydroxyindole-3-Acetic Acid in Plasma by HPLC-ECD and Pharmacokinetic Profiles of Oral and Intravenous Psilocybin in Man.” *Pharmaceutica Acta Helvetiae* 72, no. 3 (June 1997): 175–84.<https://doi.org/10.1016/S0031-6865(97)00014-9>.
52. Lindenblatt, Hiltrud, Edda Krämer, Petra Holzmann-Erens, Euphrosyne Gouzoulis-Mayfrank, and Karl-Artur Kovar. “Quantitation of Psilocin in Human Plasma by High-Performance Liquid Chromatography and Electrochemical Detection: Comparison of Liquid–Liquid Extraction with Automated on-Line Solid-Phase Extraction.” *Journal of Chromatography B: Biomedical Sciences and Applications* 709, no. 2 (May 1998): 255–63.<https://doi.org/10.1016/S0378-4347(98)00067-X>.
53. Hasler, Felix, Daniel Bourquin, Rudolf Brenneisen, and Franz X Vollenweider. “Renal Excretion Profiles of Psilocin Following Oral Administration of Psilocybin: A Controlled Study in Man.” *Journal of Pharmaceutical and Biomedical Analysis* 30, no. 2 (September 2002): 331–39.<https://doi.org/10.1016/S0731-7085(02)00278-9>.
54. Dinis-Oliveira, Ricardo Jorge. “Metabolism of Psilocybin and Psilocin: Clinical and Forensic Toxicological Relevance.” *Drug Metabolism Reviews* 49, no. 1 (January 2, 2017): 84–91.<https://doi.org/10.1080/03602532.2016.1278228>.
55. Tylš, Filip, Tomáš Páleníček, and Jiří Horáček. “Psilocybin – Summary of Knowledge and New Perspectives.” *European Neuropsychopharmacology* 24, no. 3 (March 2014): 342–56.<https://doi.org/10.1016/j.euroneuro.2013.12.006>.
56. Bailey, Christopher R., Elisabeth Cordell, Sean M. Sobin, and Alexander Neumeister. “Recent Progress in Understanding the Pathophysiology of Post-Traumatic Stress Disorder: Implications for Targeted Pharmacological Treatment.” *CNS Drugs* 27, no. 3 (March 2013): 221–32.<https://doi.org/10.1007/s40263-013-0051-4>.
57. Béïque, Jean-Claude, Mays Imad, Ljiljana Mladenovic, Jay A. Gingrich, and Rodrigo Andrade. “Mechanism of the 5-Hydroxytryptamine 2A Receptor-Mediated Facilitation of Synaptic Activity in Prefrontal Cortex.” *Proceedings of the National Academy of Sciences of the United States of America* 104, no. 23 (June 5, 2007): 9870–75.<https://doi.org/10.1073/pnas.0700436104>.
58. Zanos, Panos, Ruin Moaddel, Patrick J. Morris, Lace M. Riggs, Jaclyn N. Highland, Polymnia Georgiou, Edna F. R. Pereira, et al. “Ketamine and Ketamine Metabolite Pharmacology: Insights into Therapeutic Mechanisms.” *Pharmacological Reviews* 70, no. 3 (July 2018): 621–60.<https://doi.org/10.1124/pr.117.015198>.
59. Griffiths, Roland R., Matthew W. Johnson, William A. Richards, Brian D. Richards, Una McCann, and Robert Jesse. “Psilocybin Occasioned Mystical-Type Experiences: Immediate and Persisting Dose-Related Effects.” *Psychopharmacology* 218, no. 4 (December 2011): 649–65.<https://doi.org/10.1007/s00213-011-2358-5>.
60. Carhart-Harris, Robin L, Mark Bolstridge, James Rucker, Camilla M J Day, David Erritzoe, Mendel Kaelen, Michael Bloomfield, et al. “Psilocybin with Psychological Support for Treatment-Resistant Depression: An Open-Label Feasibility Study.” *The Lancet Psychiatry* 3, no. 7 (July 2016): 619–27.<https://doi.org/10.1016/S2215-0366(16)30065-7>.
61. Bender, David, and David J. Hellerstein. “Assessing the Risk–Benefit Profile of Classical Psychedelics: A Clinical Review of Second-Wave Psychedelic Research.” *Psychopharmacology* 239, no. 6 (June 2022): 1907–32.<https://doi.org/10.1007/s00213-021-06049-6>.
62. Geyer, M, and F Vollenweider. “Serotonin Research: Contributions to Understanding Psychoses.” *Trends in Pharmacological Sciences* 29, no. 9 (September 2008): 445–53.<https://doi.org/10.1016/j.tips.2008.06.006>.
63. Studerus, Erich, Michael Kometer, Felix Hasler, and Franz X Vollenweider. “Acute, Subacute and Long-Term Subjective Effects of Psilocybin in Healthy Humans: A Pooled Analysis of Experimental Studies.” *Journal of Psychopharmacology* 25, no. 11 (November 2011): 1434–52.<https://doi.org/10.1177/0269881110382466>.
64. MacLean, Katherine A., Matthew W. Johnson, and Roland R. Griffiths. “Mystical Experiences Occasioned by the Hallucinogen Psilocybin Lead to Increases in the Personality Domain of Openness.” *Journal of Psychopharmacology (Oxford, England)* 25, no. 11 (November 2011): 1453–61.<https://doi.org/10.1177/0269881111420188>.
65. Gukasyan, Natalie, Alan K Davis, Frederick S Barrett, Mary P Cosimano, Nathan D Sepeda, Matthew W Johnson, and Roland R Griffiths. “Efficacy and Safety of Psilocybin-Assisted Treatment for Major Depressive Disorder: Prospective 12-Month Follow-Up.” *Journal of Psychopharmacology* 36, no. 2 (February 2022): 151–58.<https://doi.org/10.1177/02698811211073759>.
66. Moshe, Isaac, Yannik Terhorst, Kennedy Opoku Asare, Lasse Bosse Sander, Denzil Ferreira, Harald Baumeister, David C. Mohr, and Laura Pulkki-Råback. “Predicting Symptoms of Depression and Anxiety Using Smartphone and Wearable Data.” *Frontiers in Psychiatry* 12 (January 28, 2021): 625247.<https://doi.org/10.3389/fpsyt.2021.625247>.
67. Altini, Marco, and Hannu Kinnunen. “The Promise of Sleep: A Multi-Sensor Approach for Accurate Sleep Stage Detection Using the Oura Ring.” *Sensors* 21, no. 13 (June 23, 2021): 4302.<https://doi.org/10.3390/s21134302>.
68. Chee, Nicholas IYN, Shohreh Ghorbani, Hosein Aghayan Golkashani, Ruth LF Leong, Ju Lynn Ong, and Michael WL Chee. “Multi-Night Validation of a Sleep Tracking Ring in Adolescents Compared with a Research Actigraph and Polysomnography.” *Nature and Science of Sleep* Volume 13 (February 2021): 177–90.<https://doi.org/10.2147/NSS.S286070>.
69. Weathers, Frank W., Michelle J. Bovin, Daniel J. Lee, Denise M. Sloan, Paula P. Schnurr, Danny G. Kaloupek, Terence M. Keane, and Brian P. Marx. “The Clinician-Administered PTSD Scale for DSM–5 (CAPS-5): Development and Initial Psychometric Evaluation in Military Veterans.” *Psychological Assessment* 30, no. 3 (March 2018): 383–95.<https://doi.org/10.1037/pas0000486>.
70. Bovin, Michelle J., Brian P. Marx, Frank W. Weathers, Matthew W. Gallagher, Paola Rodriguez, Paula P. Schnurr, and Terence M. Keane. “Psychometric Properties of the PTSD Checklist for Diagnostic and Statistical Manual of Mental Disorders–Fifth Edition (PCL-5) in Veterans.” *Psychological Assessment* 28, no. 11 (November 2016): 1379–91.<https://doi.org/10.1037/pas0000254>.
71. Armour, Cherie, Jack Tsai, Tory A. Durham, Ruby Charak, Tracey L. Biehn, Jon D. Elhai, and Robert H. Pietrzak. “Dimensional Structure of DSM-5 Posttraumatic Stress Symptoms: Support for a Hybrid Anhedonia and Externalizing Behaviors Model.” *Journal of Psychiatric Research* 61 (February 2015): 106–13.<https://doi.org/10.1016/j.jpsychires.2014.10.012>.
72. Kroenke, Kurt, Robert L. Spitzer, and Janet B. W. Williams. “The PHQ-9: Validity of a Brief Depression Severity Measure.” *Journal of General Internal Medicine* 16, no. 9 (September 2001): 606–13.<https://doi.org/10.1046/j.1525-1497.2001.016009606.x>.
73. Martin, Alexandra, Winfried Rief, Antje Klaiberg, and Elmar Braehler. “Validity of the Brief Patient Health Questionnaire Mood Scale (PHQ-9) in the General Population.” *General Hospital Psychiatry* 28, no. 1 (January 2006): 71–77.<https://doi.org/10.1016/j.genhosppsych.2005.07.003>.
74. Spitzer RL, Kroenke K, Williams JBW, Löwe B. A brief measure for assessing generalized anxiety disorder: the GAD-7. Arch Intern Med [Internet]. 2006 May 22;166(10):1092–7. Available from: <http://dx.doi.org/10.1001/archinte.166.10.1092>.
75. “Validity of the Dissociative Experiences Scale in Screening for Multiple Personality Disorder: A Multicenter Study.” *American Journal of Psychiatry* 150, no. 7 (July 1993): 1030–36.<https://doi.org/10.1176/ajp.150.7.1030>.
76. Buysse, D. J., C. F. Reynolds, T. H. Monk, C. C. Hoch, A. L. Yeager, and D. J. Kupfer. “Quantification of Subjective Sleep Quality in Healthy Elderly Men and Women Using the Pittsburgh Sleep Quality Index (PSQI).” *Sleep* 14, no. 4 (August 1991): 331–38.
77. Smyth, Carole. “The Pittsburgh Sleep Quality Index (PSQI).” *Journal of Gerontological Nursing* 25, no. 12 (December 1999): 10–10.<https://doi.org/10.3928/0098-9134-19991201-10>.
78. Topp, Christian Winther, Søren Dinesen Østergaard, Susan Søndergaard, and Per Bech. “The WHO-5 Well-Being Index: A Systematic Review of the Literature.” *Psychotherapy and Psychosomatics* 84, no. 3 (2015): 167–76.<https://doi.org/10.1159/000376585>.
79. Verhofstadt, Lesley L., Ann Buysse, Yves Rosseel, and Olivier J. Peene. “Confirming the Three-Factor Structure of the Quality of Relationships Inventory within Couples.” *Psychological Assessment* 18, no. 1 (March 2006): 15–21.<https://doi.org/10.1037/1040-3590.18.1.15>.
80. Bovin, Michelle J., Shimrit K. Black, Paola Rodriguez, Carole A. Lunney, Sarah E. Kleiman, Frank W. Weathers, Paula P. Schnurr, James Spira, Terence M. Keane, and Brian P. Marx. “Development and Validation of a Measure of PTSD-Related Psychosocial Functional Impairment: The Inventory of Psychosocial Functioning.” *Psychological Services* 15, no. 2 (May 2018): 216–29.<https://doi.org/10.1037/ser0000220>.
81. Vogt, Dawne S., Jillian C. Shipherd, and Patricia A. Resick. “Posttraumatic Maladaptive Beliefs Scale: Evolution of the Personal Beliefs and Reactions Scale.” *Assessment* 19, no. 3 (September 2012): 308–17.<https://doi.org/10.1177/1073191110376161>.
82. Gámez, Wakiza, Michael Chmielewski, Roman Kotov, Camilo Ruggero, Nadia Suzuki, and David Watson. “The Brief Experiential Avoidance Questionnaire: Development and Initial Validation.” *Psychological Assessment* 26, no. 1 (March 2014): 35–45.<https://doi.org/10.1037/a0034473>.
83. Grégoire, Simon, Joel Gagnon, Lise Lachance, Rebecca Shankland, Frédérick Dionne, Ilios Kotsou, Jean-Louis Monestès, Jaci L. Rolffs, and Ronald D. Rogge. “Validation of the English and French Versions of the Multidimensional Psychological Flexibility Inventory Short Form (MPFI-24).” *Journal of Contextual Behavioral Science* 18 (October 2020): 99–110.<https://doi.org/10.1016/j.jcbs.2020.06.004>.
84. Herrero, Rocío, M^a^ Dolores Vara, Marta Miragall, Cristina Botella, Azucena García-Palacios, Heleen Riper, Annet Kleiboer, and Rosa M^a^ Baños. “Working Alliance Inventory for Online Interventions-Short Form (WAI-TECH-SF): The Role of the Therapeutic Alliance between Patient and Online Program in Therapeutic Outcomes.” *International Journal of Environmental Research and Public Health* 17, no. 17 (August 25, 2020): 6169.<https://doi.org/10.3390/ijerph17176169>.
85. Kaufman, Erin A., Mengya Xia, Gregory Fosco, Mona Yaptangco, Chloe R. Skidmore, and Sheila E. Crowell. “The Difficulties in Emotion Regulation Scale Short Form (DERS-SF): Validation and Replication in Adolescent and Adult Samples.” *Journal of Psychopathology and Behavioral Assessment* 38, no. 3 (September 2016): 443–55.<https://doi.org/10.1007/s10862-015-9529-3>.
86. Raes, Filip, Elizabeth Pommier, Kristin D. Neff, and Dinska Van Gucht. “Construction and Factorial Validation of a Short Form of the Self-Compassion Scale.” *Clinical Psychology & Psychotherapy* 18, no. 3 (May 2011): 250–55.<https://doi.org/10.1002/cpp.702>.
87. Davis, Alan K, Frederick S Barrett, Sara So, Natalie Gukasyan, Thomas C Swift, and Roland R Griffiths. “Development of the Psychological Insight Questionnaire among a Sample of People Who Have Consumed Psilocybin or LSD.” *Journal of Psychopharmacology* 35, no. 4 (April 2021): 437–46.<https://doi.org/10.1177/0269881120967878>.
88. Roseman, Leor, Eline Haijen, Kelvin Idialu-Ikato, Mendel Kaelen, Rosalind Watts, and Robin Carhart-Harris. “Emotional Breakthrough and Psychedelics: Validation of the Emotional Breakthrough Inventory.” *Journal of Psychopharmacology* 33, no. 9 (September 2019): 1076–87.<https://doi.org/10.1177/0269881119855974>.
89. Studerus, Erich, Alex Gamma, and Franz X. Vollenweider. “Psychometric Evaluation of the Altered States of Consciousness Rating Scale (OAV).” Edited by Vaughan Bell. *PLoS ONE* 5, no. 8 (August 31, 2010): e12412.<https://doi.org/10.1371/journal.pone.0012412>.
90. Wolff, Max, Lea J Mertens, Marie Walter, Sören Enge, and Ricarda Evens. “The Acceptance/Avoidance-Promoting Experiences Questionnaire (APEQ): A Theory-Based Approach to Psychedelic Drugs’ Effects on Psychological Flexibility.” *Journal of Psychopharmacology* 36, no. 3 (March 2022): 387–408.<https://doi.org/10.1177/02698811211073758>.
91. Posner, Kelly, Maria A. Oquendo, Madelyn Gould, Barbara Stanley, and Mark Davies. “Columbia Classification Algorithm of Suicide Assessment (C-CASA): Classification of Suicidal Events in the FDA’s Pediatric Suicidal Risk Analysis of Antidepressants.” *The American Journal of Psychiatry* 164, no. 7 (July 2007): 1035–43.<https://doi.org/10.1176/ajp.2007.164.7.1035>.
92. Obeid, Jihad S., Catherine A. McGraw, Brenda L. Minor, José G. Conde, Robert Pawluk, Michael Lin, Janey Wang, et al. “Procurement of Shared Data Instruments for Research Electronic Data Capture (REDCap).” *Journal of Biomedical Informatics* 46, no. 2 (April 2013): 259–65.<https://doi.org/10.1016/j.jbi.2012.10.006>.
93. [Nguyen B, Nigro M, Rueda A, Krishnan S, Bhat V. Digital Phenotype Representation by Statistical and Data-Driven Approaches with Digital Health Data. ICASSP 2022 - 2022 IEEE Int Conf Acoust Speech Signal Process ICASSP 2022. Published online 2022.](https://www.zotero.org/google-docs/?broken=jfVRha)
