# Supplementary material for: Psilocybin-assisted massed cognitive processing therapy for chronic posttraumatic stress disorder: Protocol for an open-label pilot feasibility trial
Source: PLoS One. 2025 Jan 17;20(1):e0313741. doi: 10.1371/journal.pone.0313741 (PMC11741563; doi:10.1371/journal.pone.0313741)
Supplement: Attachment — Submitted filename: renamed_4f500.docx [file pone.0313741.s004.docx]

# COMMENTS – Manuscript PONE-D-24-19436

Title: “Psilocybin-Assisted Massed Cognitive Processing Therapy for Chronic Posttraumatic Stress Disorder: Protocol for an Open-Label Trial”

**Important note: This review pertains only to ‘statistical aspects’ of the study and so ‘clinical aspects’ [like medical importance, relevance of the study, ‘clinical significance and** **implication(s)’ of the whole study, etc.] are to be evaluated [should be assessed] separately/independently. Further please note that any ‘statistical review’ is generally done under the assumption that study specific methodological [as well as execution] issues are perfectly taken care of by the investigator(s). This review is not an exception to that and so does not cover clinical aspects {however, seldom comments are made only if those issues are intimately / scientifically related & intermingle with ‘statistical aspects’ of the study}. Agreed that** **‘statistical methods’ are used as just tools here, however, they are vital part of methodology [and so should be given due importance]. I look at the manuscript in/with statistical view point, other reviewer(s) look(s) at it with different angle so that in totality the review is very comprehensive. However, there should be efforts from authors side to improve (may be by taking clues from reviewer’s comments). Therefore, please do not limit the revision only (with respect) to comments made here.**

**COMMENTS:** Although there is no major flaw [study/manuscript is already of acceptable level], I have different opinion / observations/concerns regarding (very) few issues which are given below, if agreed, try to incorporate them (as this study is at planning/protocol stage only)]:

In ‘Abstract-Background’ section it is stated that “This open-label pilot study will examine the feasibility, tolerability, and preliminary efficacy of a single dose 25 mg psilocybin, combined with 1 week of massed CPT in chronic PTSD”. However, in the title “pilot / feasibility study” is not mentioned. Why? You may know that many things are often ignored (loosely looked at / evaluated) in case of ‘pilot studies’ [typically involving a small number of subjects], and that more latitude [i.e., leeway, freedom, liberty] in statistical requirements is observed/given. Nevertheless, pilot nature of the study is expected to be mentioned in the title itself.

For a pilot study it is alright to have ‘single-arm design’, [or it is alright when that is the only possibility’], however, it is very essential to keep the limitations in mind while interpreting results. This study being ‘pilot’ (feasibility study) in nature, sample size is not a big issue. However, few important methodological issues need to be very rigorous (to the maximum possible extent) followed. Very good that in section ‘5. Reporting’ you stated “Outcomes will be reported according to CONSORT reporting guidelines” and quoted reference 39 [Schulz KF, Altman DG, Moher D, CONSORT Group. CONSORT 2010 Statement: updated guidelines for reporting parallel group randomised trials. BMC Med 2010; 8: 18], however, please note that this being a ‘single-arm design’ pilot study many items are not applicable {and so keep the limitations in mind}. You may be aware of one important reference, namely: “Introducing the CONSORT extension to pilot trials: enhancing the design, conduct and reporting of pilot or feasibility trials” by Luciana P. F. Abbade, Joelcio F. Abbade and Lehana Thabane.

As said in section ‘7. Discussion’ [This open label trial is primarily interested in precise estimates of feasibility, tolerability, efficacy and safety outcome variability, in patients with chronic PTSD that will aid in the planning of a larger, sufficiently powered RCT. PTSD is a disabling and severe psychiatric disorder, and it is imperative to develop rapid-acting interventions and increase the efficacy of current front-line treatments. This open-label trial is a first step towards what may be a promising intervention for chronic PTSD. Findings from this study may help to develop psilocybin-assisted iCPT as a novel and rapid acting intervention for individuals suffering with chronic PTSD] and because you intend to use many scales [Assessments will consist of several clinical scales (CAPS-5, PCL-5, PHQ-9, GAD-7, DES-II, PSQI, QRI, WHO-5, IPF, PMBS, BEAQ, MPFI-24, WAI-SF, DERS-SF, SCF-SF, PIQ, CTQ-SF, EBI, ASC, APEQ) to measure symptoms severity and efficacy], by rightly guessing that you all (team) are likely be involved in the planning of a larger, sufficiently powered RCT subsequently and in this context, I request all authors to read the following note pasted from one standard textbook on ‘Medical Research Methodology’ [though I am sure that the authors already know these things]:

Though the measures/tools used are appropriate often times, most of them are likely to yield data that are in [at the most] ‘ordinal’ level of measurement [and not in ratio level of measurement for sure {as the score two times higher does not indicate presence of that parameter/phenomenon as double (for example, a Visual Analogue Scales VAS score or say ‘depression’ score)}]. Then application of suitable non-parametric (or distribution free) test(s) is/are indicated/advisable [even if distribution may be ‘Gaussian’ (also called ‘normal’)]. Agreed that there is/are no non-parametric test(s)/technique(s) available to be used as alternative in all situation(s), but should be used whenever/wherever they are available. Therefore, in short use suitable non-parametric test(s)/technique(s) while dealing with data that are in ‘ordinal’ level of measurement even if [despite that] the distribution may be ‘Gaussian’. Testing ‘normality’ in sample [by using any normality test(s)} is not required/desired while dealing with data that are in ‘ordinal’ level of measurement [as most of the normality tests are not valid for ‘ordinal’ data].

In section ‘4. Statistical analysis’ you have mentioned ‘non-parametric’ test [Wilcoxon signed rank test comparing CAPS-5 scores from the baseline to the primary endpoint will be the main analysis] which shows that right things are known to the team and so this a sort of reminder to keep mind.

As pointed out in ‘important note’ above “This review pertains only to ‘statistical aspects’ of the study and so ‘clinical aspects’ should be assessed separately/independently. In my opinion, to make this article finally acceptable (which is quite possible and easy), a small amount of re-vision (re-drafting) may be needed. ‘Minor revision’ is recommended .
